# Supplementary material for: IMiDs induce FAM83F degradation via an interaction with CK1α to attenuate Wnt signalling
Source: Life Sci Alliance. 2020 Dec 23;4(2):e202000804. doi: 10.26508/lsa.202000804 (PMC7768194; doi:10.26508/lsa.202000804)
Supplement: Supplementary file 1 [file LSA-2020-00804_SdataF1.pdf]

Figure 1A.

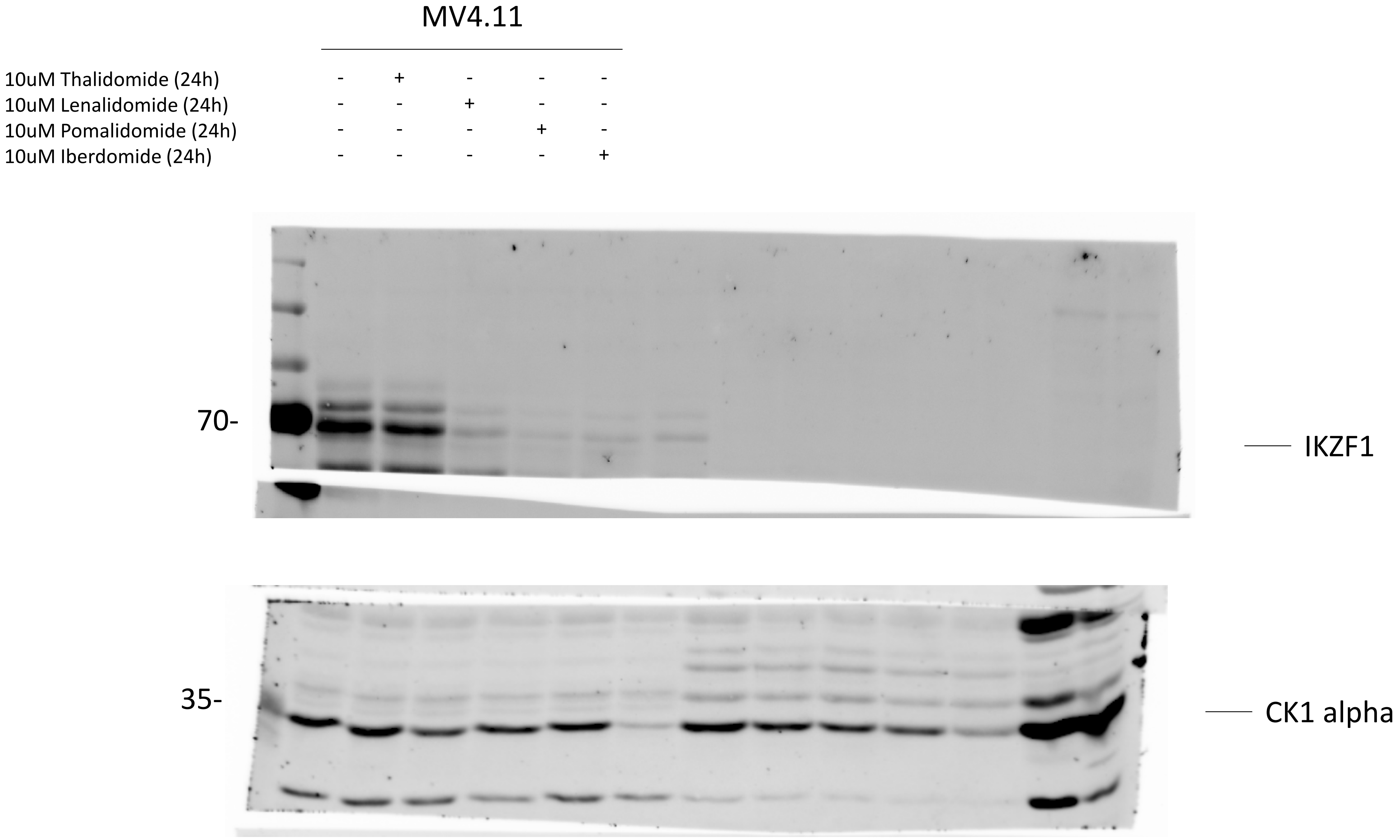
MV4.11
10uM Thalidomide (24h) - + - - -
10uM Lenalidomide (24h) - - + - -
10uM Pomalidomide (24h) - - - + -
10uM Iberdomide (24h) - - - - +
70-
IKZF1
35-
CK1 alpha

Figure 1A.

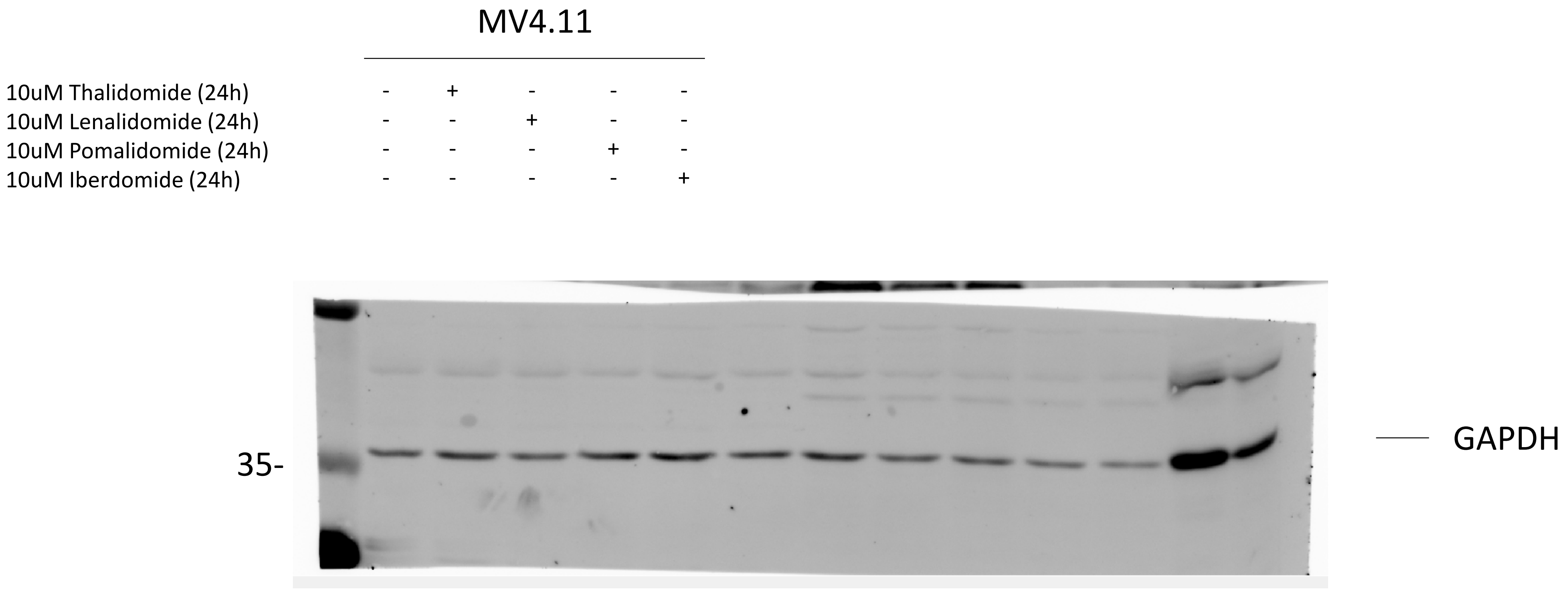
MV4.11
10uM Thalidomide (24h) - + - - -
10uM Lenalidomide (24h) - - + - -
10uM Pomalidomide (24h) - - - + -
10uM Iberdomide (24h) - - - - +
35-
GAPDH

Figure 1B.

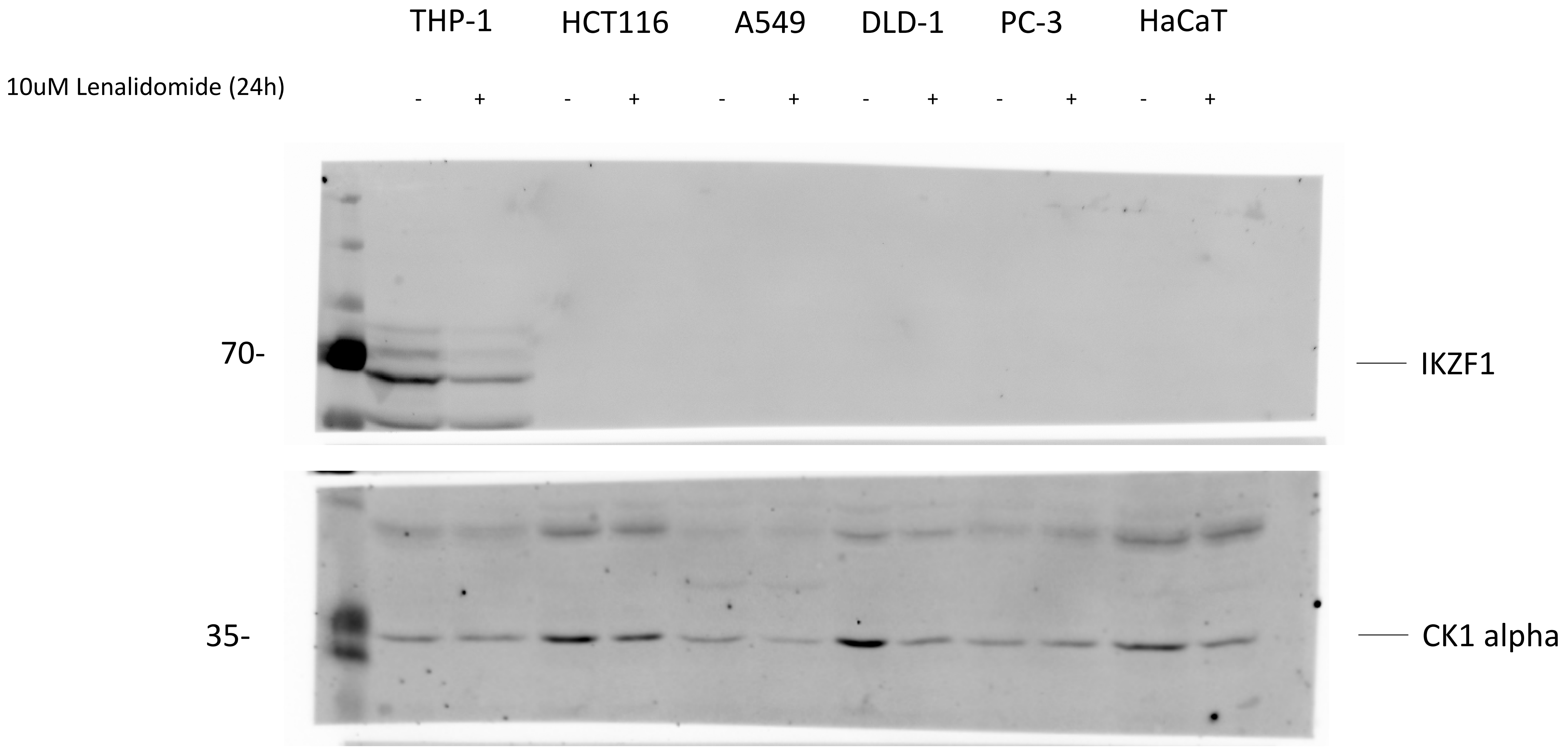
THP-1
HCT116
A549
DLD-1
PC-3
HaCaT
10uM Lenalidomide (24h)
- + - + - + - + - + - +
70-
35-
IKZF1
CK1 alpha

Figure 1B.

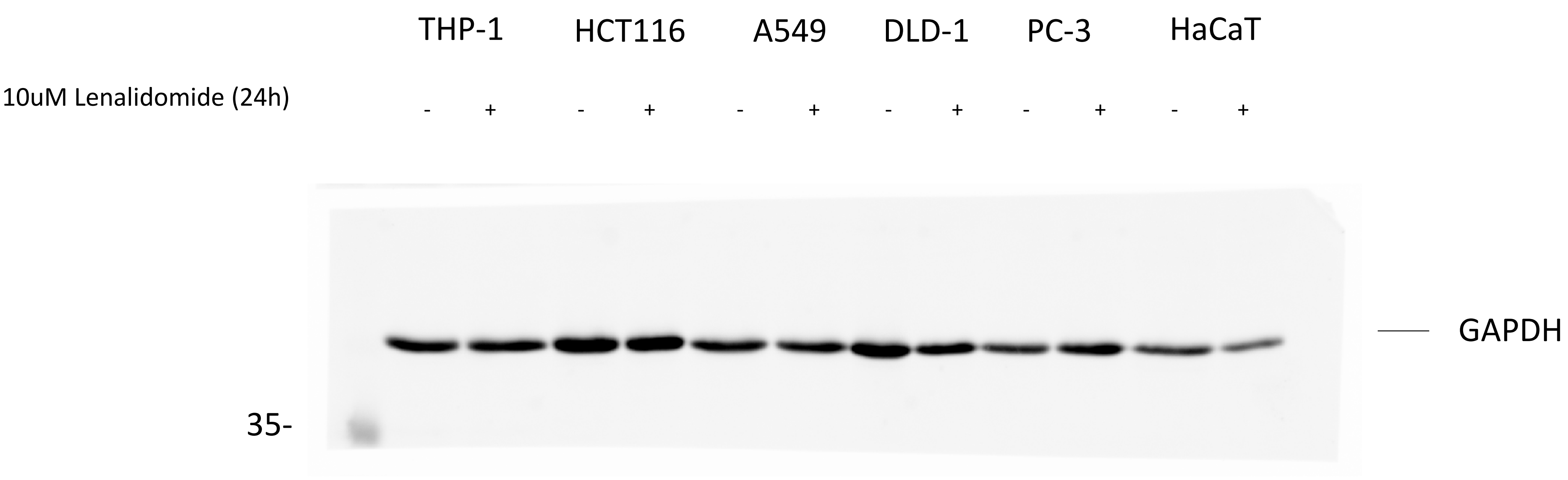
THP-1
HCT116
A549
DLD-1
PC-3
HaCaT
10uM Lenalidomide (24h)
- + - + - + - + - + - +
GAPDH
35-

Figure 1C.

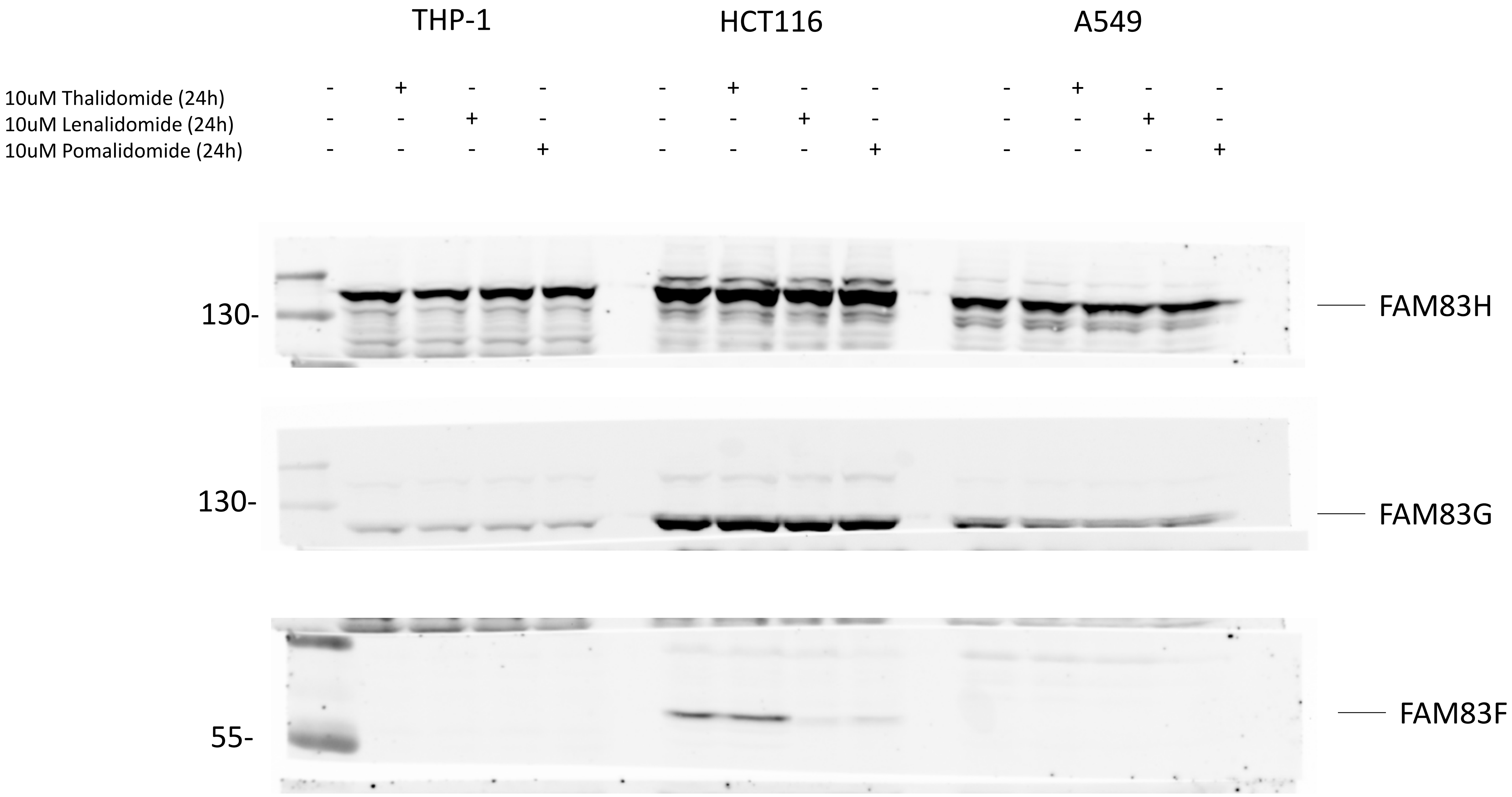
THP-1
HCT116
A549
10uM Thalidomide (24h) - + - - - + - - - + - -
10uM Lenalidomide (24h) - - + - - - + - - - + -
10uM Pomalidomide (24h) - - - + - - - + - - - +
130-
FAM83H
130-
FAM83G
55-
FAM83F

Figure 1C.

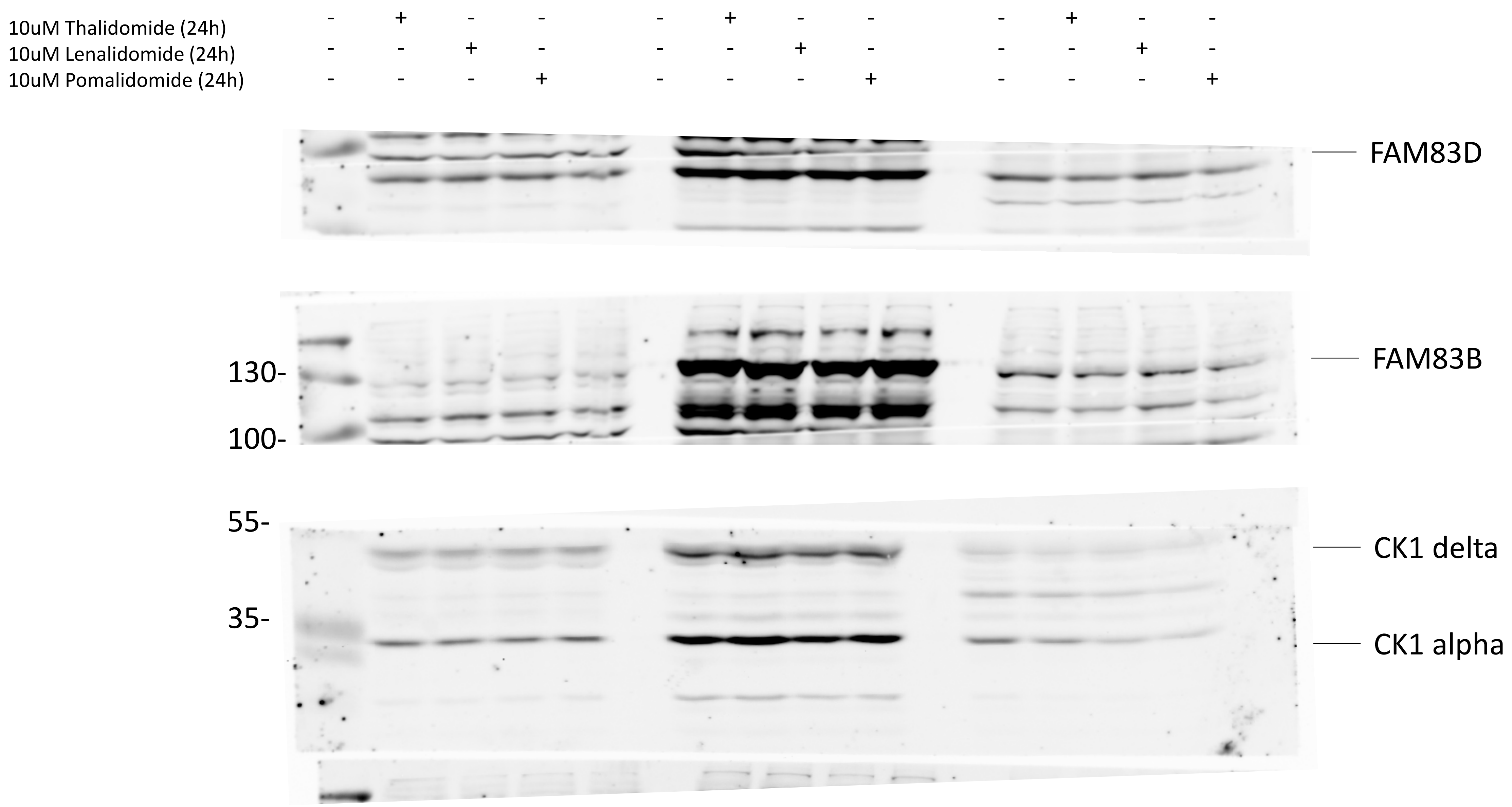
THP-1
HCT116
A549
10uM Thalidomide (24h)
10uM Lenalidomide (24h)
10uM Pomalidomide (24h)
- + - - - + - - - + - -
- - + - - - + - - - + -
- - - + - - - + - - - +
FAM83D
FAM83B
130-
100-
55-
35-
CK1 delta
CK1 alpha

Figure 1C.

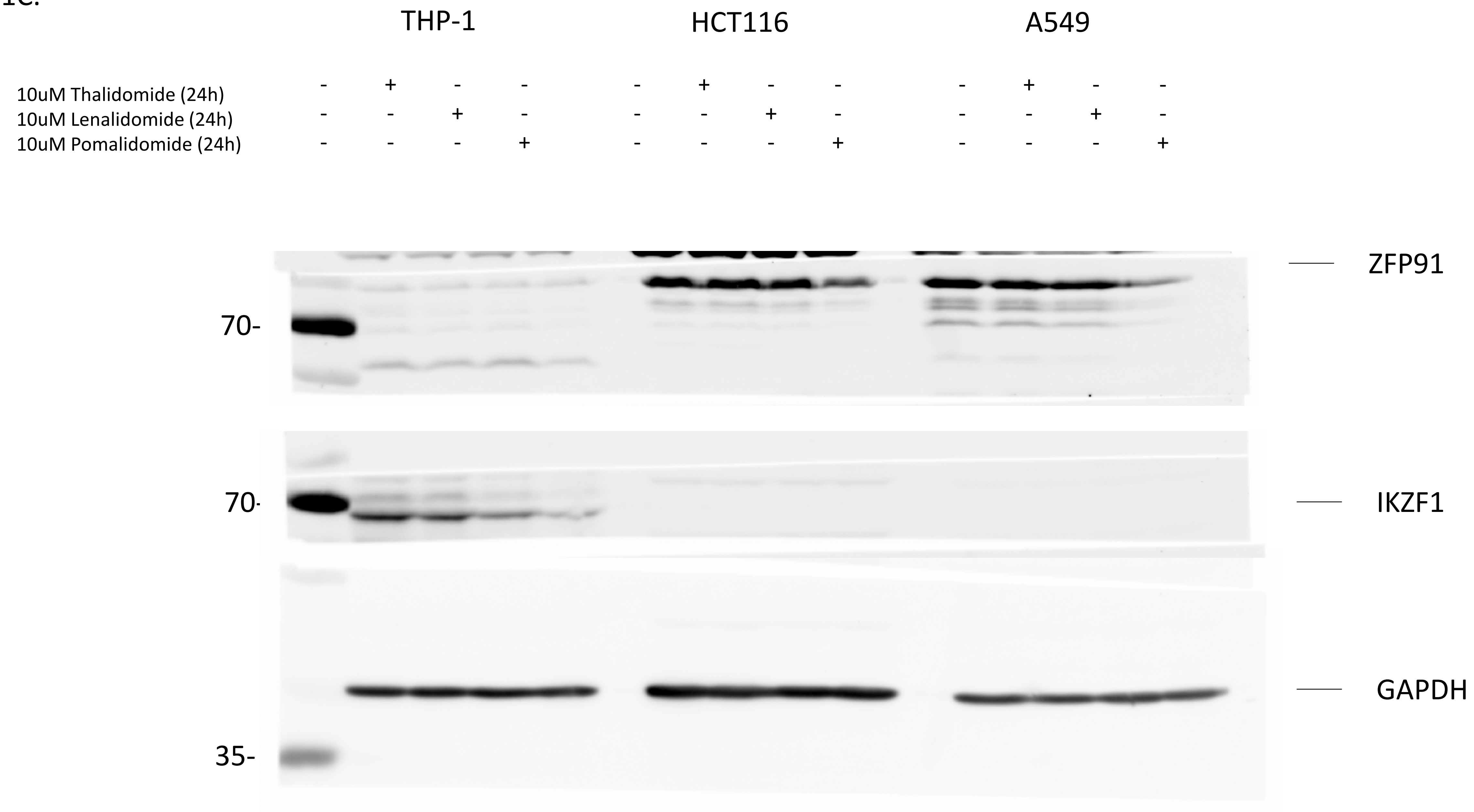
THP-1
HCT116
A549
10uM Thalidomide (24h)
10uM Lenalidomide (24h)
10uM Pomalidomide (24h)
70-
70-
35-
ZFP91
IKZF1
GAPDH

Figure 1C.

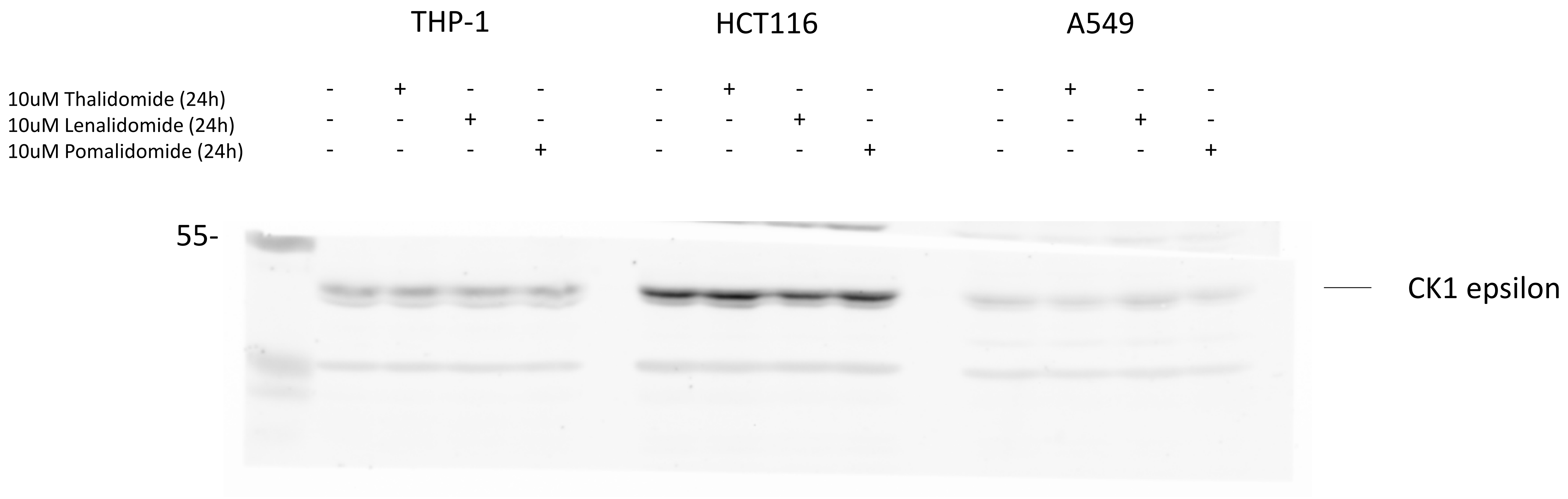
THP-1
HCT116
A549
10uM Thalidomide (24h) - + - - - + - - - + - -
10uM Lenalidomide (24h) - - + - - - + - - - + -
10uM Pomalidomide (24h) - - - + - - - + - - - +
55-
CK1 epsilon

Figure 1C.

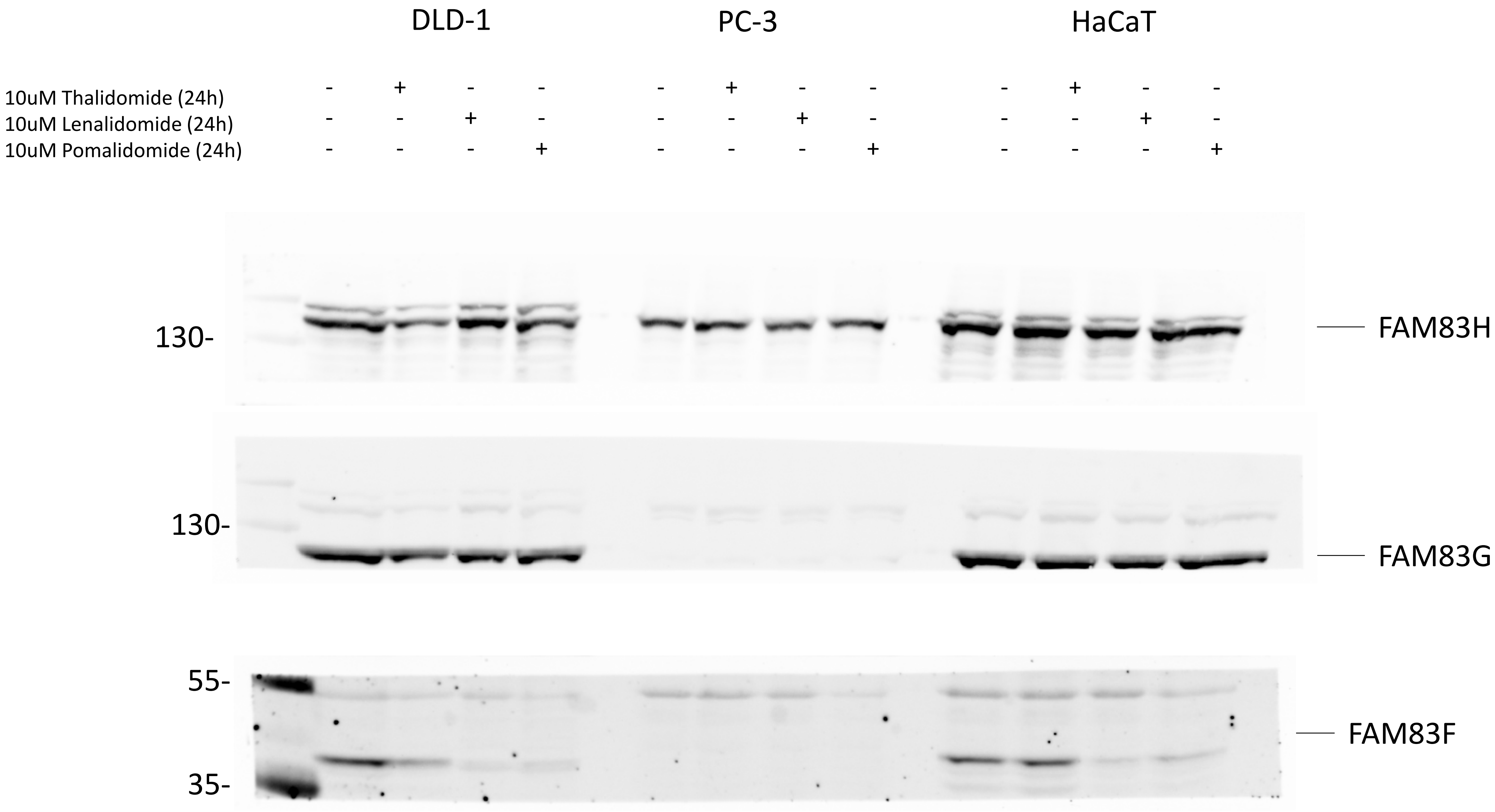
DLD-1
PC-3
HaCaT
10uM Thalidomide (24h) - + - - - + - - - + - -
10uM Lenalidomide (24h) - - + - - - + - - - + -
10uM Pomalidomide (24h) - - - + - - - + - - - +
130-
FAM83H
130-
FAM83G
55-
35-
FAM83F

Figure 1C.

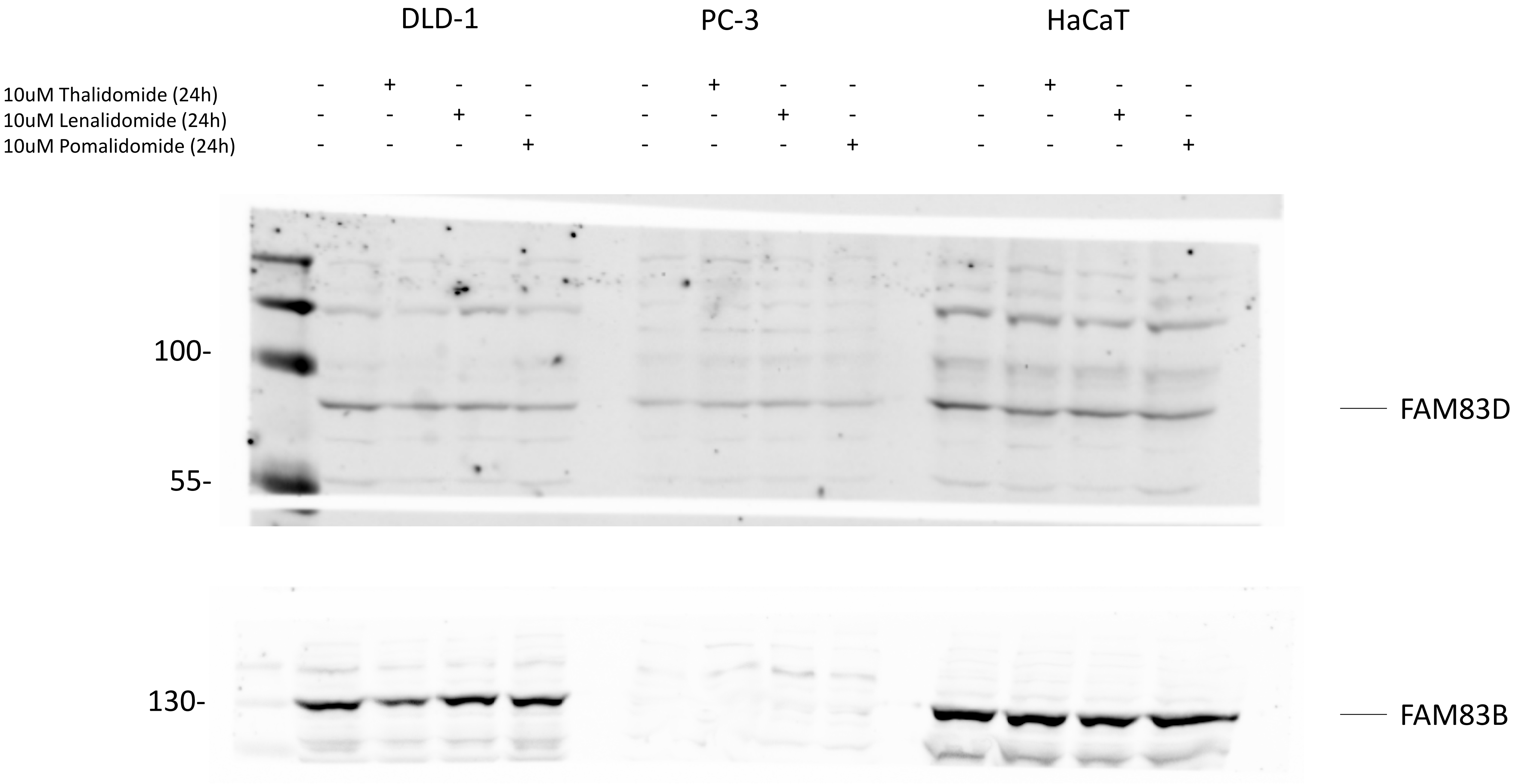

DLD-1
PC-3
HaCaT
10uM Thalidomide (24h) - + - - - + - - - + - -
10uM Lenalidomide (24h) - - + - - - + - - - + -
10uM Pomalidomide (24h) - - - + - - - + - - - +
100-
55-
FAM83D
130-
FAM83B

Figure 1C.

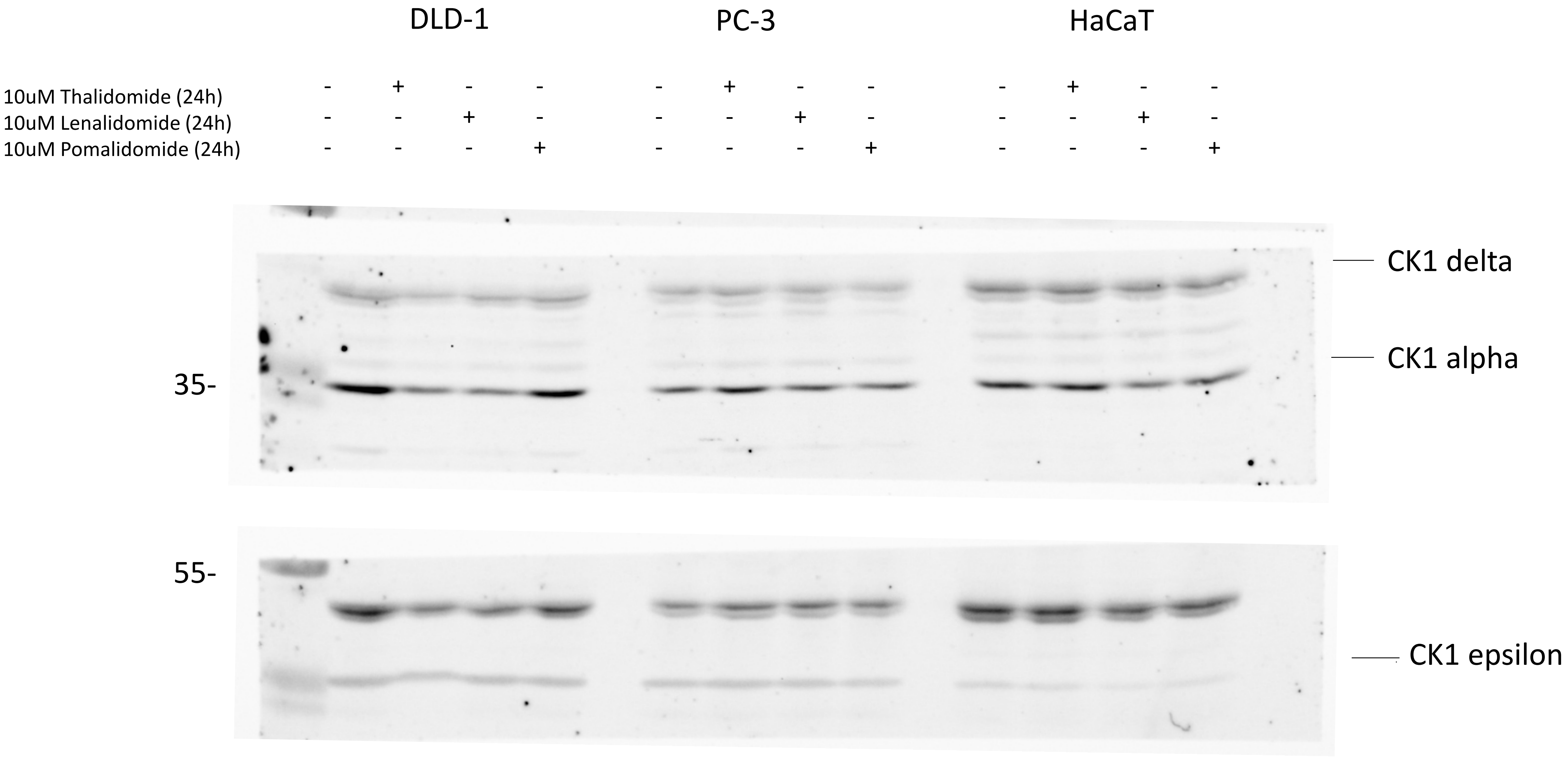
DLD-1
PC-3
HaCaT
10uM Thalidomide (24h)
10uM Lenalidomide (24h)
10uM Pomalidomide (24h)
- + - - - + - - - + - -
- - + - - - + - - - + -
- - - + - - - + - - - +
35-
55-
CK1 delta
CK1 alpha
CK1 epsilon

Figure 1C.

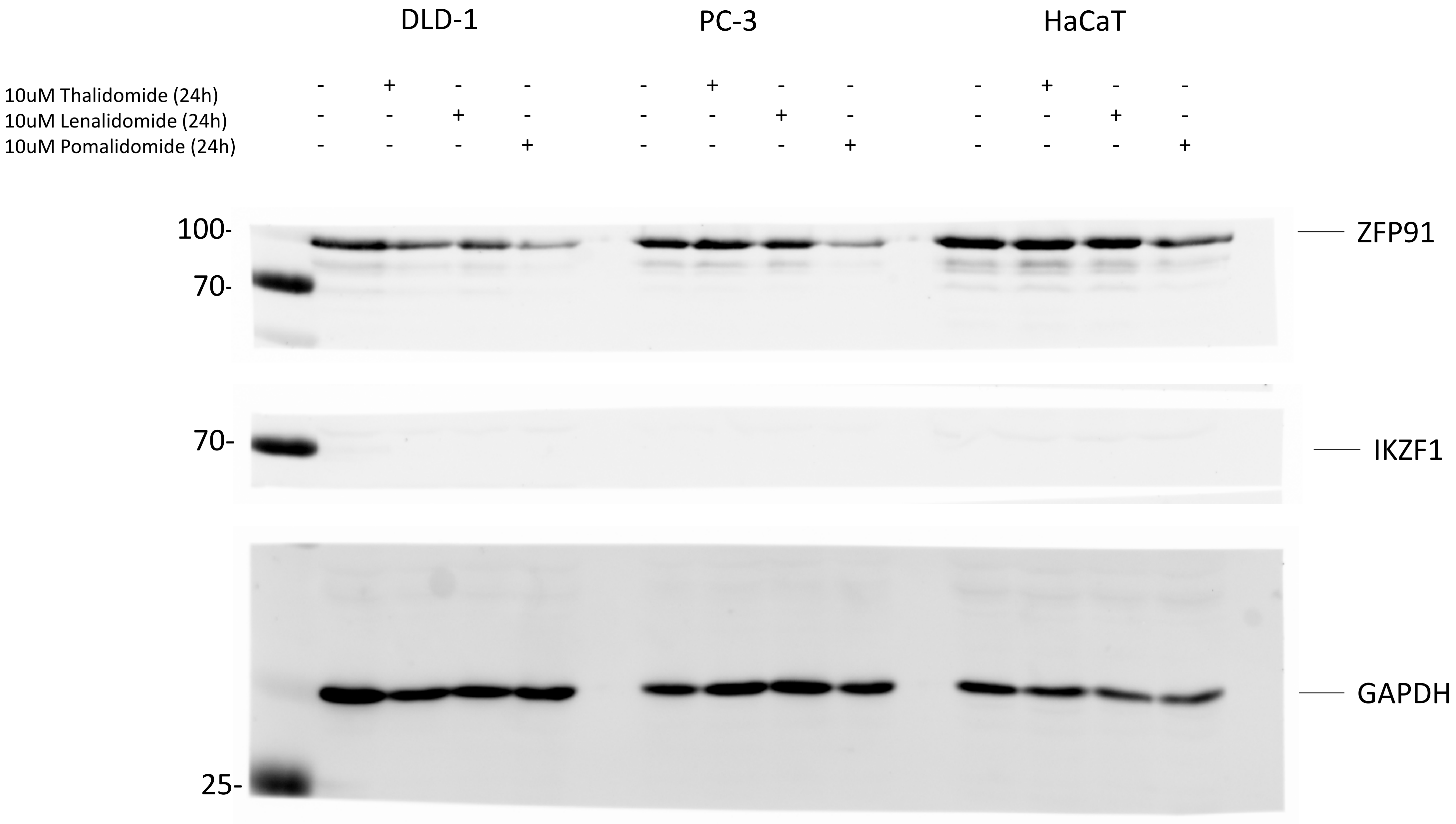
DLD-1
PC-3
HaCaT
10uM Thalidomide (24h) - + - - - + - - - + - -
10uM Lenalidomide (24h) - - + - - - + - - - + -
10uM Pomalidomide (24h) - - - + - - - + - - - +
100-
70-
ZFP91
70-
IKZF1
GAPDH
25-

Figure 1E.

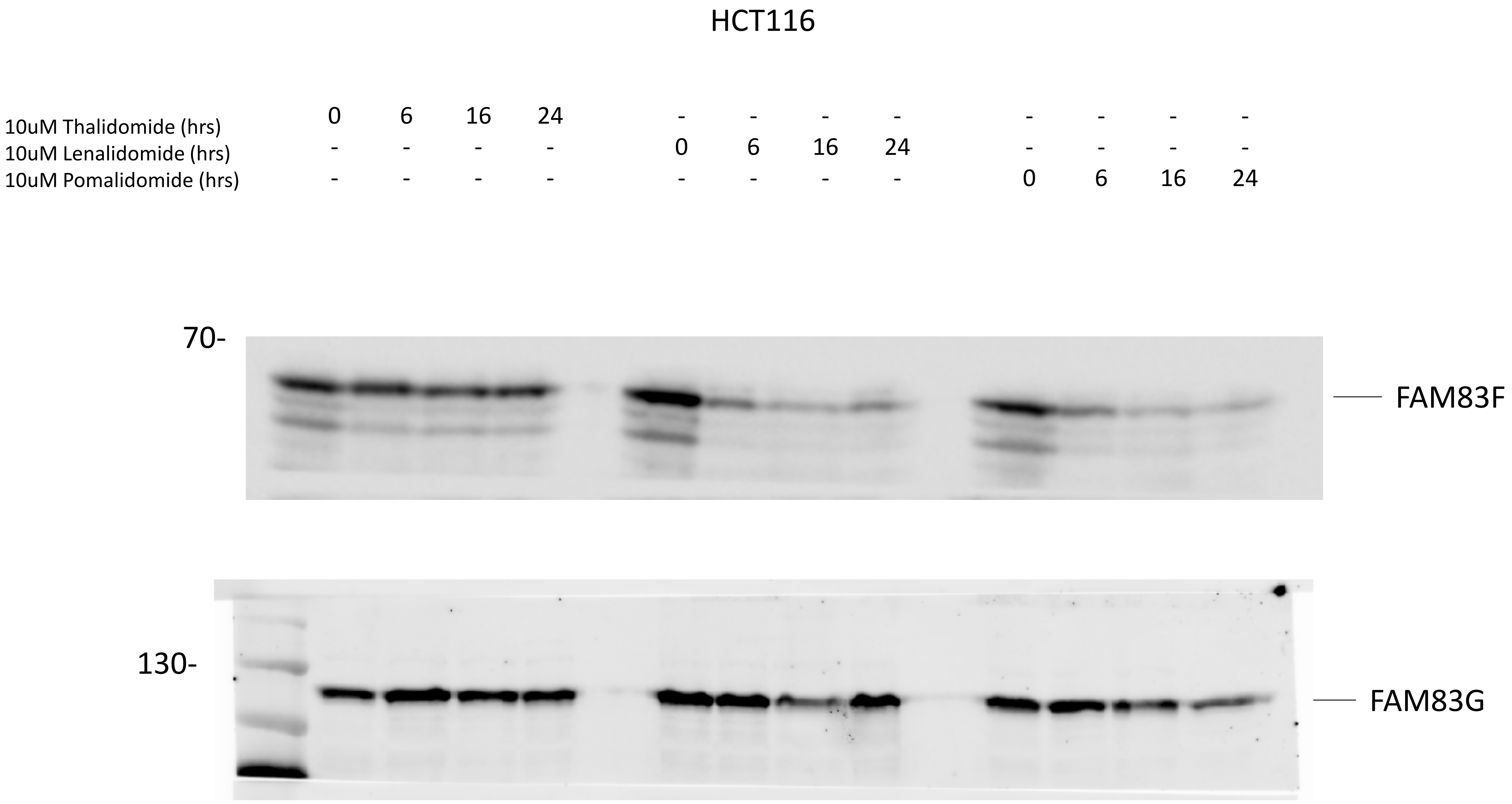
HCT116
10uM Thalidomide (hrs) 0 6 16 24 - - - - - - - -
10uM Lenalidomide (hrs) - - - - 0 6 16 24 - - - -
10uM Pomalidomide (hrs) - - - - - - - - 0 6 16 24
70-
FAM83F
130-
FAM83G

Figure 1E.

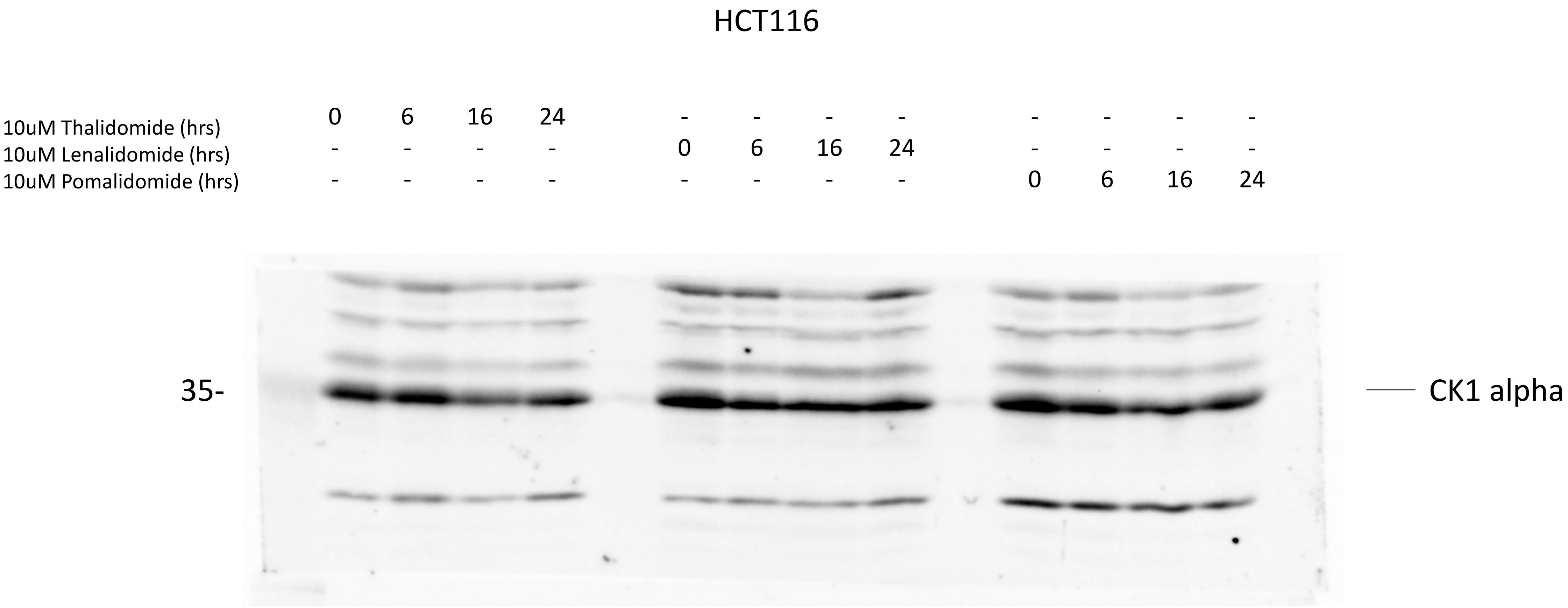
HCT116
10uM Thalidomide (hrs) 0 6 16 24 - - - - - - - -
10uM Lenalidomide (hrs) - - - - 0 6 16 24 - - - -
10uM Pomalidomide (hrs) - - - - - - - - 0 6 16 24
35-
CK1 alpha

Figure 1E.

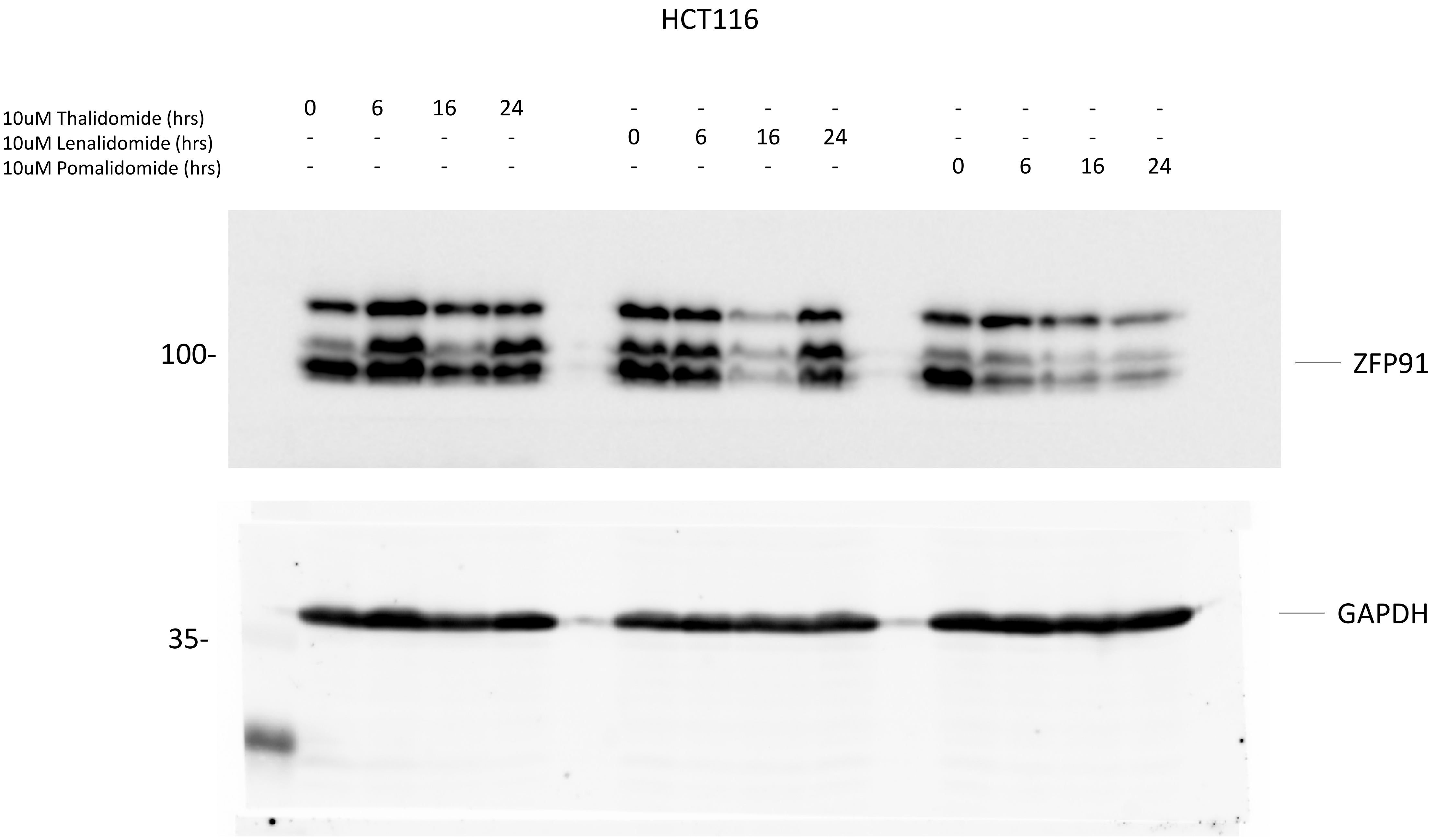
HCT116
10uM Thalidomide (hrs)
10uM Lenalidomide (hrs)
10uM Pomalidomide (hrs)
0 6 16 24 - - - - - - - -
- - - - 0 6 16 24 - - - -
- - - - - - - - 0 6 16 24
100-
ZFP91
35-
GAPDH

Figure 1F.

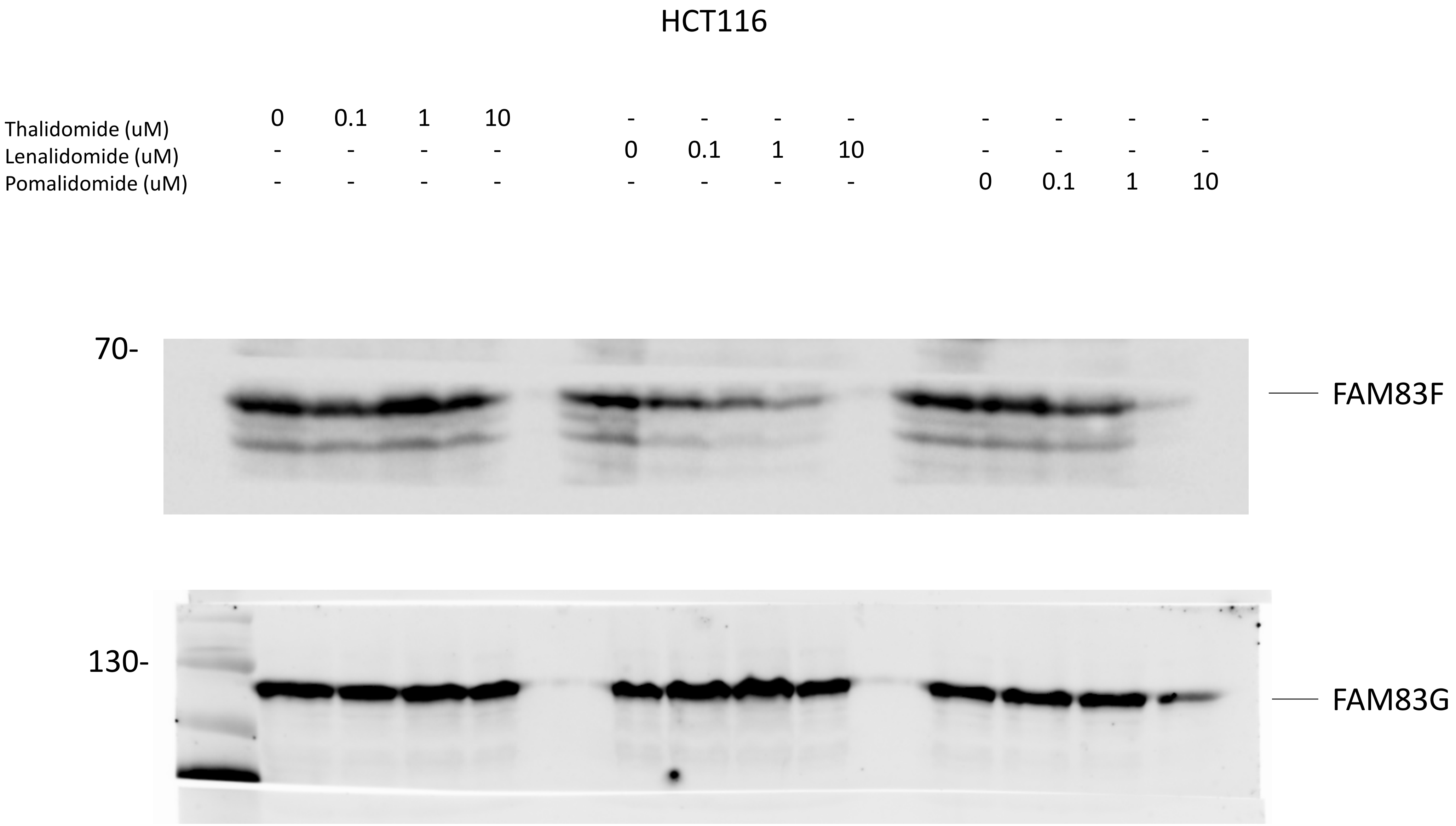
HCT116
Thalidomide (uM) 0 0.1 1 10 - - - - - - - -
Lenalidomide (uM) - - - - 0 0.1 1 10 - - - -
Pomalidomide (uM) - - - - - - - - 0 0.1 1 10
70-
FAM83F
130-
FAM83G

Figure 1F.

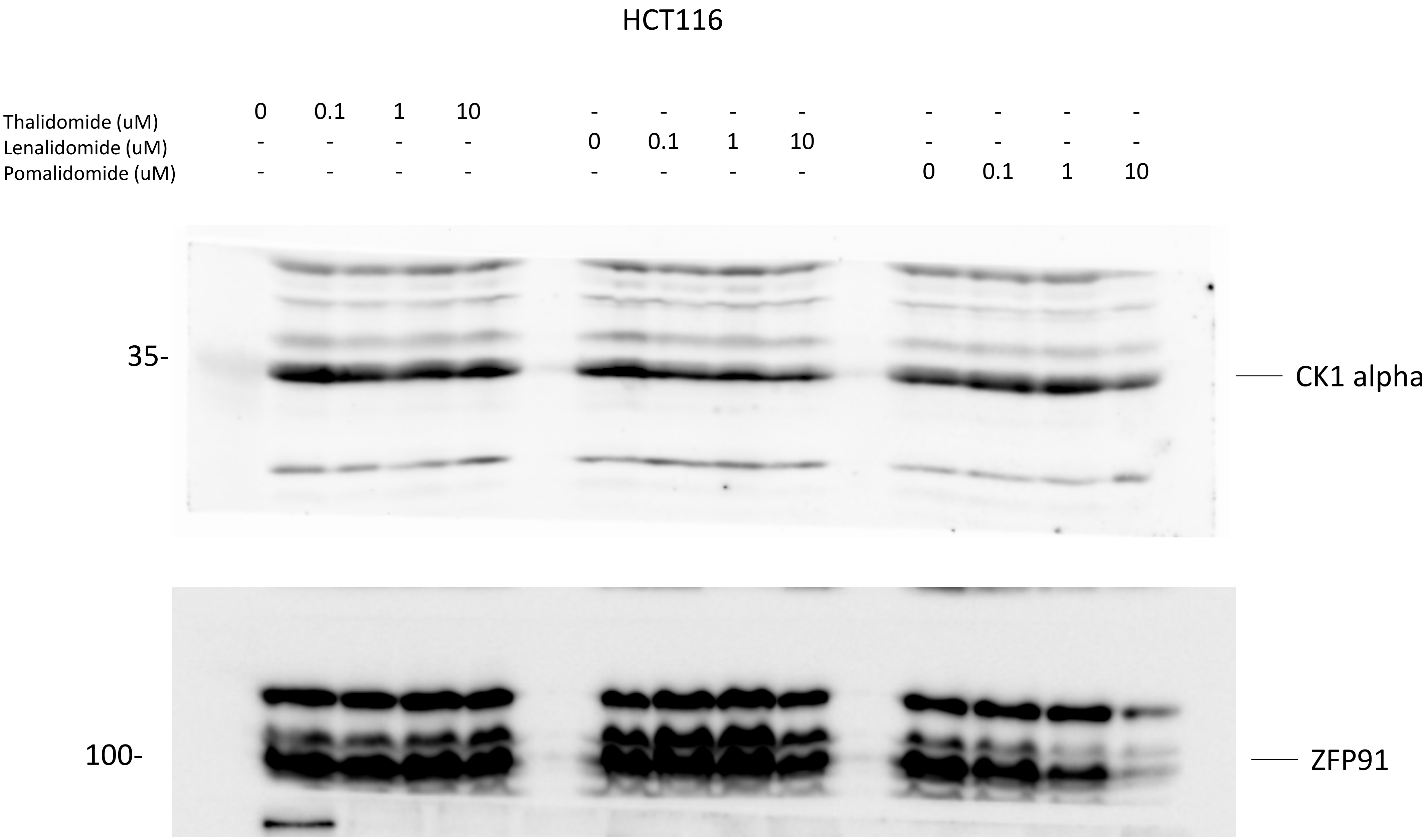
HCT116
Thalidomide (uM) 0 0.1 1 10 - - - - - - - -
Lenalidomide (uM) - - - - 0 0.1 1 10 - - - -
Pomalidomide (uM) - - - - - - - - 0 0.1 1 10
35-
CK1 alpha
100-
ZFP91

Figure 1F.

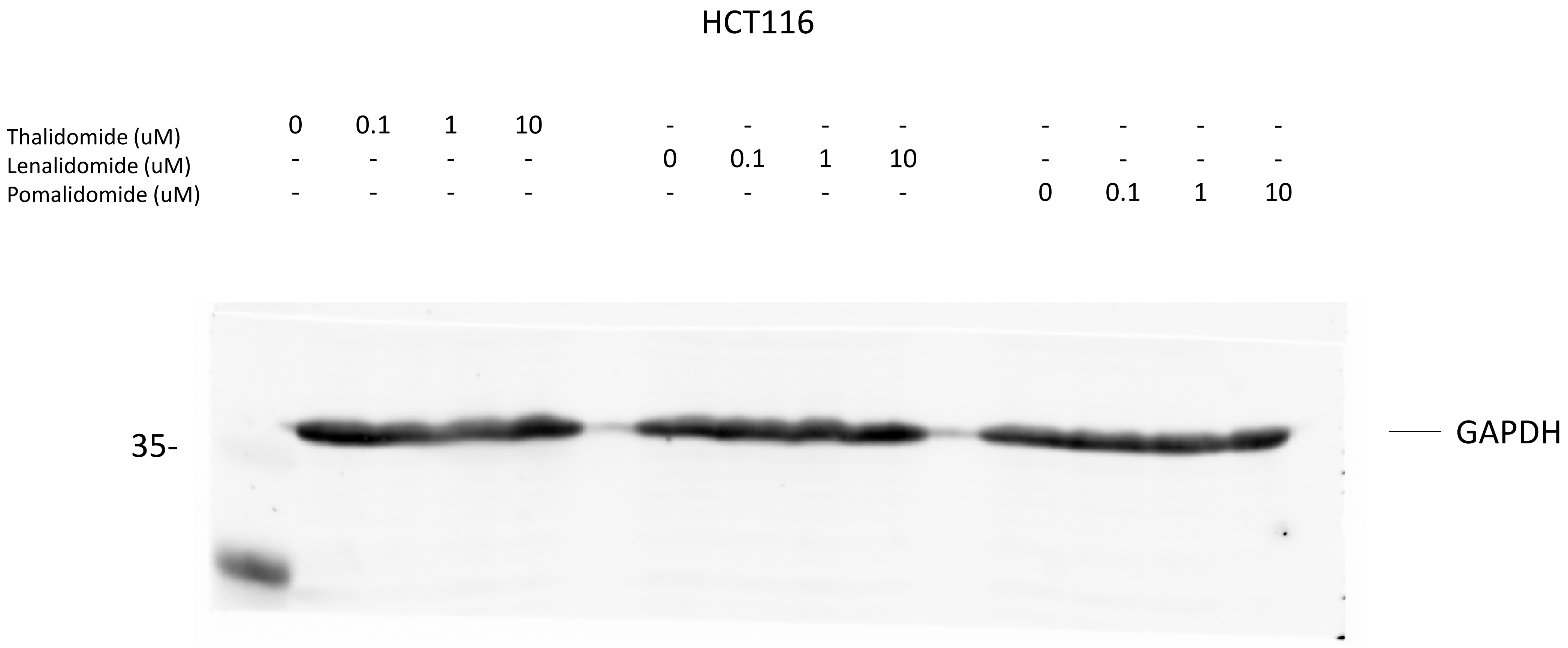
HCT116
Thalidomide (uM) 0 0.1 1 10 - - - - - - - -
Lenalidomide (uM) - - - - 0 0.1 1 10 - - - -
Pomalidomide (uM) - - - - - - - - 0 0.1 1 10
35-
GAPDH
