# Supplementary material for: IMiDs induce FAM83F degradation via an interaction with CK1α to attenuate Wnt signalling
Source: Life Sci Alliance. 2020 Dec 23;4(2):e202000804. doi: 10.26508/lsa.202000804 (PMC7768194; doi:10.26508/lsa.202000804)

Sup. Figure 1A.

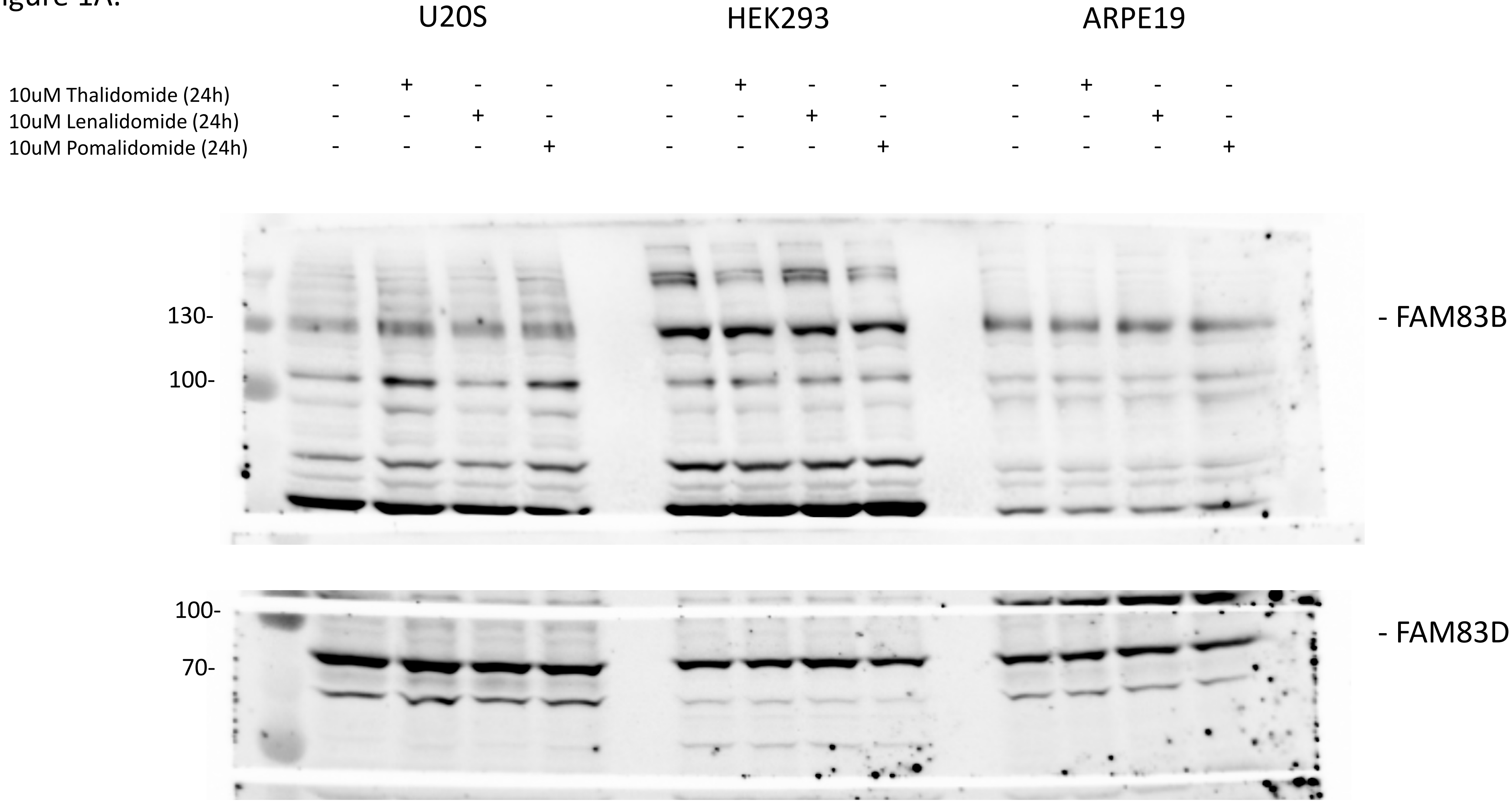

Sup. Figure 1A.

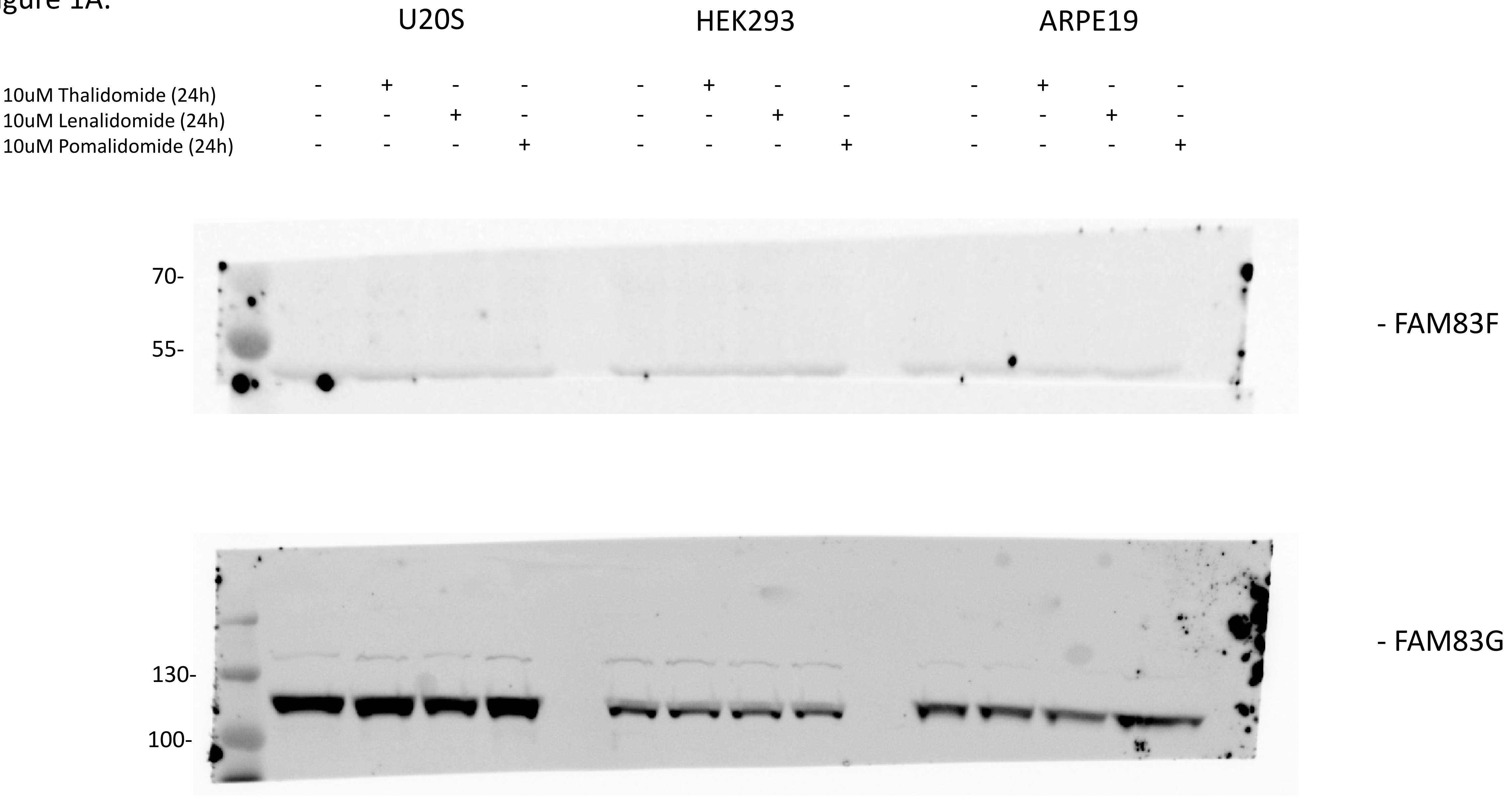

Sup. Figure 1A.

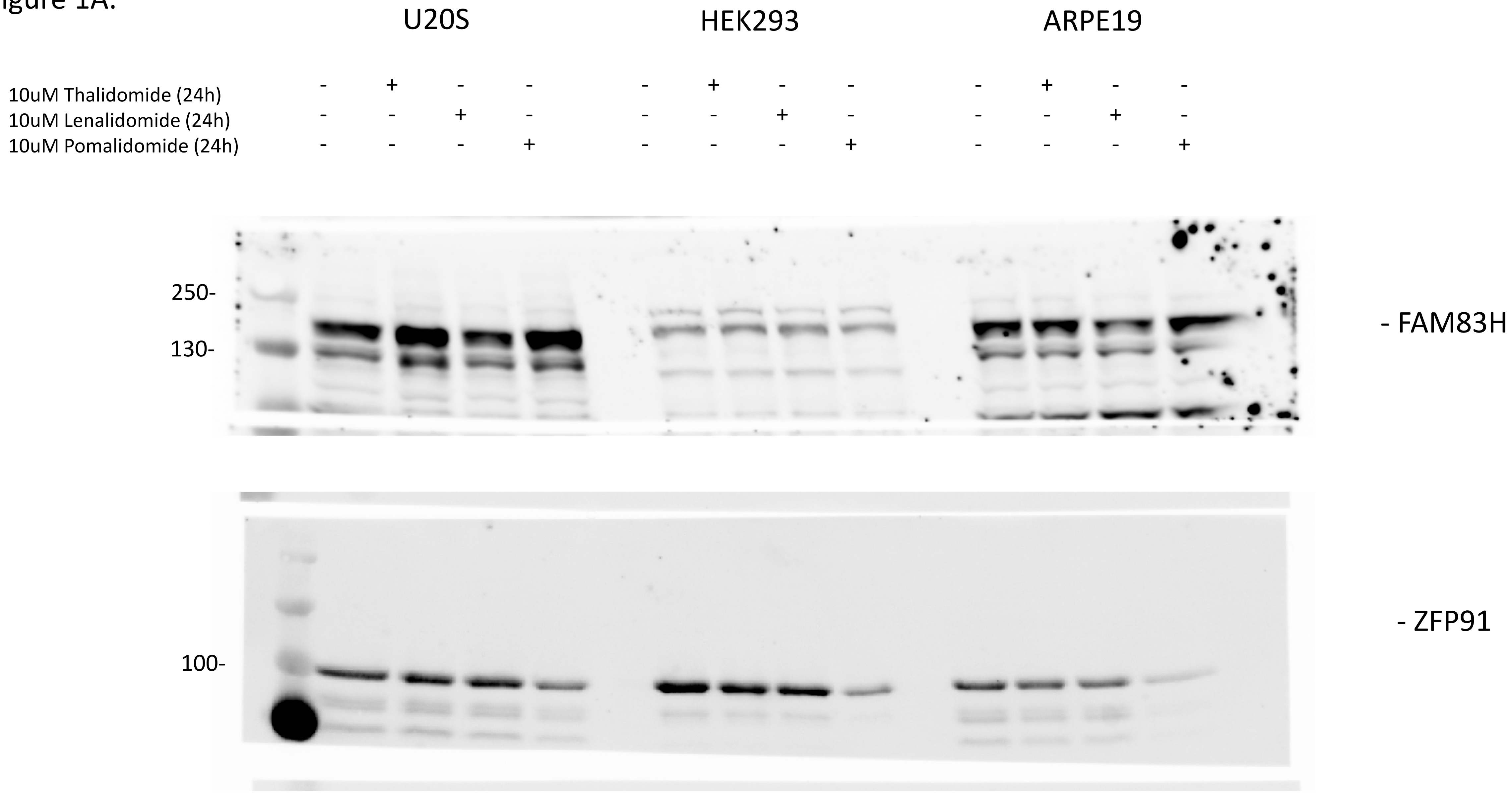

Sup. Figure 1A.

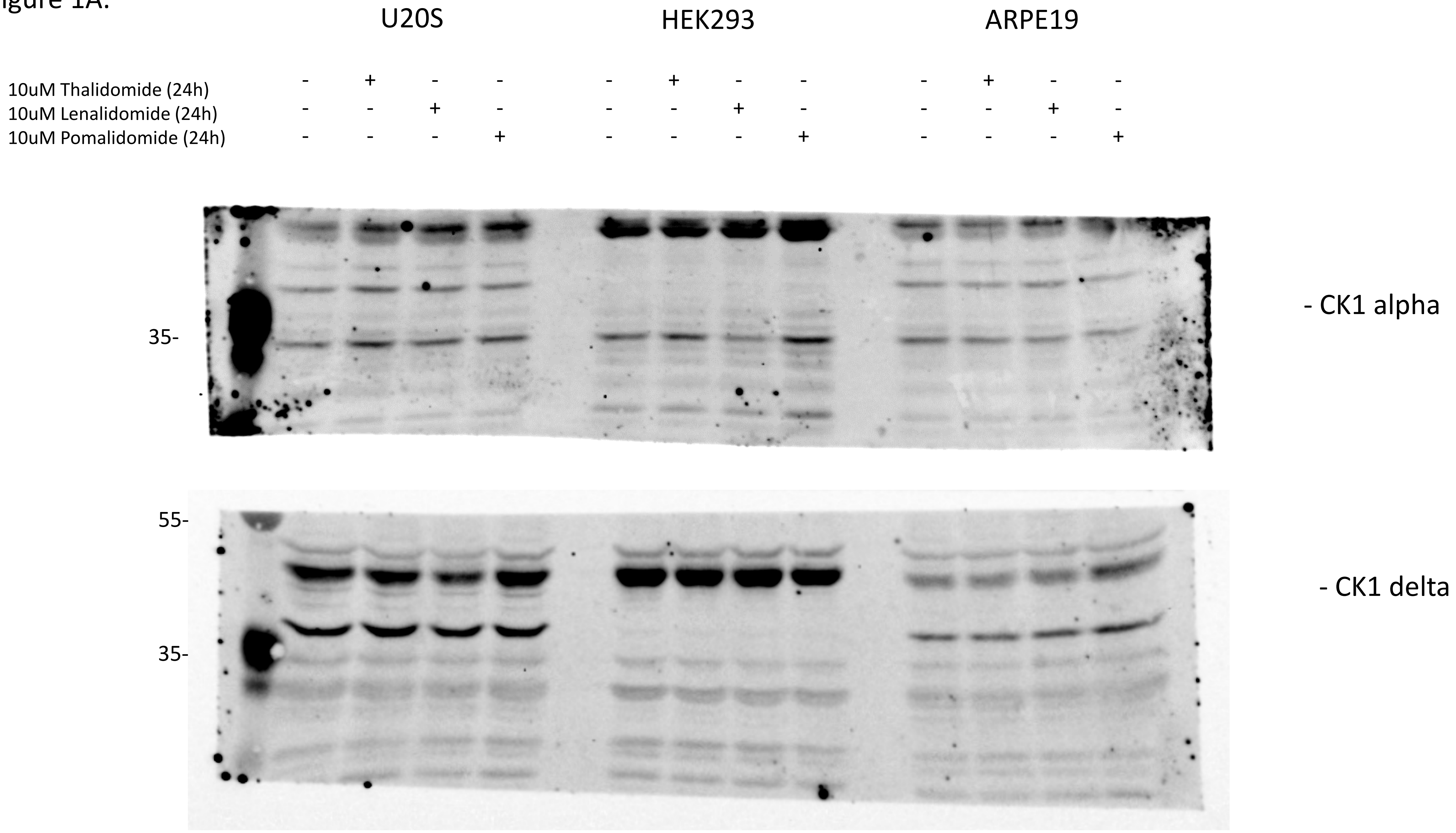

Sup. Figure 1A.

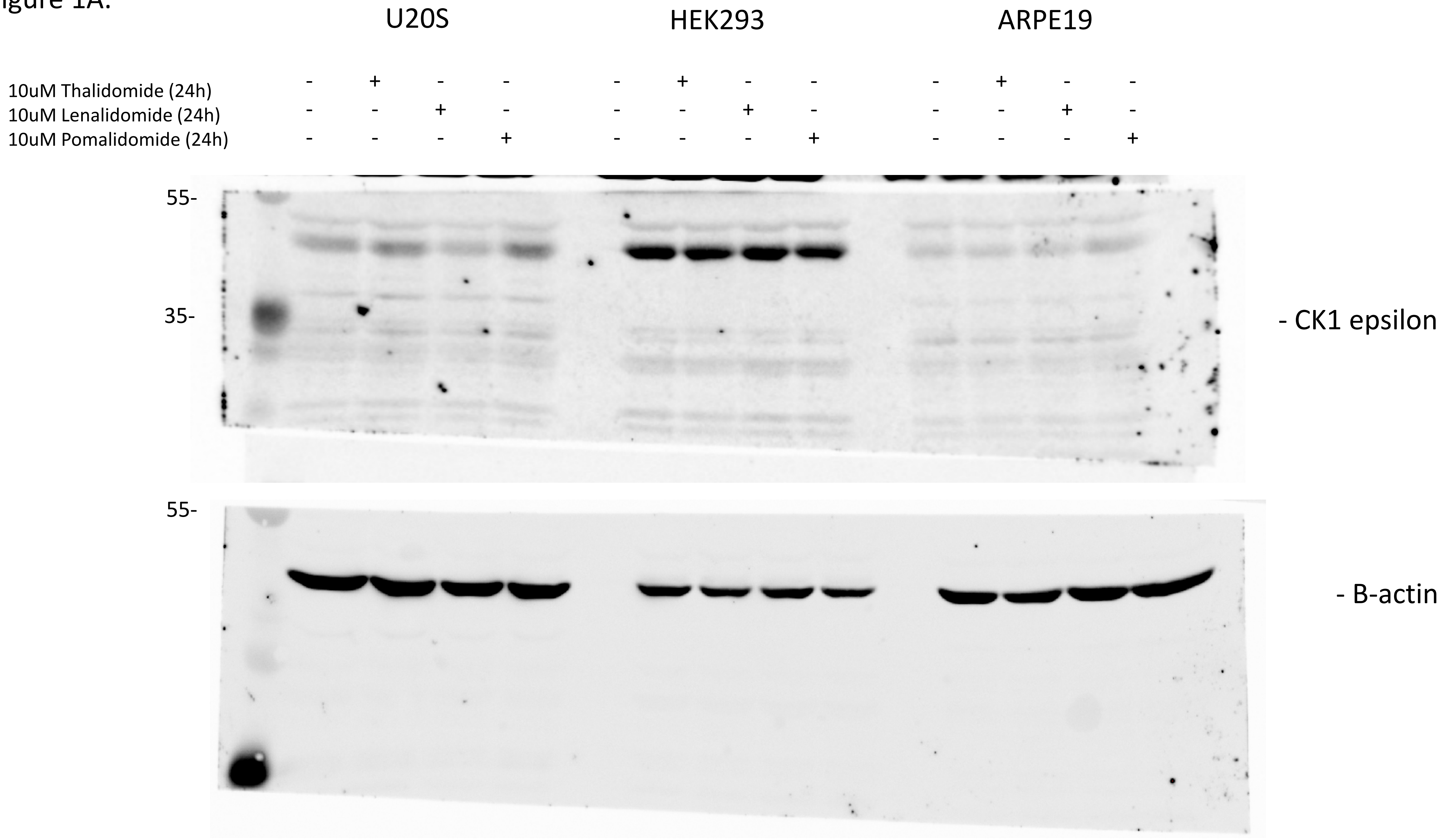

Sup. Figure 1B.

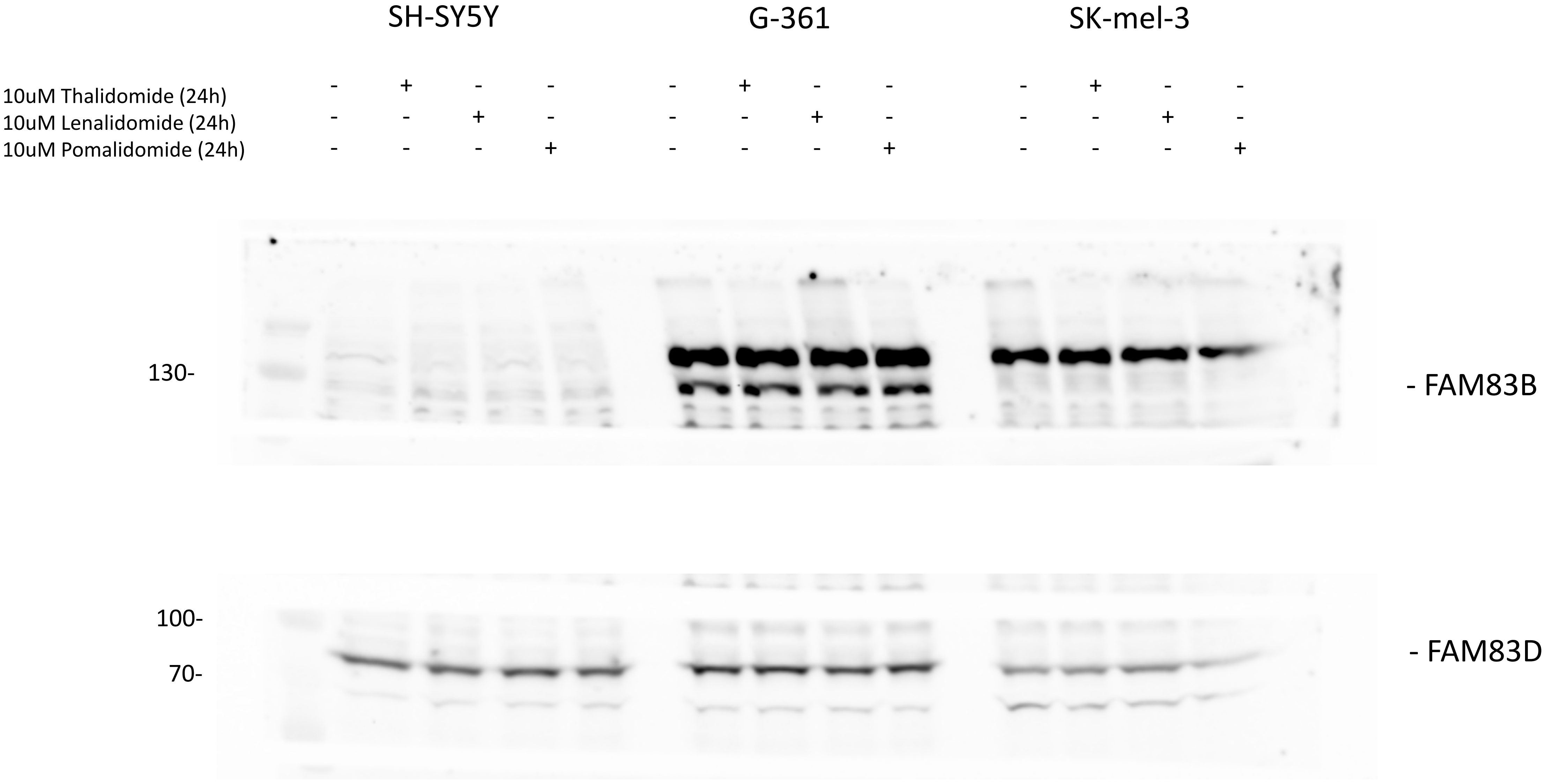

Sup. Figure 1B.

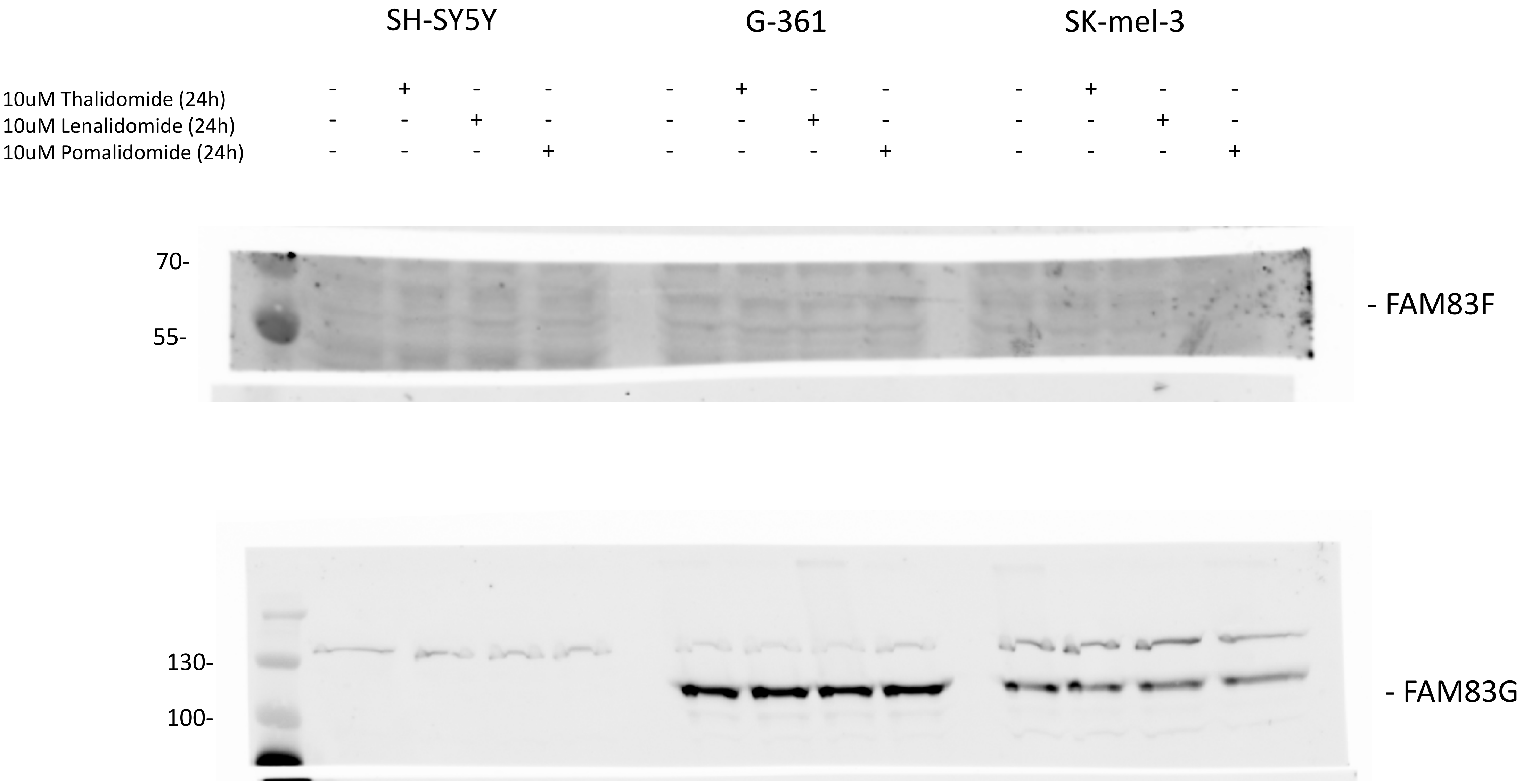

Sup. Figure 1B.

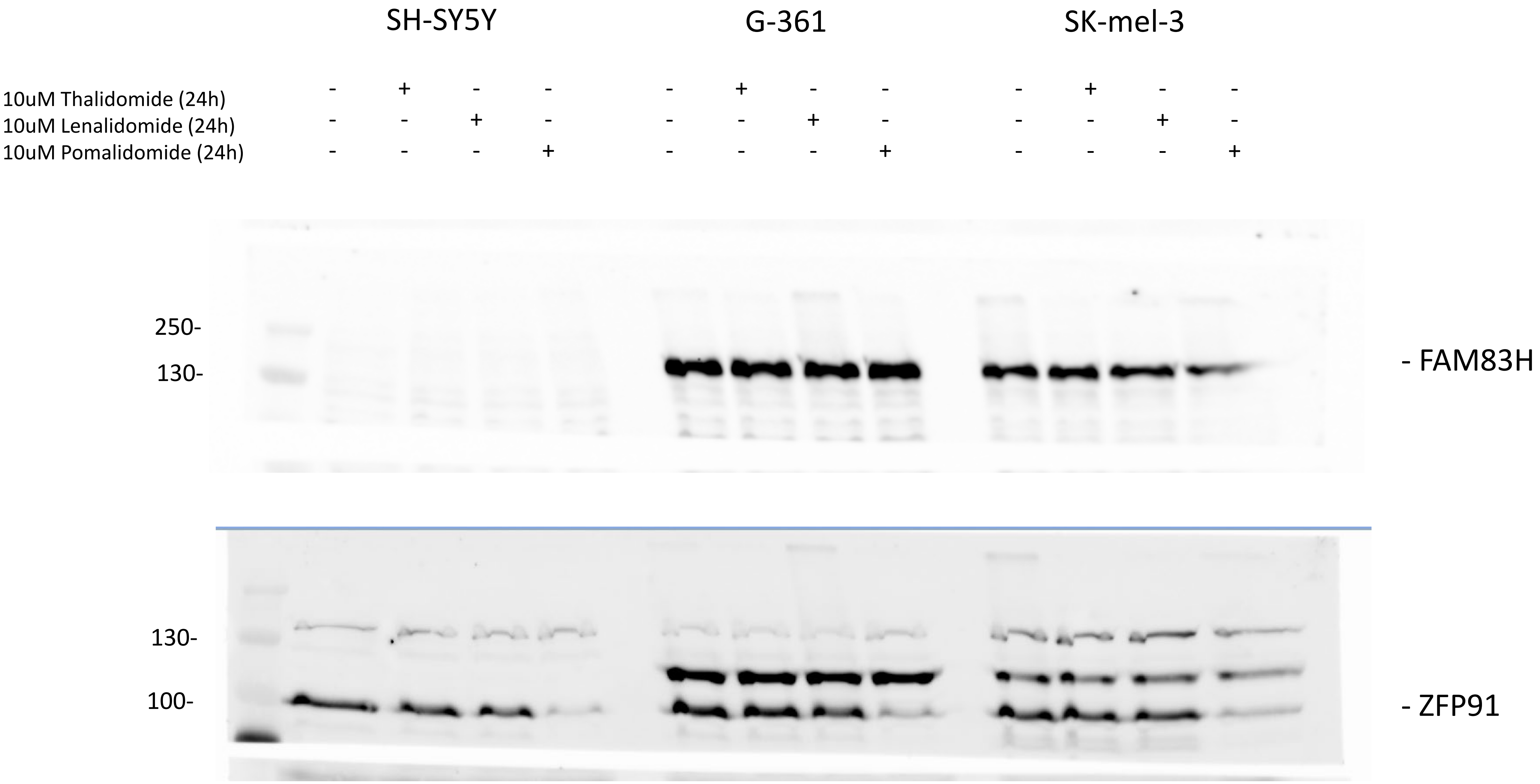

Sup. Figure 1B.

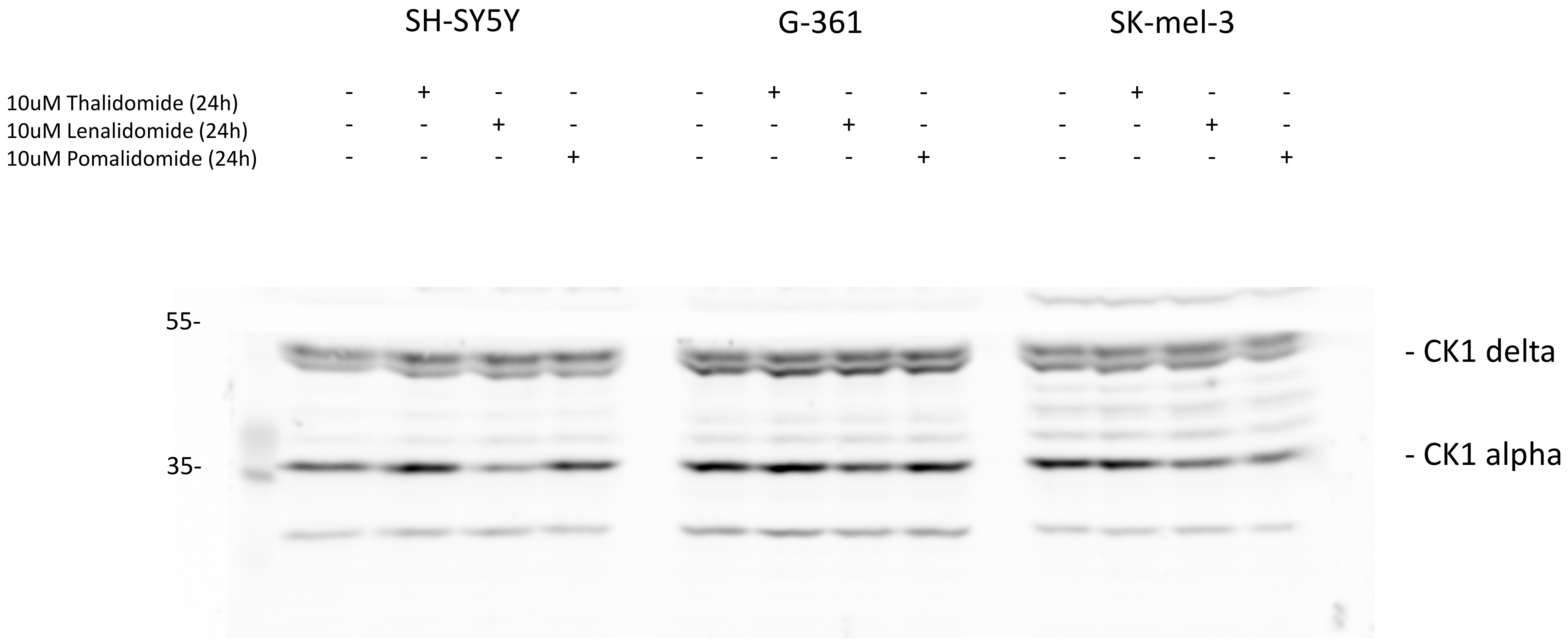

Sup. Figure 1B.

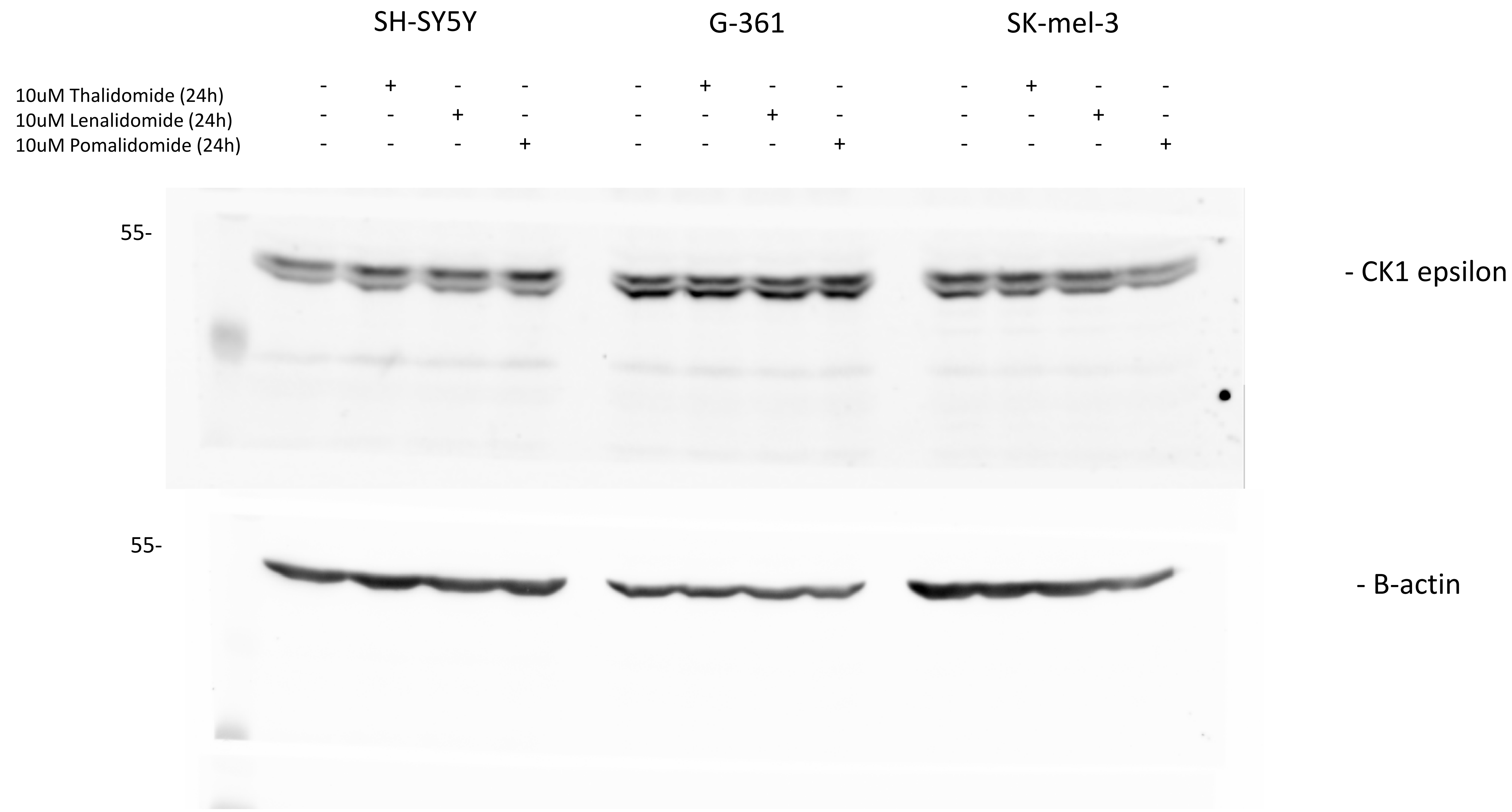

Supplement: Supplementary file 2 [file LSA-2020-00804_SdataFS1.pdf]
