# Supplementary material for: IMiDs induce FAM83F degradation via an interaction with CK1α to attenuate Wnt signalling
Source: Life Sci Alliance. 2020 Dec 23;4(2):e202000804. doi: 10.26508/lsa.202000804 (PMC7768194; doi:10.26508/lsa.202000804)

Sup. Figure 2A.

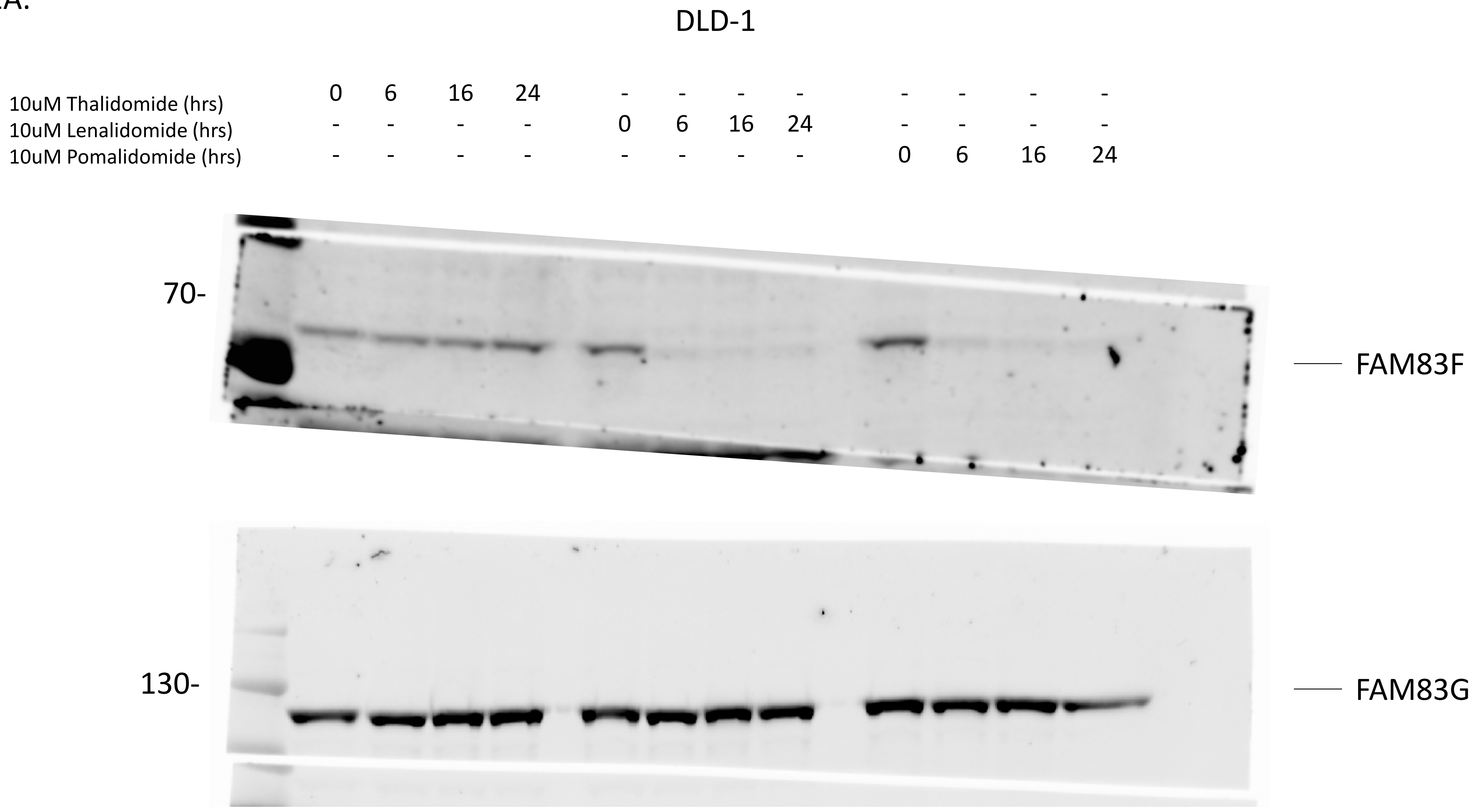

Sup. Figure 2A.

DLD-1

10uM Thalidomide (hrs)	0	6	16	24	-	-	-	-	-	-	-	-
10uM Lenalidomide (hrs)	-	-	-	-	0	6	16	24	-	-	-	-
10uM Pomalidomide (hrs)	-	-	-	-	-	-	-	-	0	6	16	24

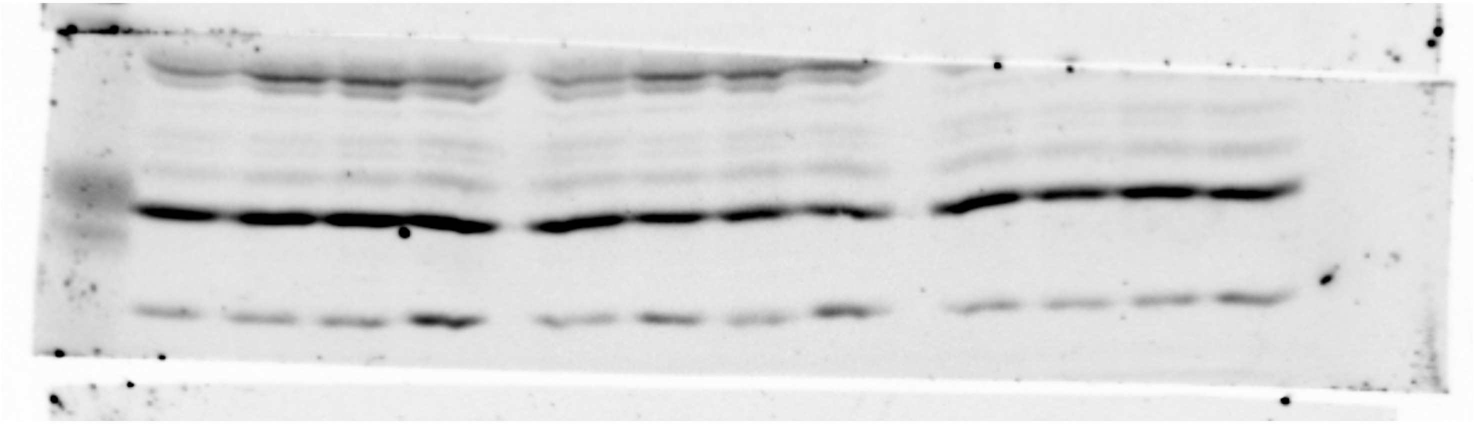

Sup. Figure 2A.

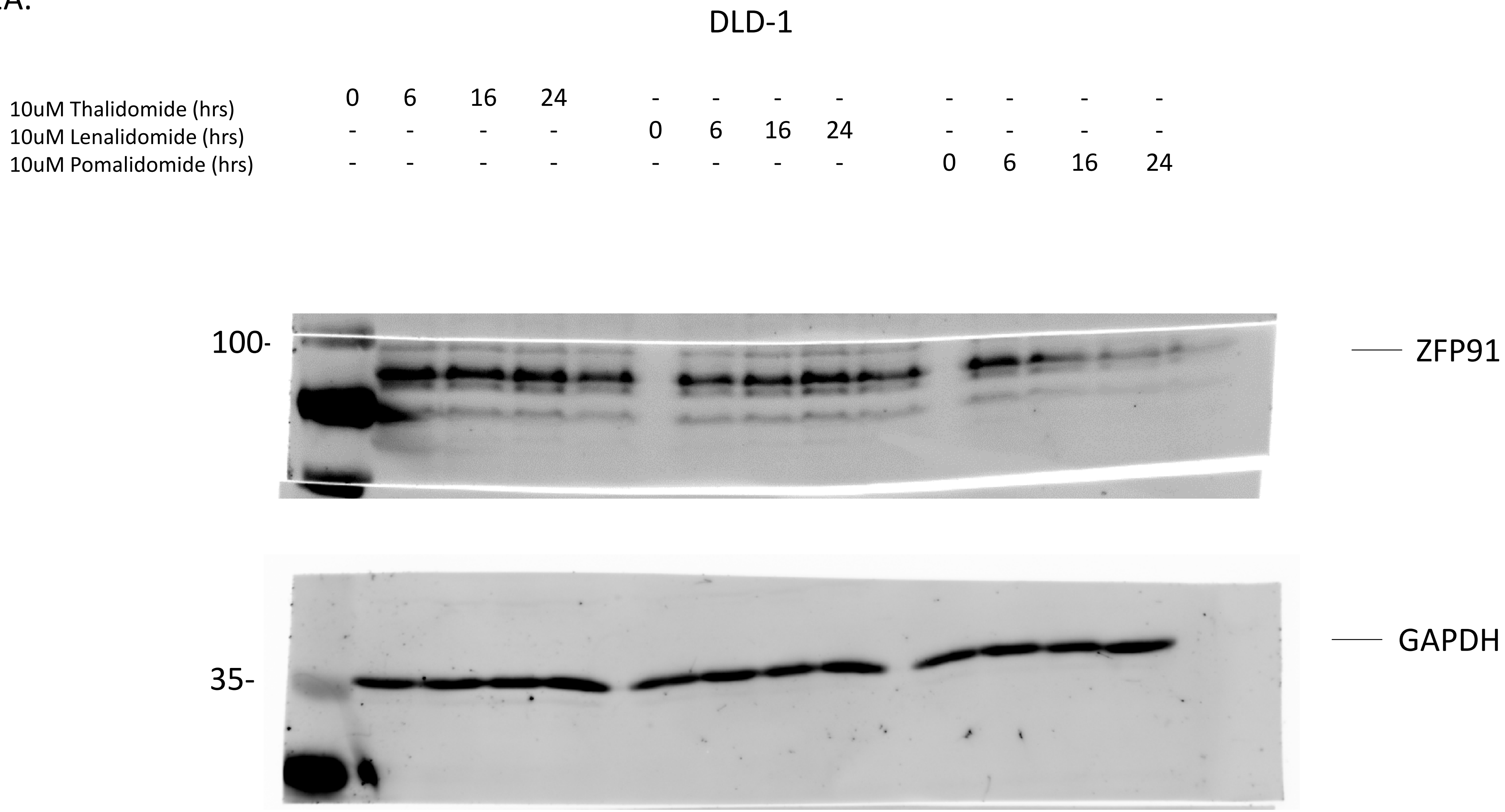

Sup. Figure 2B.

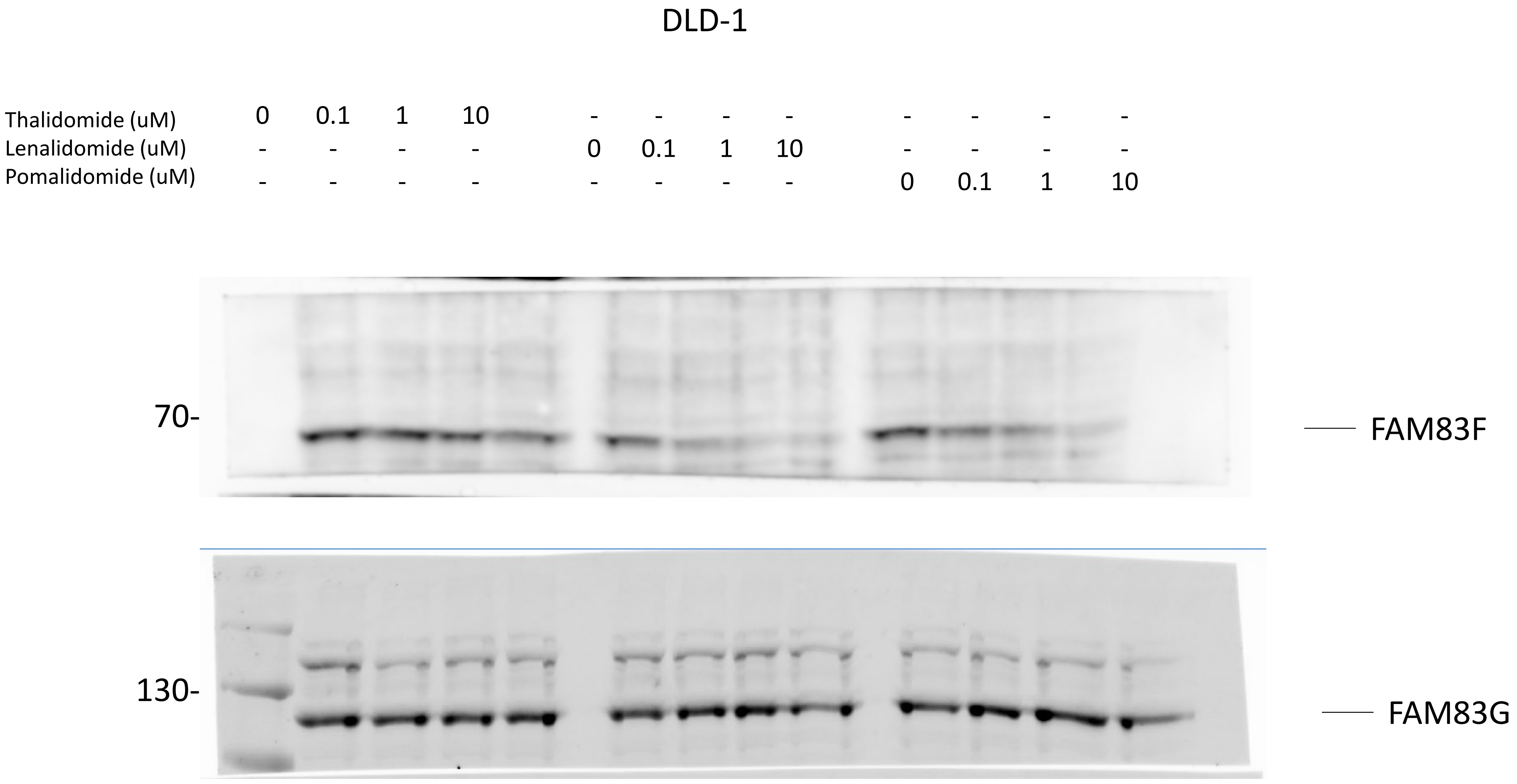

Sup. Figure 2B.

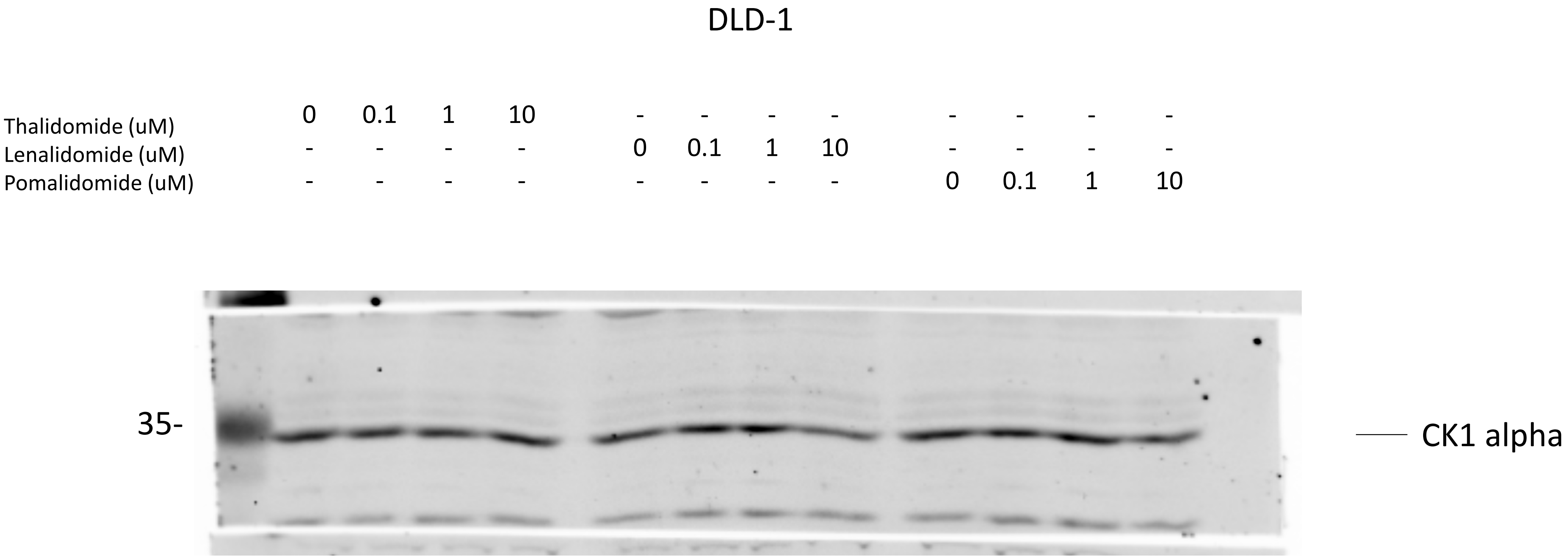

Sup. Figure 2B.

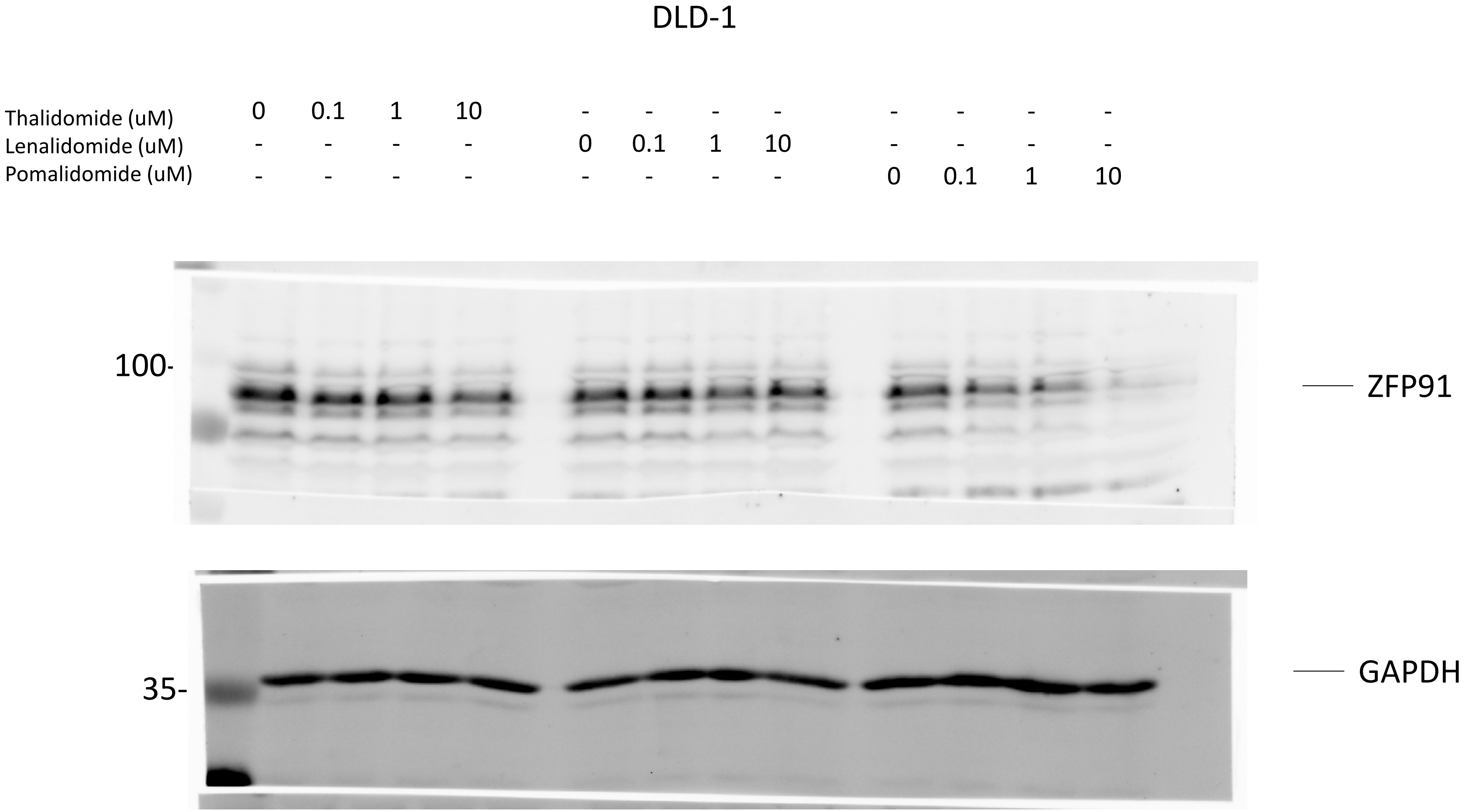

Sup. Figure 2C.

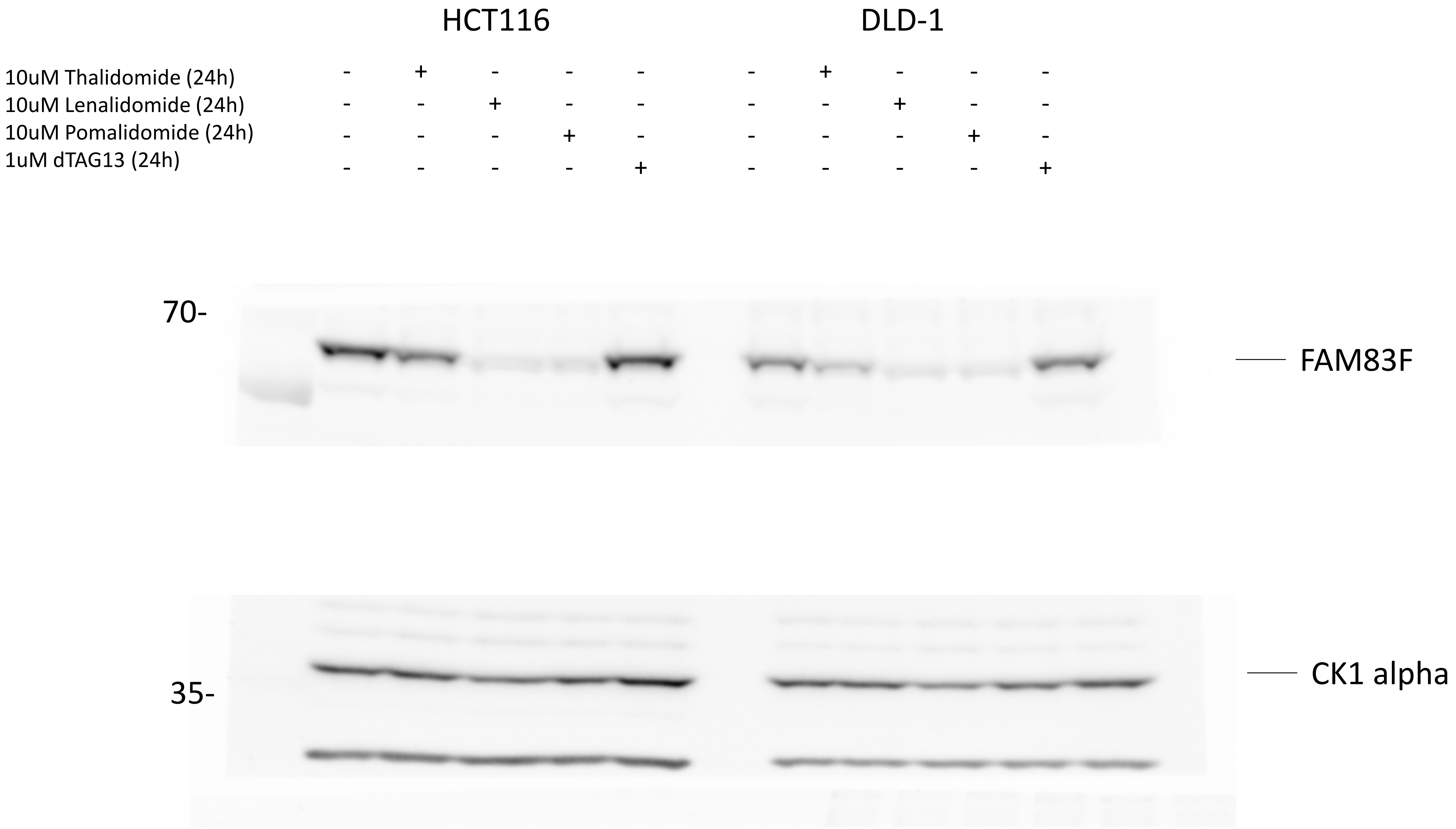

Sup. Figure 2C.

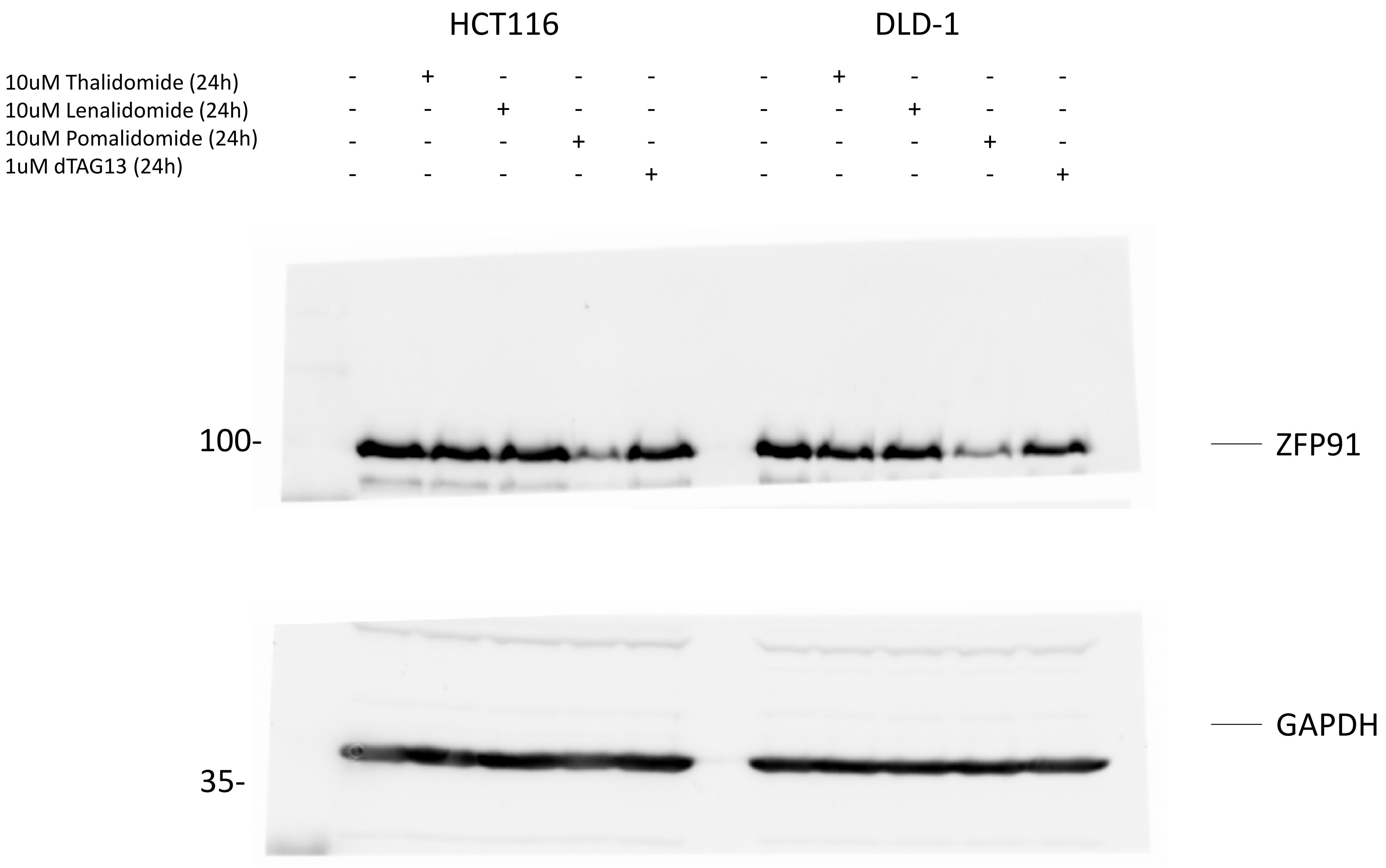

Supplement: Supplementary file 3 [file LSA-2020-00804_SdataFS2.pdf]
