# Supplementary figures and images for: IMiDs induce FAM83F degradation via an interaction with CK1α to attenuate Wnt signalling
Source: Life Sci Alliance. 2020 Dec 23;4(2):e202000804. doi: 10.26508/lsa.202000804 (PMC7768194; doi:10.26508/lsa.202000804)

Figure 2B.

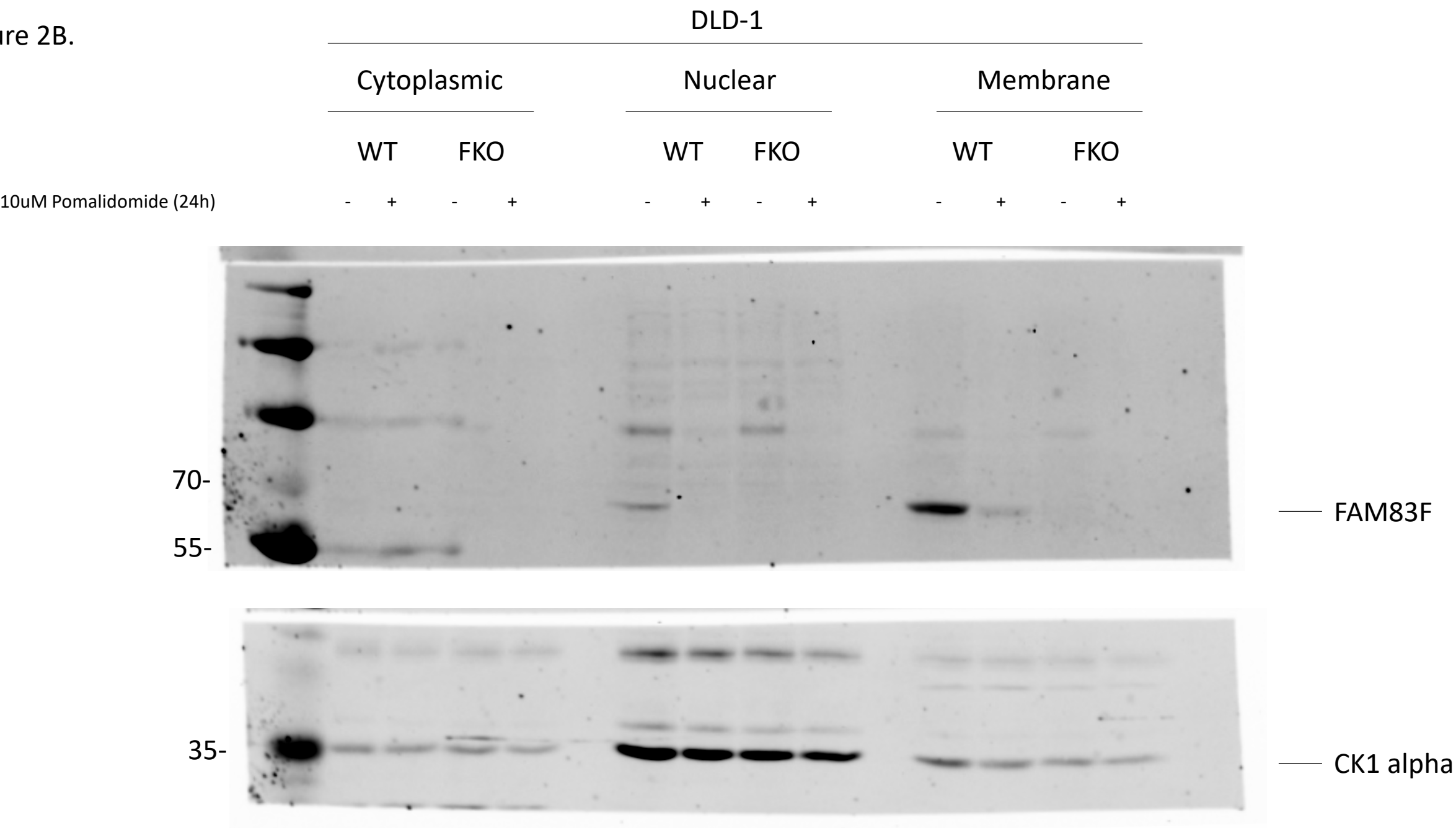

Figure 2B.

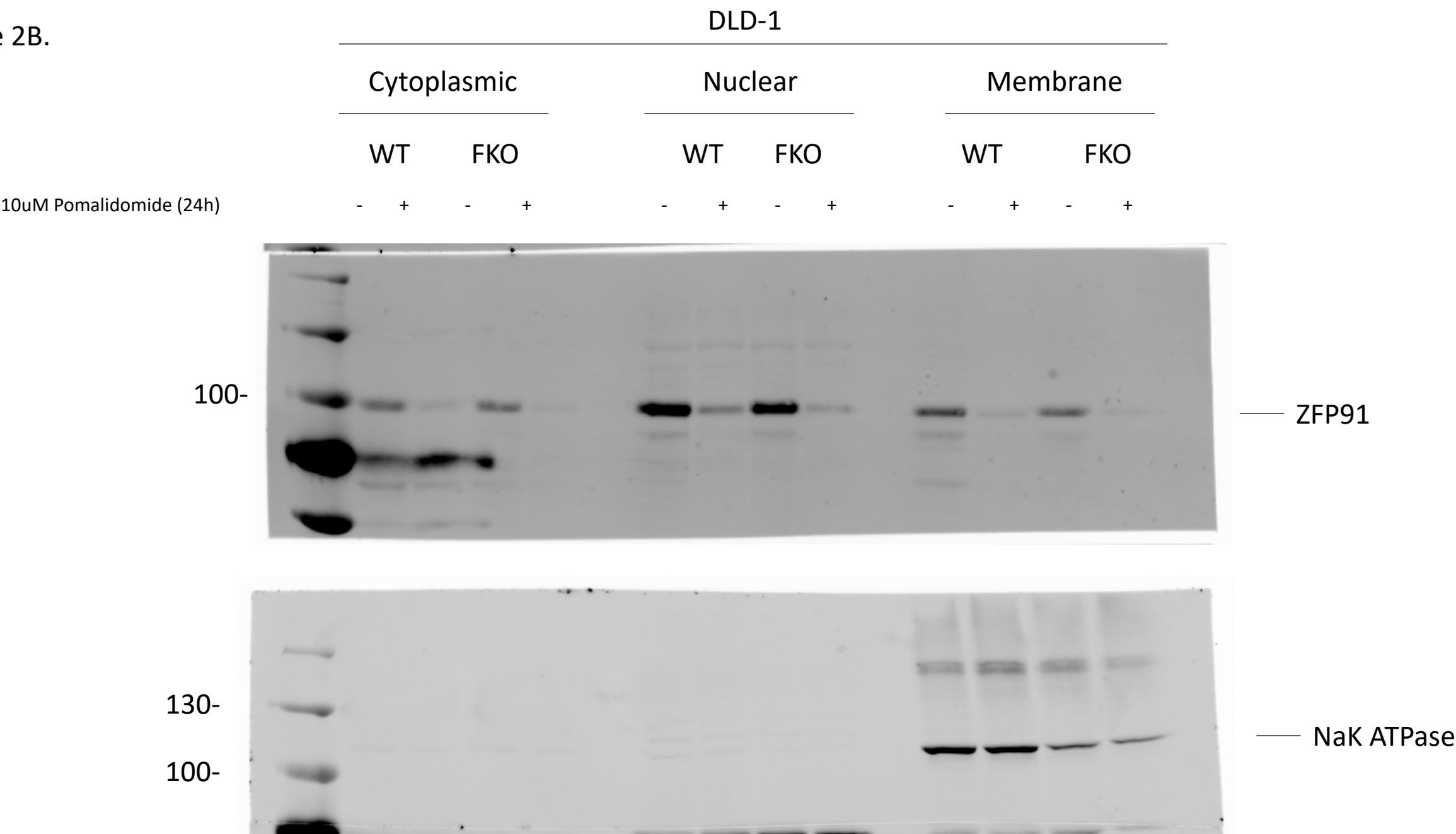

Figure 2B.

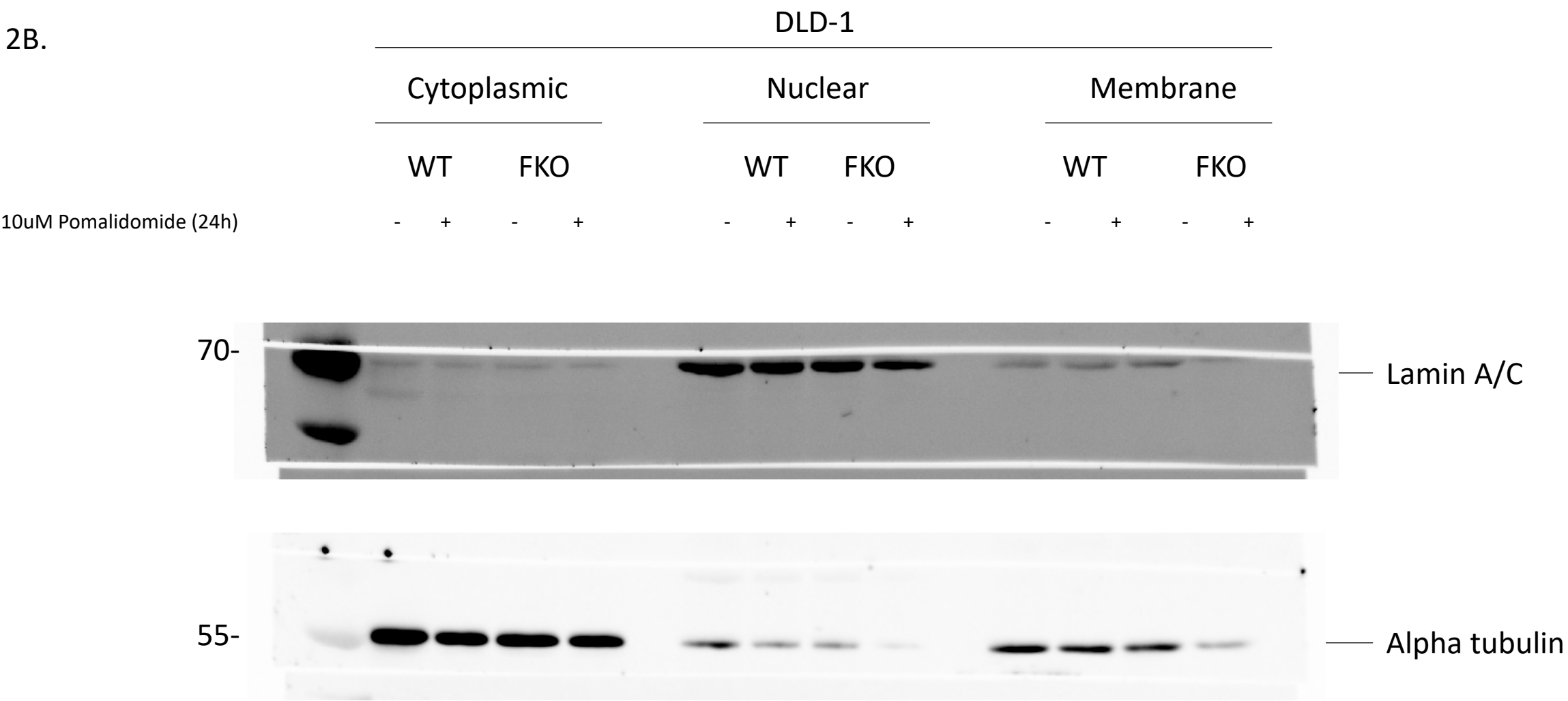

Supplement: Supplementary file 4 [file LSA-2020-00804_SdataF2.pdf]

Sup. Figure 3A.

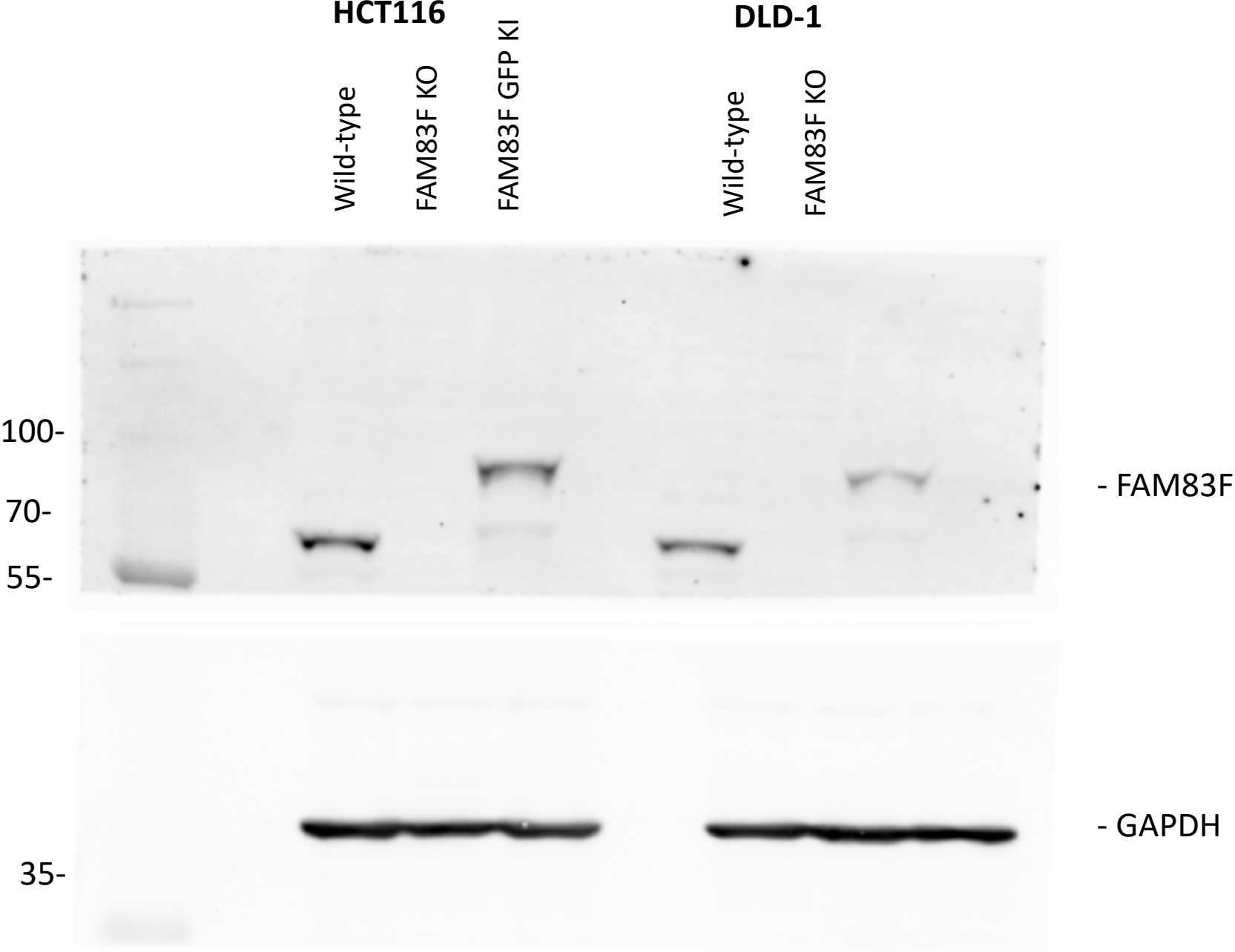

Sup. Figure 3B.

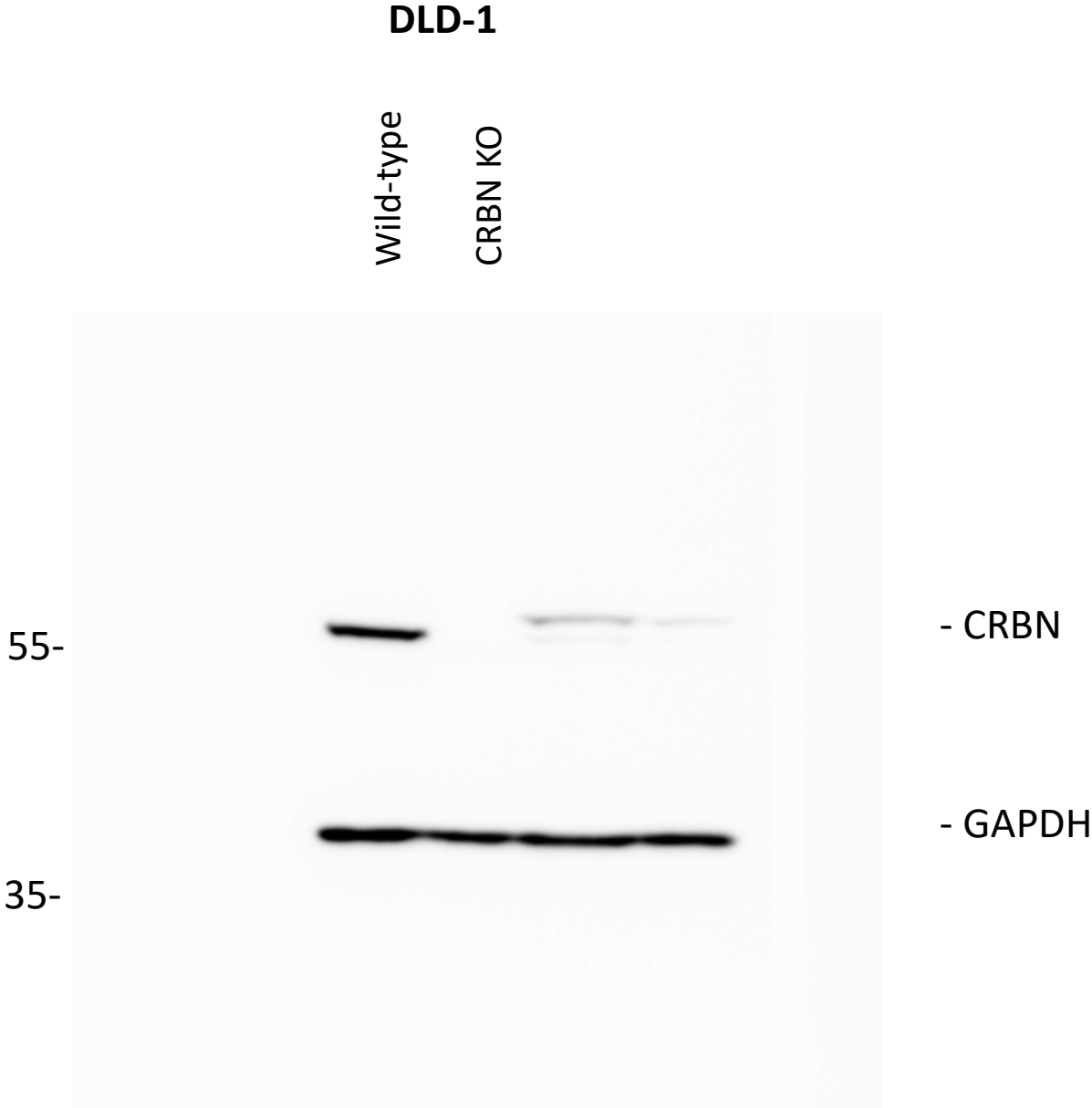

Sup. Figure 3C.

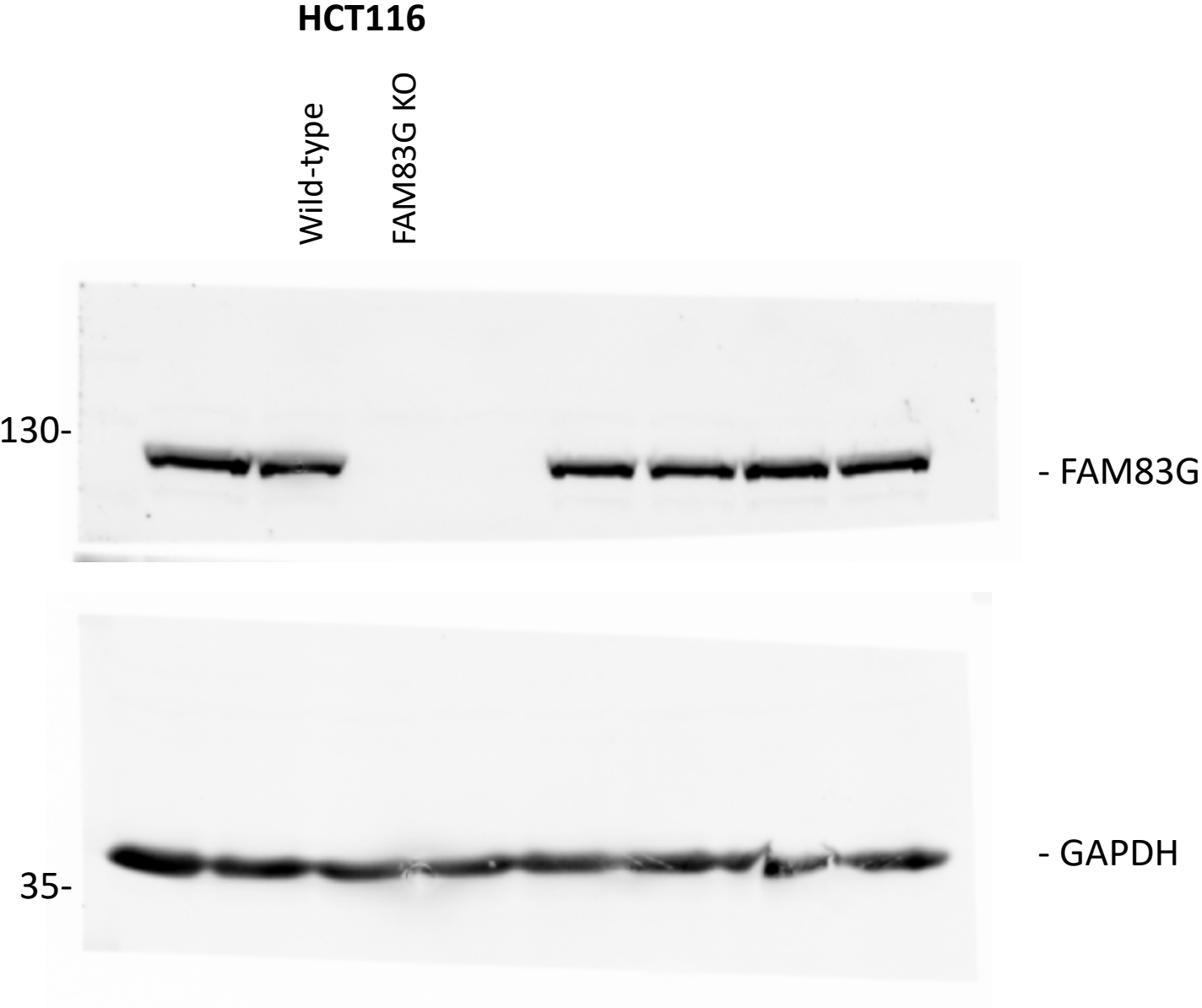

Supplement: Supplementary file 5 [file LSA-2020-00804_SdataFS3.pdf]

Sup. Figure 7.

MV4.11

|                         |   |   |   |   |   |   |
|-------------------------|---|---|---|---|---|---|
| 10uM Thalidomide (24h)  | - | + | - | - | - | - |
| 10uM Lenalidomide (24h) | - | - | + | - | - | - |
| 10uM Pomalidomide (24h) | - | - | - | + | - | - |
| 10uM Iberdomide (24h)   | - | - | - | - | + | - |
| 10uM BTX161 (24h)       | - | - | - | - | - | + |

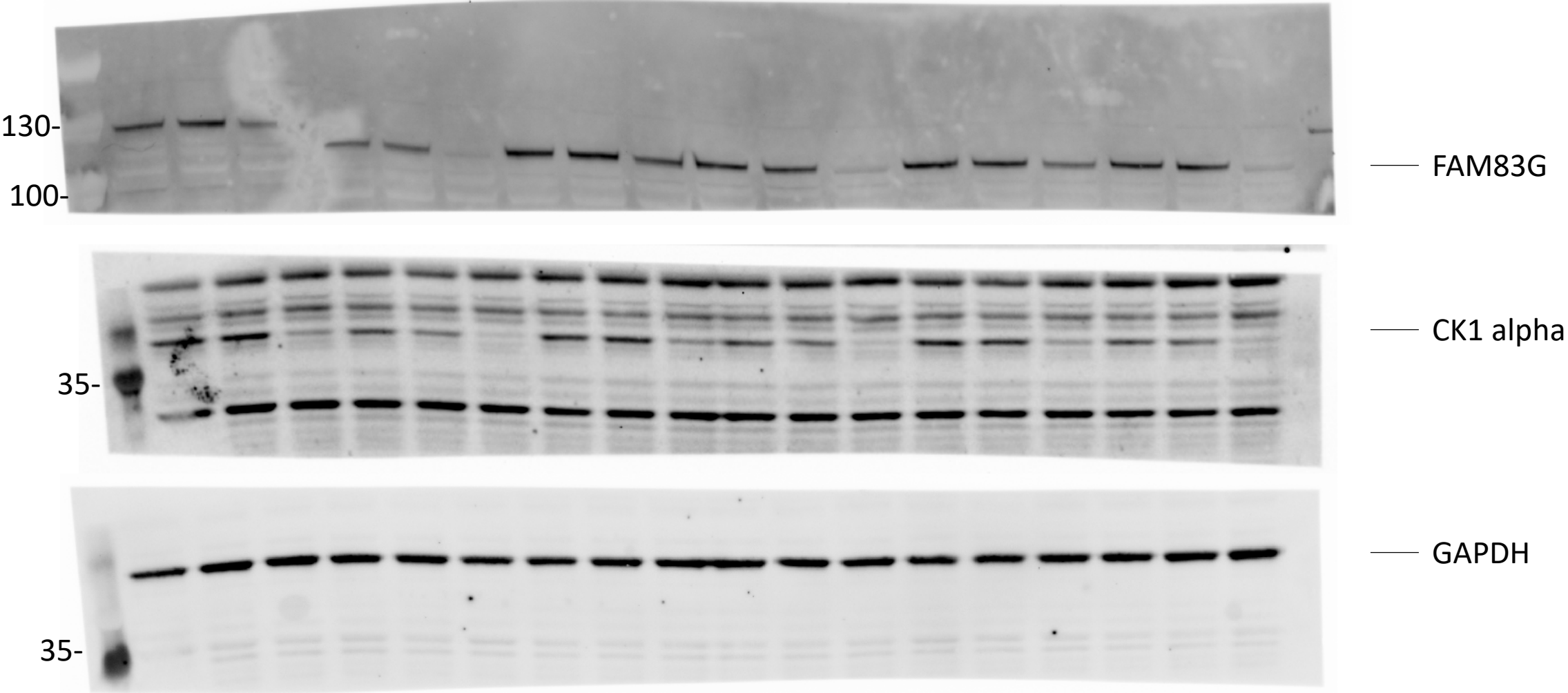

Supplement: Supplementary file 11 [file LSA-2020-00804_SdataFS7.pdf]

Figure 7B.

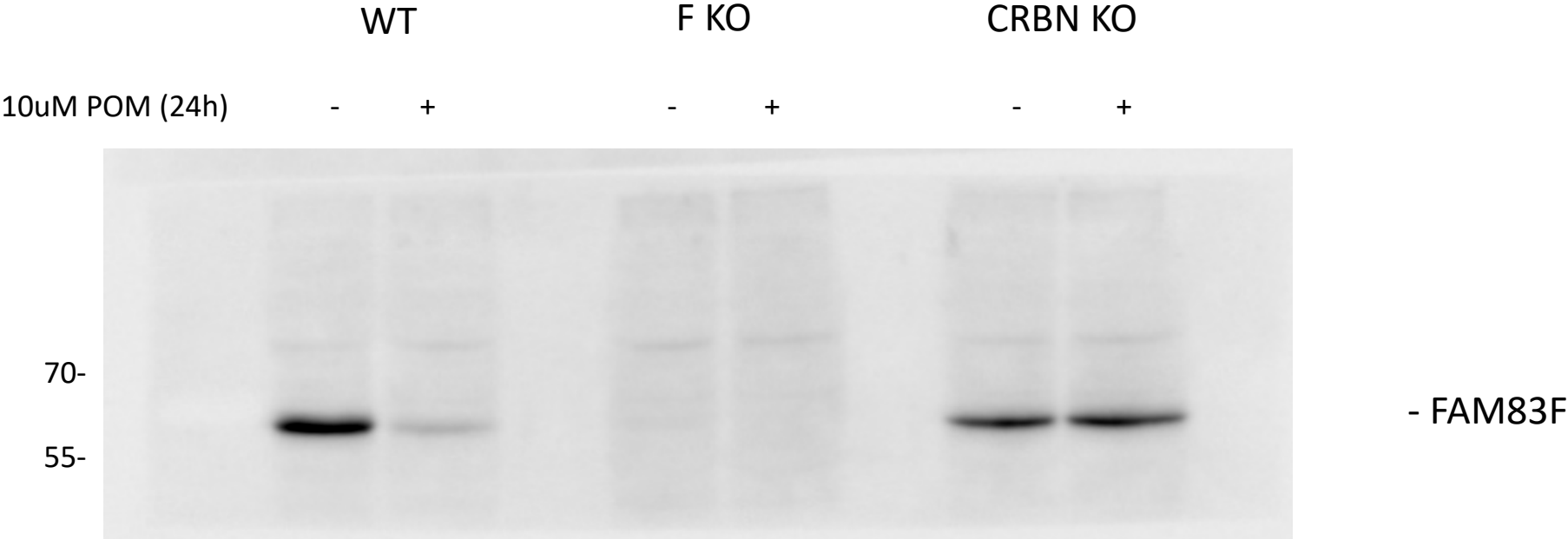

Figure 7B.

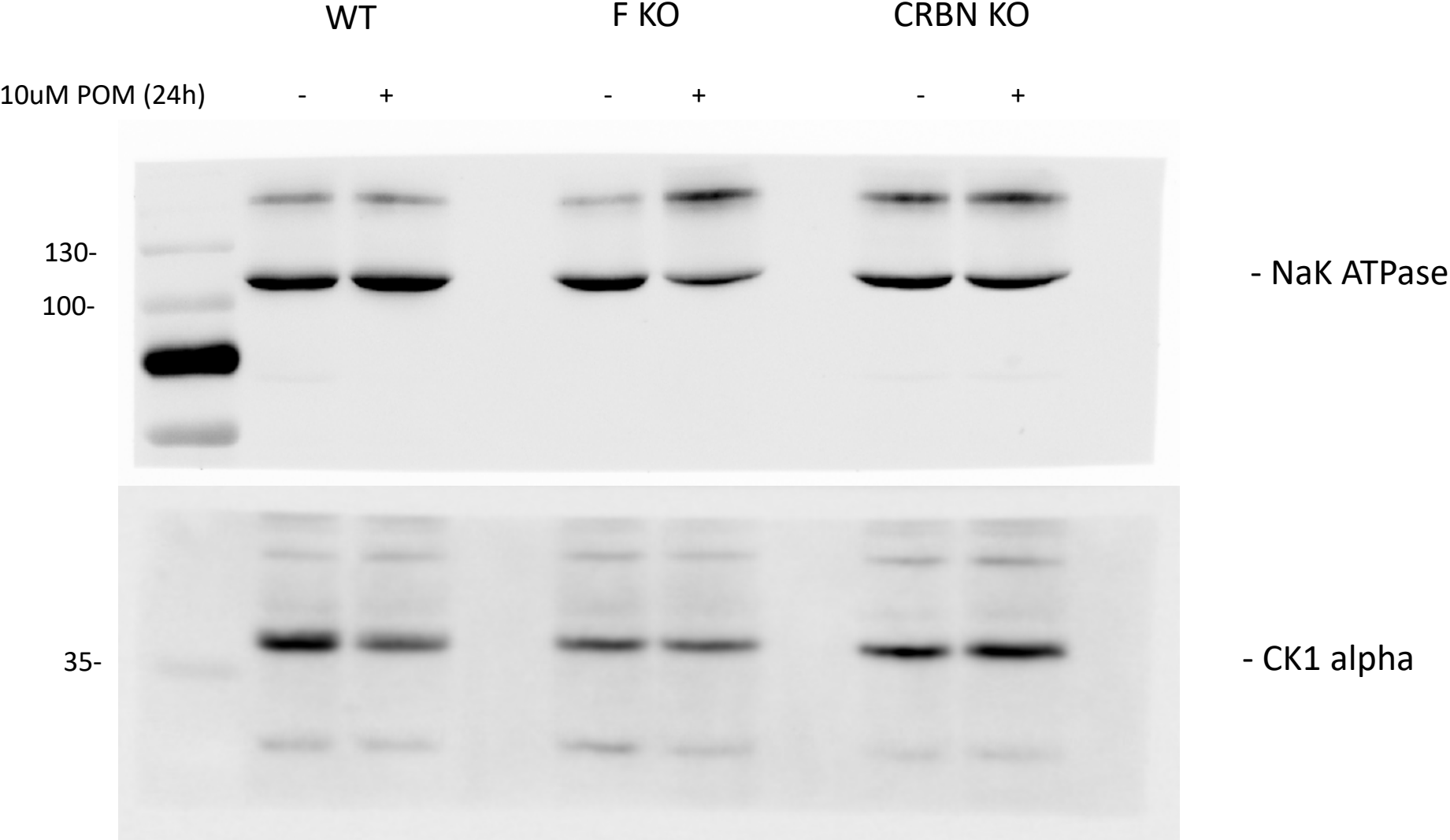

Figure 7D.

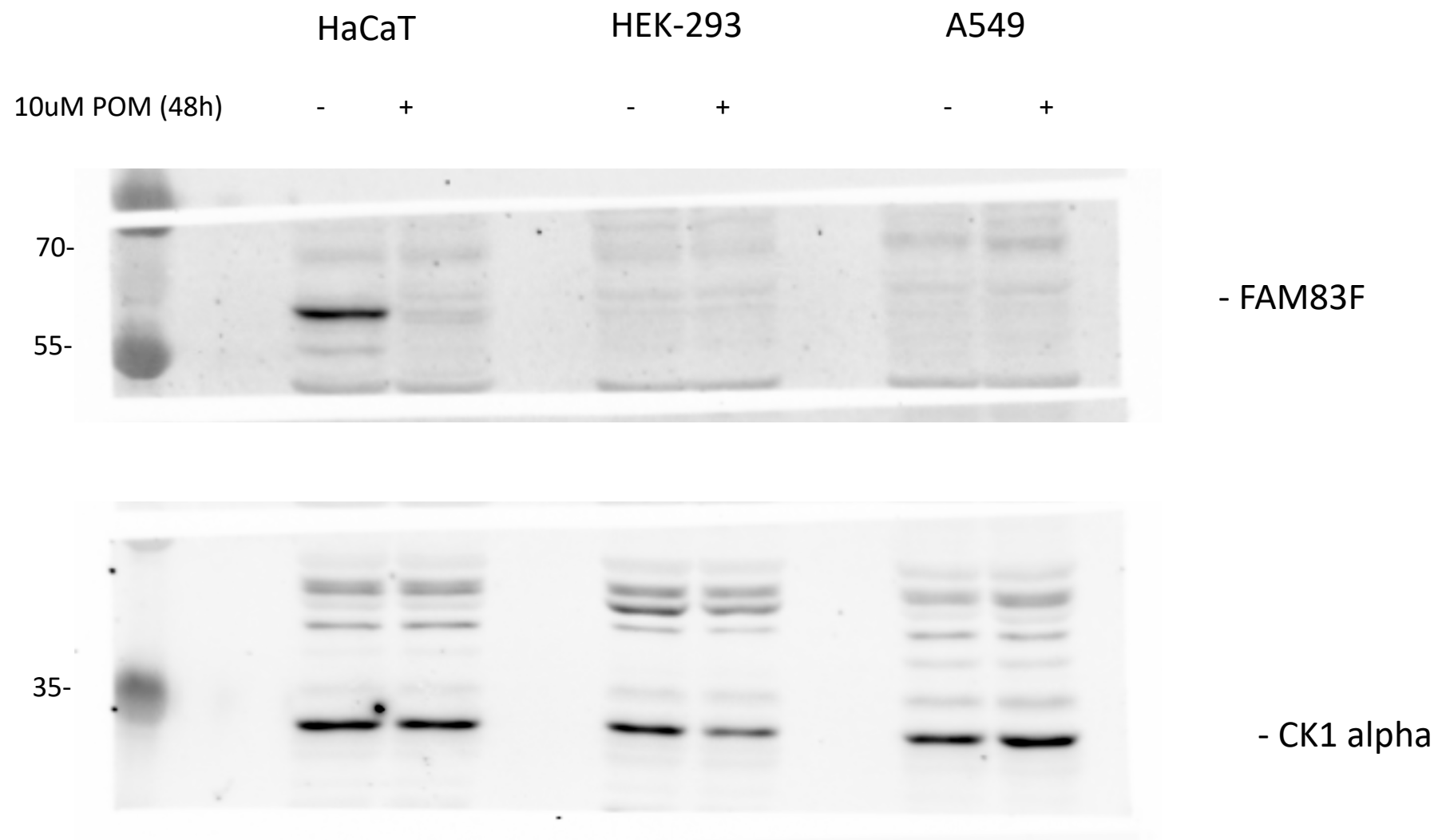

Figure 7D.

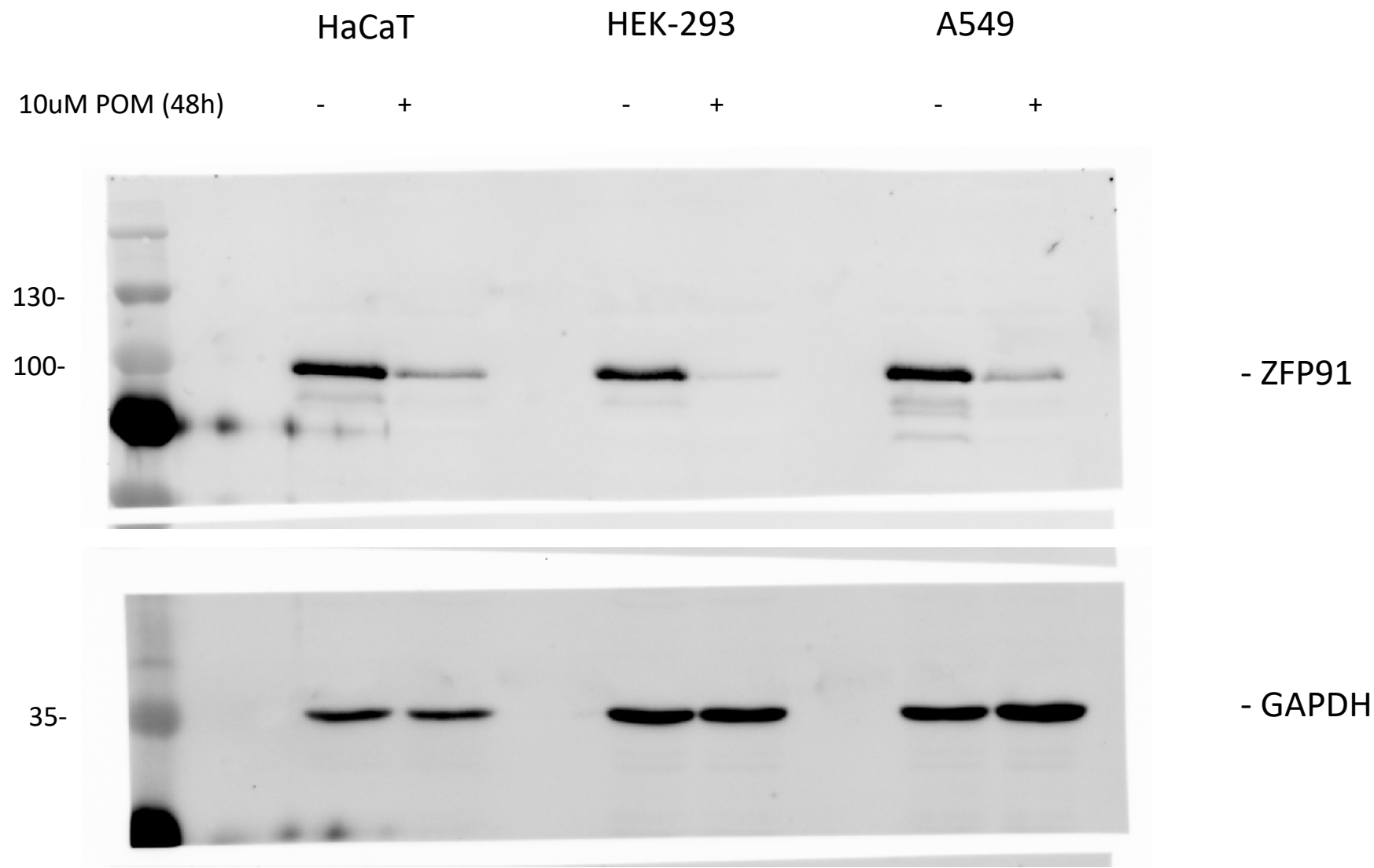

Supplement: Supplementary file 12 [file LSA-2020-00804_SdataF7.pdf]
