# Supplementary material for: IMiDs induce FAM83F degradation via an interaction with CK1α to attenuate Wnt signalling
Source: Life Sci Alliance. 2020 Dec 23;4(2):e202000804. doi: 10.26508/lsa.202000804 (PMC7768194; doi:10.26508/lsa.202000804)
Supplement: Supplementary file 6 [file LSA-2020-00804_SdataF3.pdf]

Figure 3A.

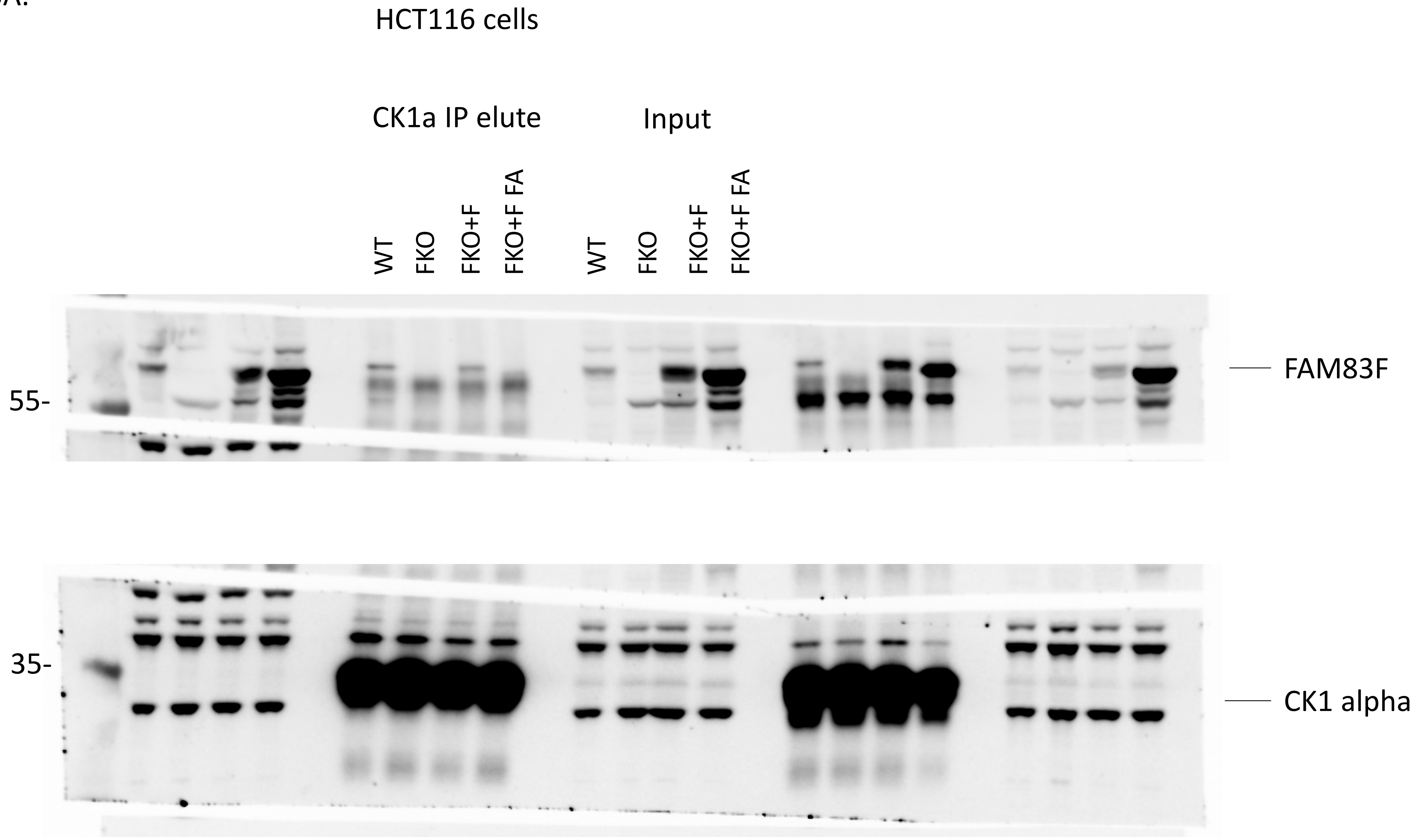

HCT116 cells
CK1a IP elute
Input
WT
FKO
FKO+F
FKO+F FA
WT
FKO
FKO+F
FKO+F FA
55-
FAM83F
35-
CK1 alpha

Figure 3A.

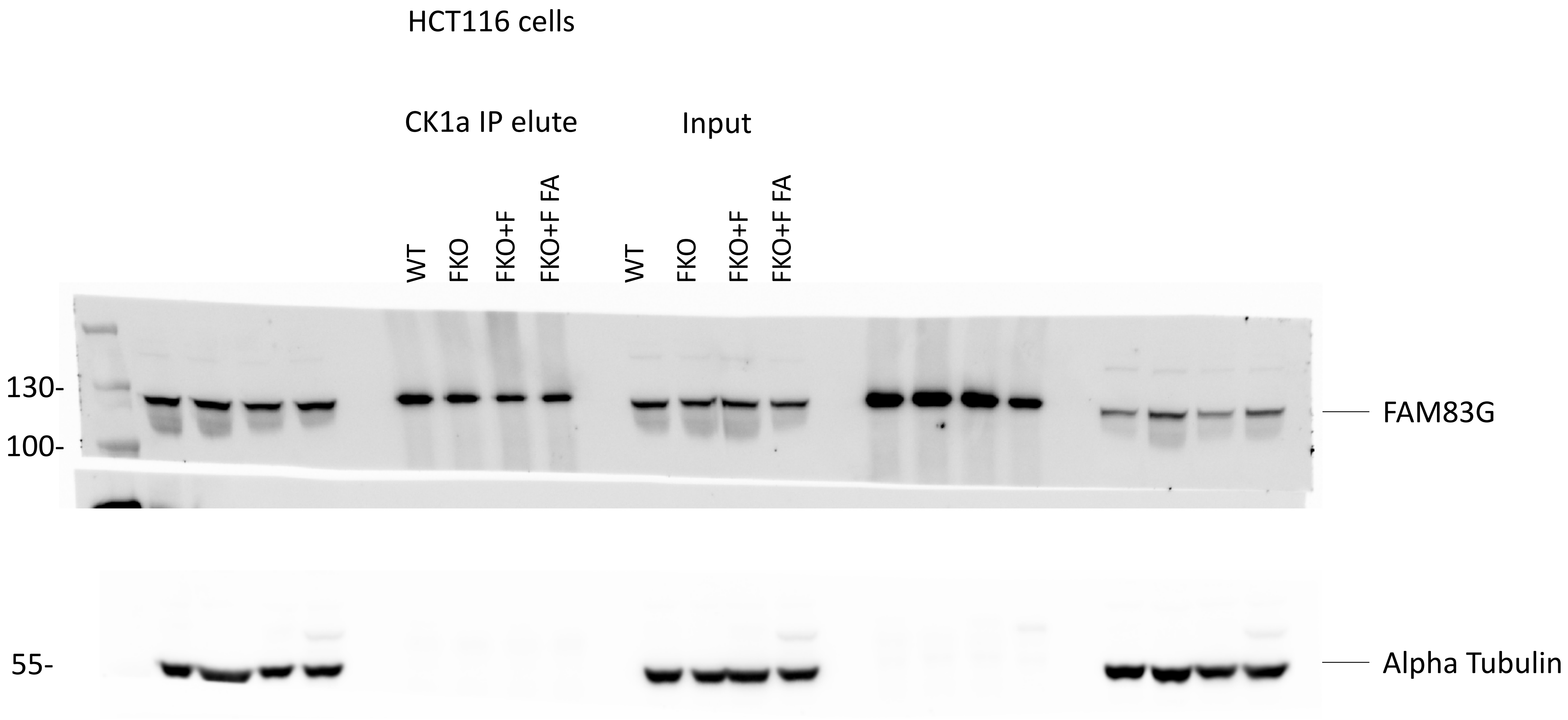
HCT116 cells
CK1a IP elute
Input
WT
FKO
FKO+F
FKO+F FA
WT
FKO
FKO+F
FKO+F FA
130-
100-
FAM83G
55-
Alpha Tubulin

Figure 3B.

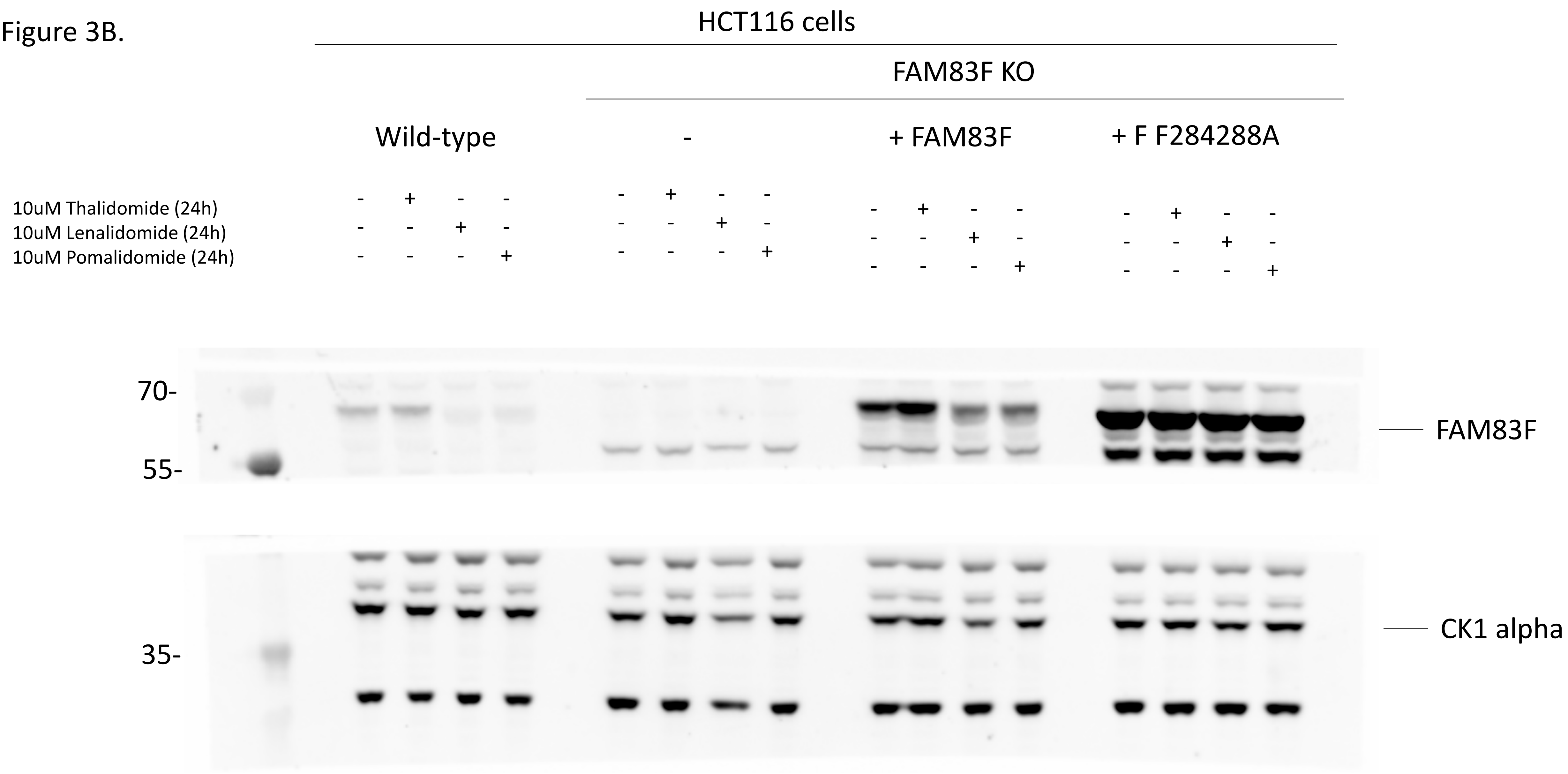
HCT116 cells
FAM83F KO
Wild-type
-
+ FAM83F
+ F F284288A
10uM Thalidomide (24h)
10uM Lenalidomide (24h)
10uM Pomalidomide (24h)
- + - -
- - + -
- - - +
70-
55-
35-
FAM83F
CK1 alpha

Figure 3B.

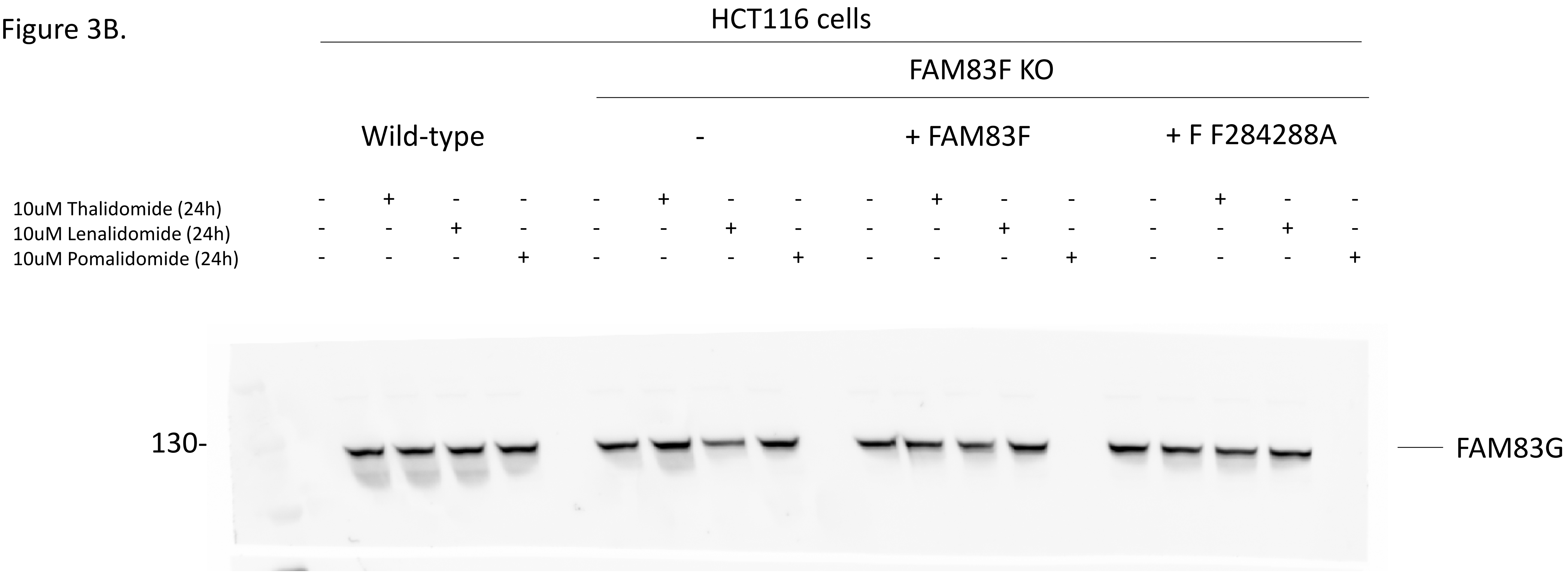
HCT116 cells
FAM83F KO
Wild-type
-
+ FAM83F
+ F F284288A
10uM Thalidomide (24h)
10uM Lenalidomide (24h)
10uM Pomalidomide (24h)
130-
FAM83G

Figure 3B.

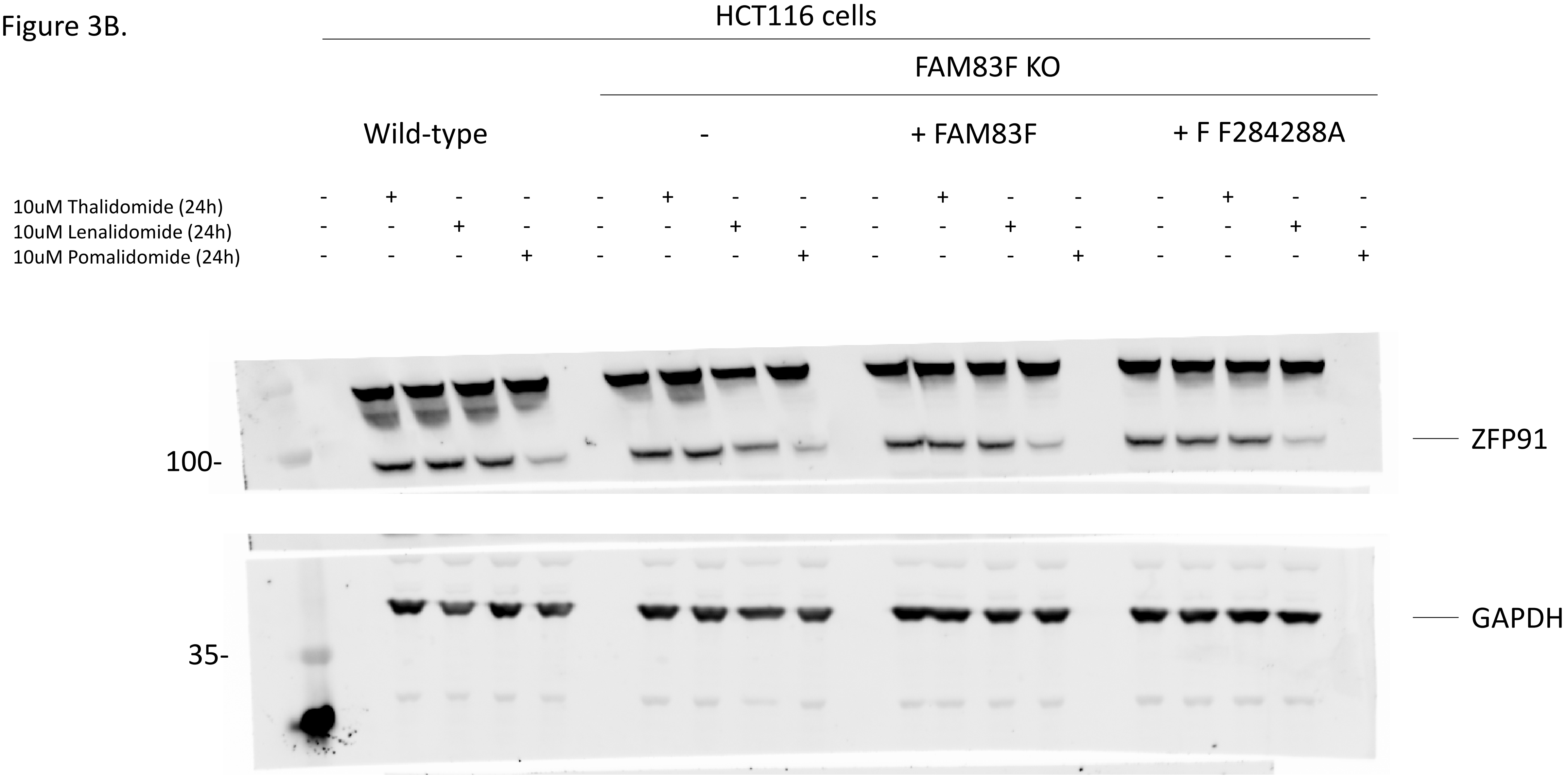
HCT116 cells
FAM83F KO
Wild-type
-
+ FAM83F
+ F F284288A
10uM Thalidomide (24h)
10uM Lenalidomide (24h)
10uM Pomalidomide (24h)
100-
35-
ZFP91
GAPDH
