# Supplementary material for: IMiDs induce FAM83F degradation via an interaction with CK1α to attenuate Wnt signalling
Source: Life Sci Alliance. 2020 Dec 23;4(2):e202000804. doi: 10.26508/lsa.202000804 (PMC7768194; doi:10.26508/lsa.202000804)
Supplement: Supplementary file 7 [file LSA-2020-00804_SdataF4.pdf]

Figure 4A.

DLD-1

10uM Thalidomide	-	+	+	+	-	-	-	-	-	-
10uM Lenalidomide	-	-	-	-	+	+	+	-	-	-
10uM Pomalidomide	-	-	-	-	-	-	-	+	+	+
1uM MLN4924	-	-	+	-	-	+	-	-	+	-
5uM Bortezamib	-	-	-	+	-	-	+	-	-	+

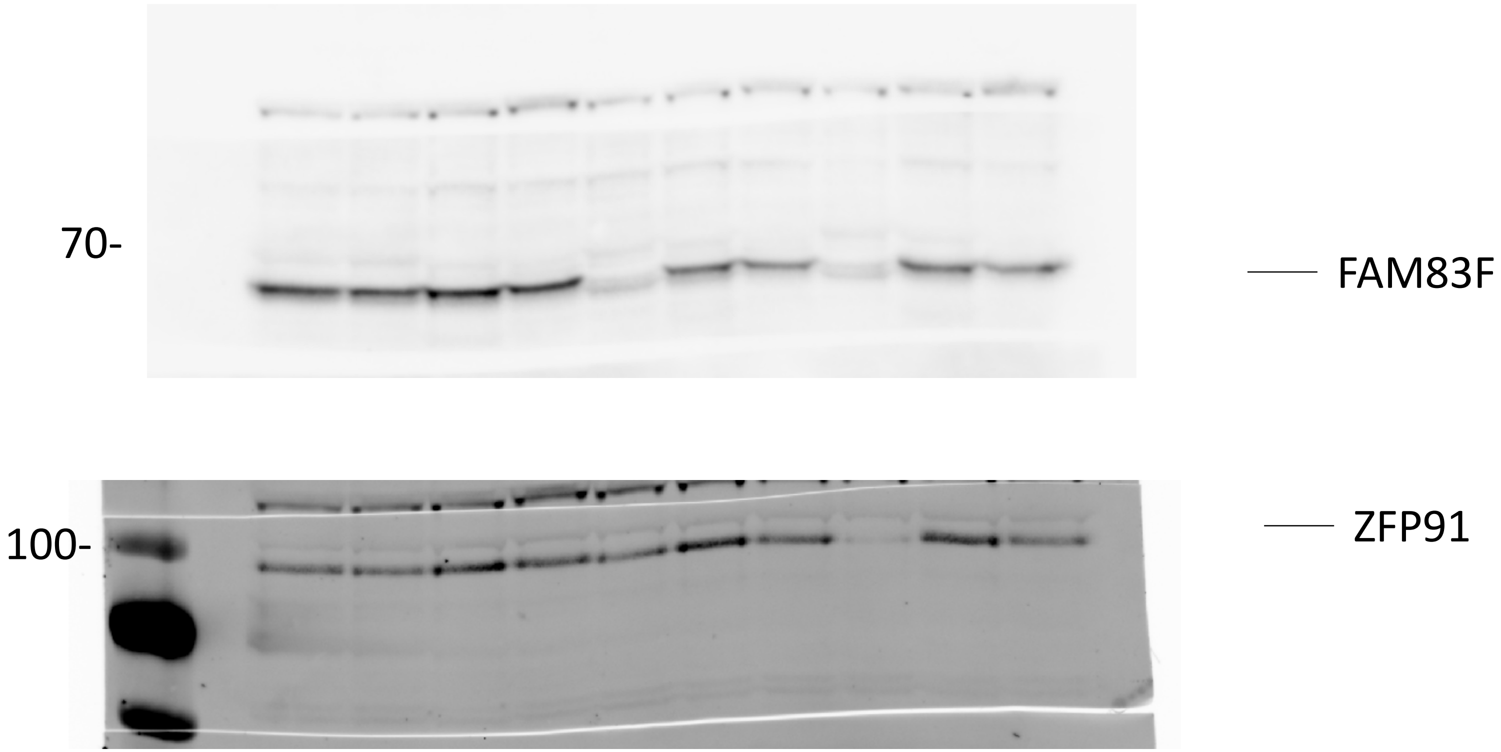

Figure 4A.

	DLD-1									
10uM Thalidomide	-	+	+	+	-	-	-	-	-	-
10uM Lenalidomide	-	-	-	-	+	+	+	-	-	-
10uM Pomalidomide	-	-	-	-	-	-	-	+	+	+
1uM MLN4924	-	-	+	-	-	+	-	-	+	-
5uM Bortezamib	-	-	-	+	-	-	+	-	-	+

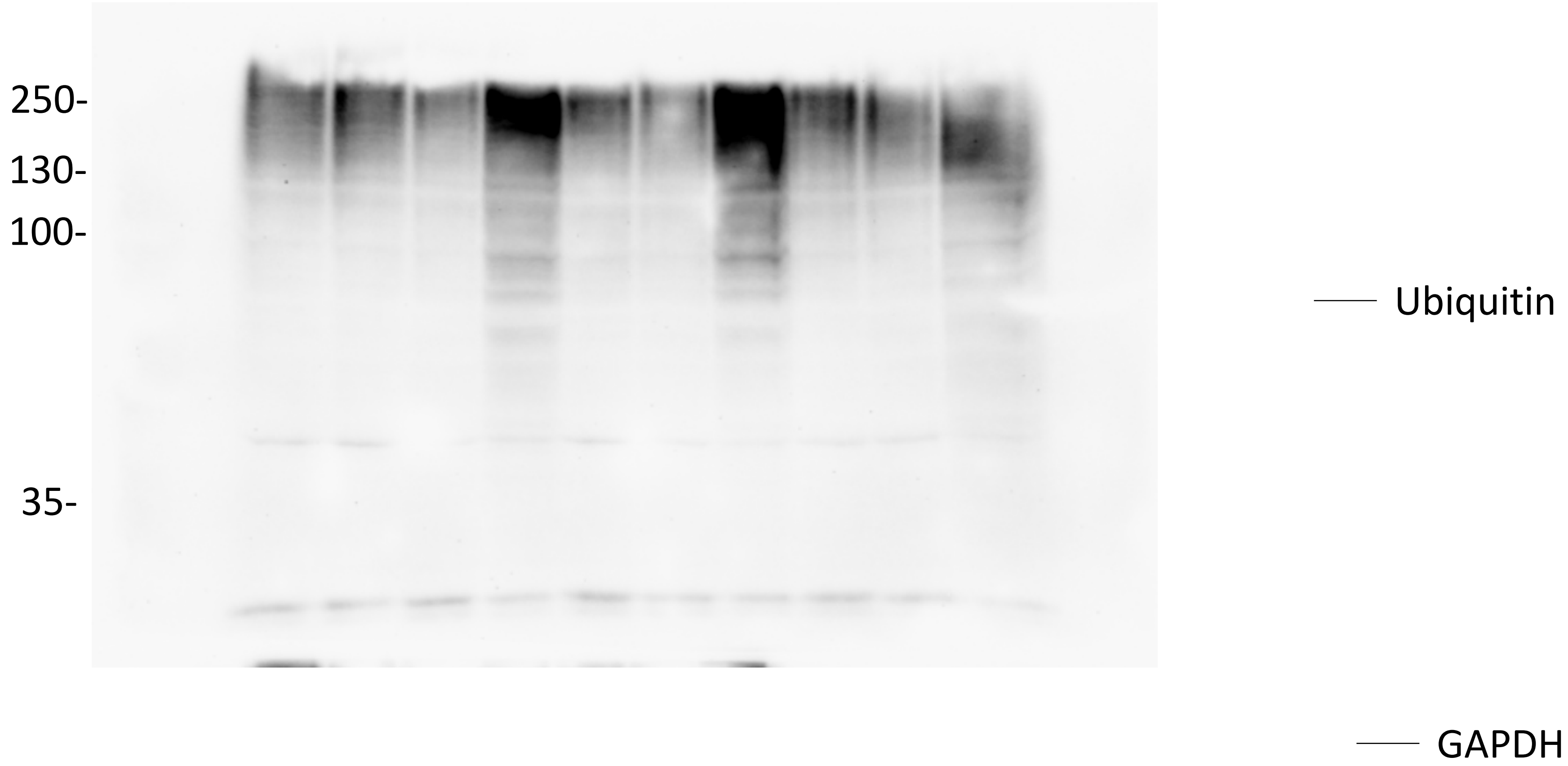

Figure 4A.

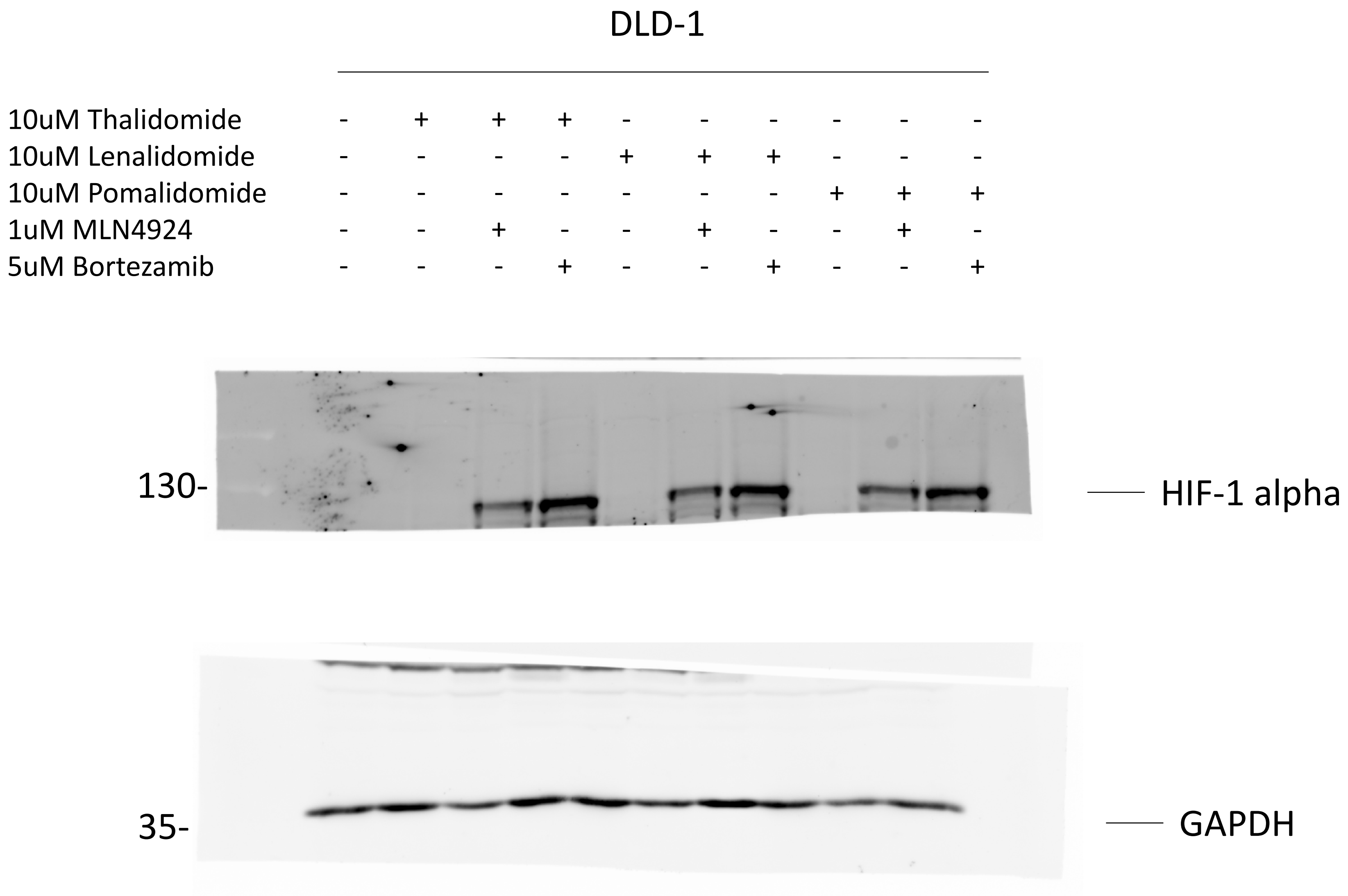
DLD-1
10uM Thalidomide - + + + - - - - - -
10uM Lenalidomide - - - - + + + - - -
10uM Pomalidomide - - - - - - - + + +
1uM MLN4924 - - + - - + - - + -
5uM Bortezamib - - - + - - + - - +
130-
HIF-1 alpha
35-
GAPDH

Figure 4A.

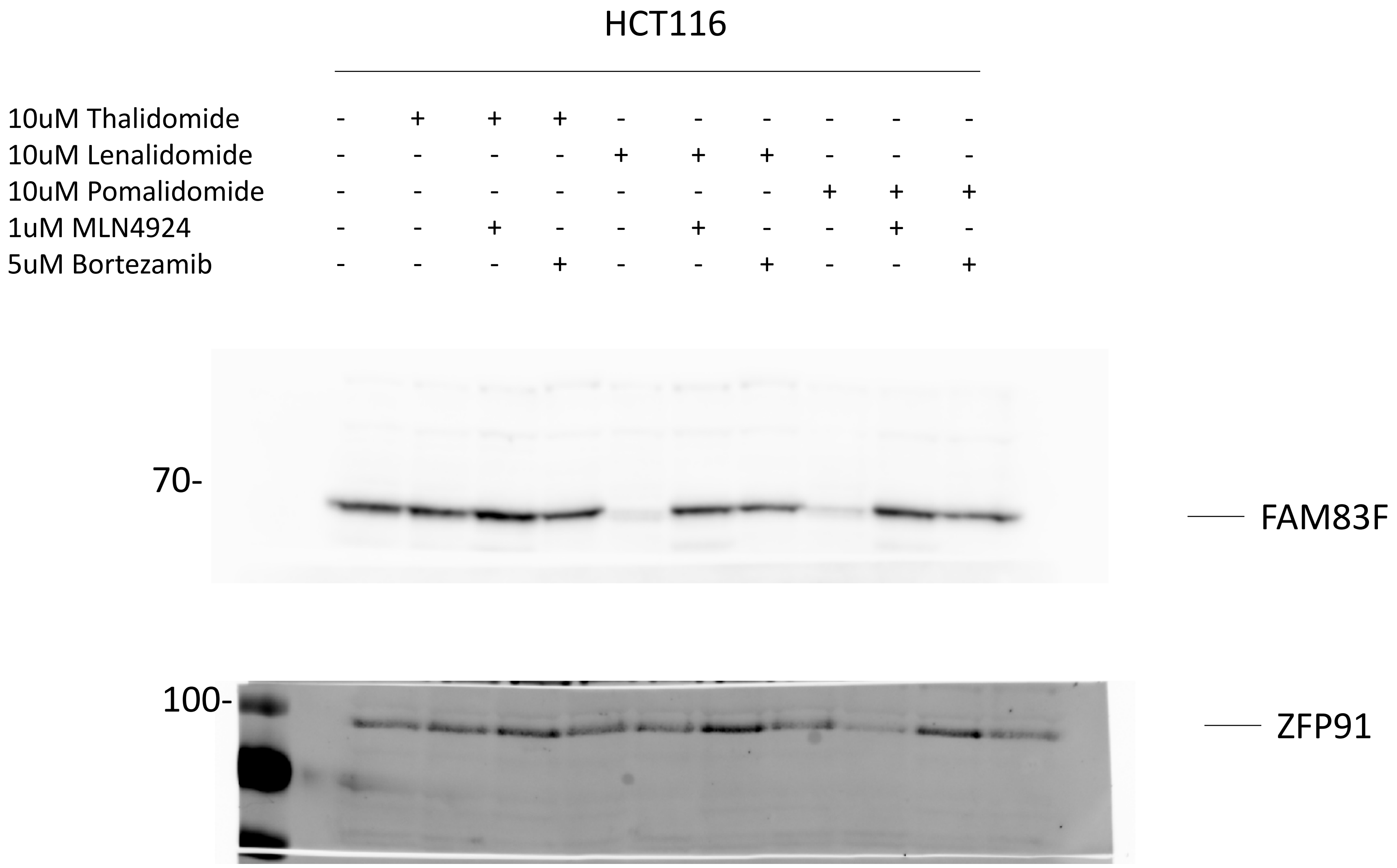
HCT116
10uM Thalidomide - + + + - - - - - -
10uM Lenalidomide - - - - + + + - - -
10uM Pomalidomide - - - - - - - + + +
1uM MLN4924 - - + - - + - - + -
5uM Bortezamib - - - + - - + - - +
70-
FAM83F
100-
ZFP91

Figure 4A.

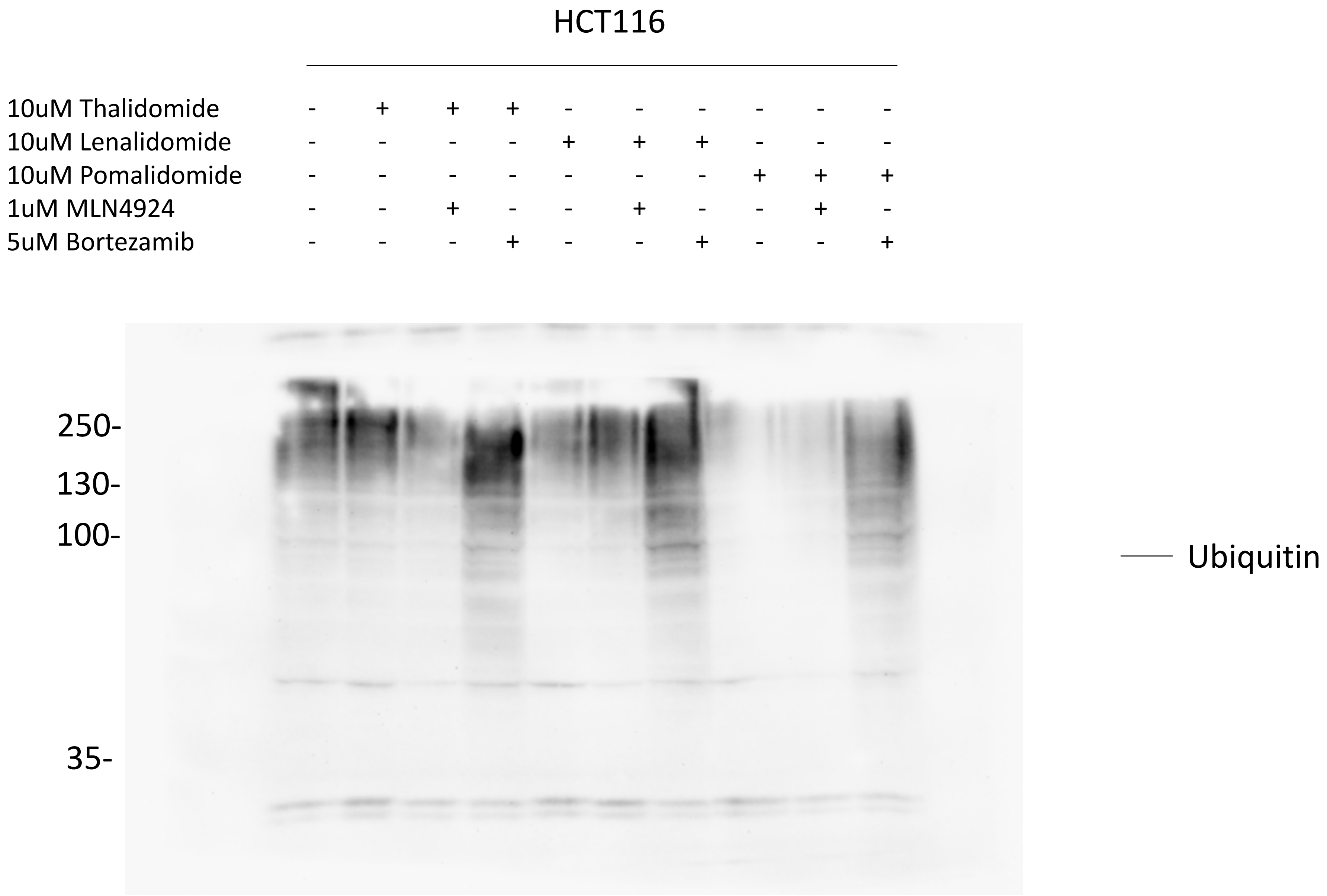
HCT116
10uM Thalidomide - + + + - - - - - -
10uM Lenalidomide - - - - + + + - - -
10uM Pomalidomide - - - - - - - + + +
1uM MLN4924 - - + - - + - - + -
5uM Bortezamib - - - + - - + - - +
250-
130-
100-
35-
Ubiquitin

Figure 4A.

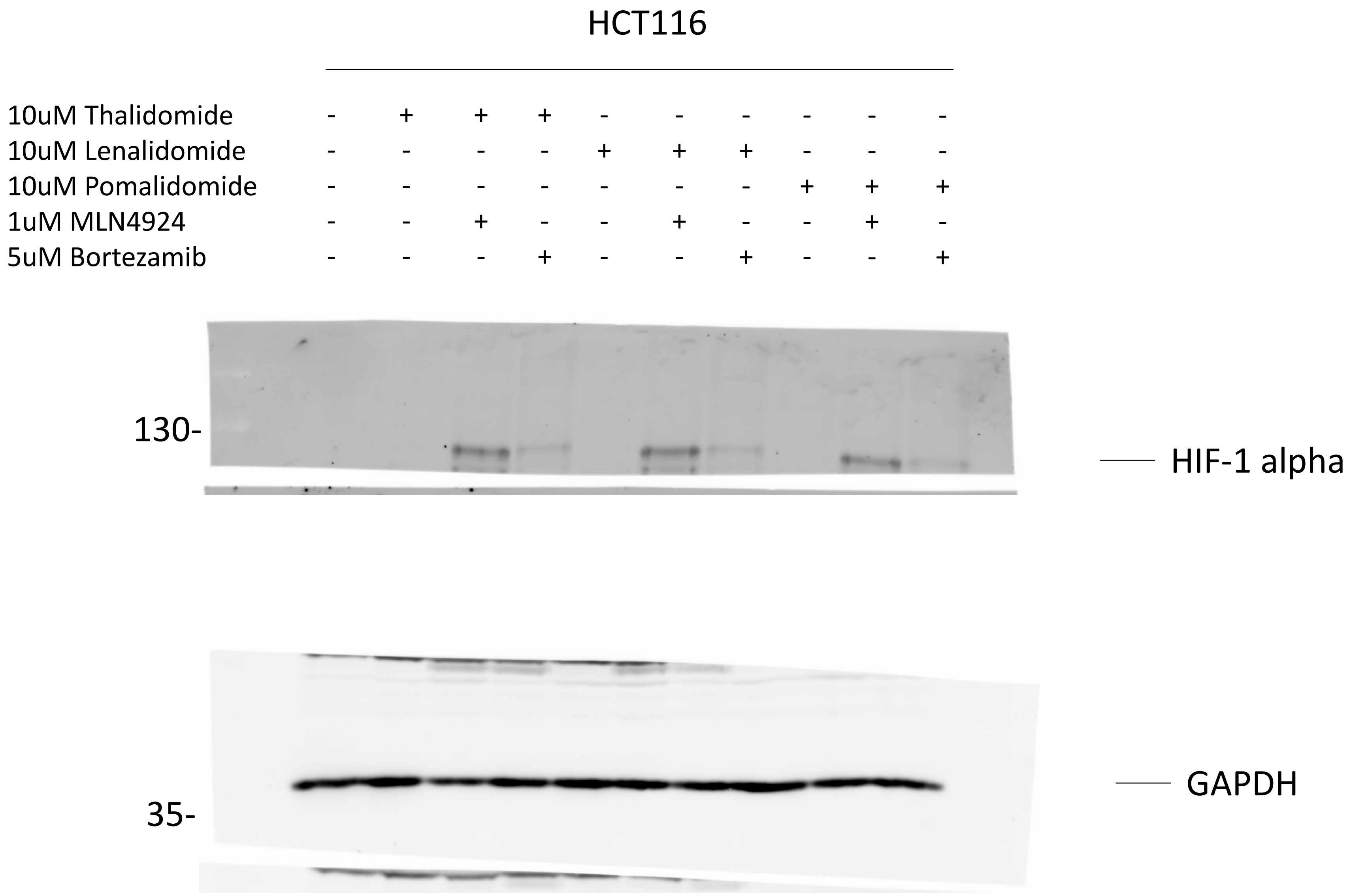
HCT116
10uM Thalidomide - + + + - - - - - -
10uM Lenalidomide - - - - + + + - - -
10uM Pomalidomide - - - - - - - + + +
1uM MLN4924 - - + - - + - - + -
5uM Bortezamib - - - + - - + - - +
130-
HIF-1 alpha
35-
GAPDH

Figure 4B.

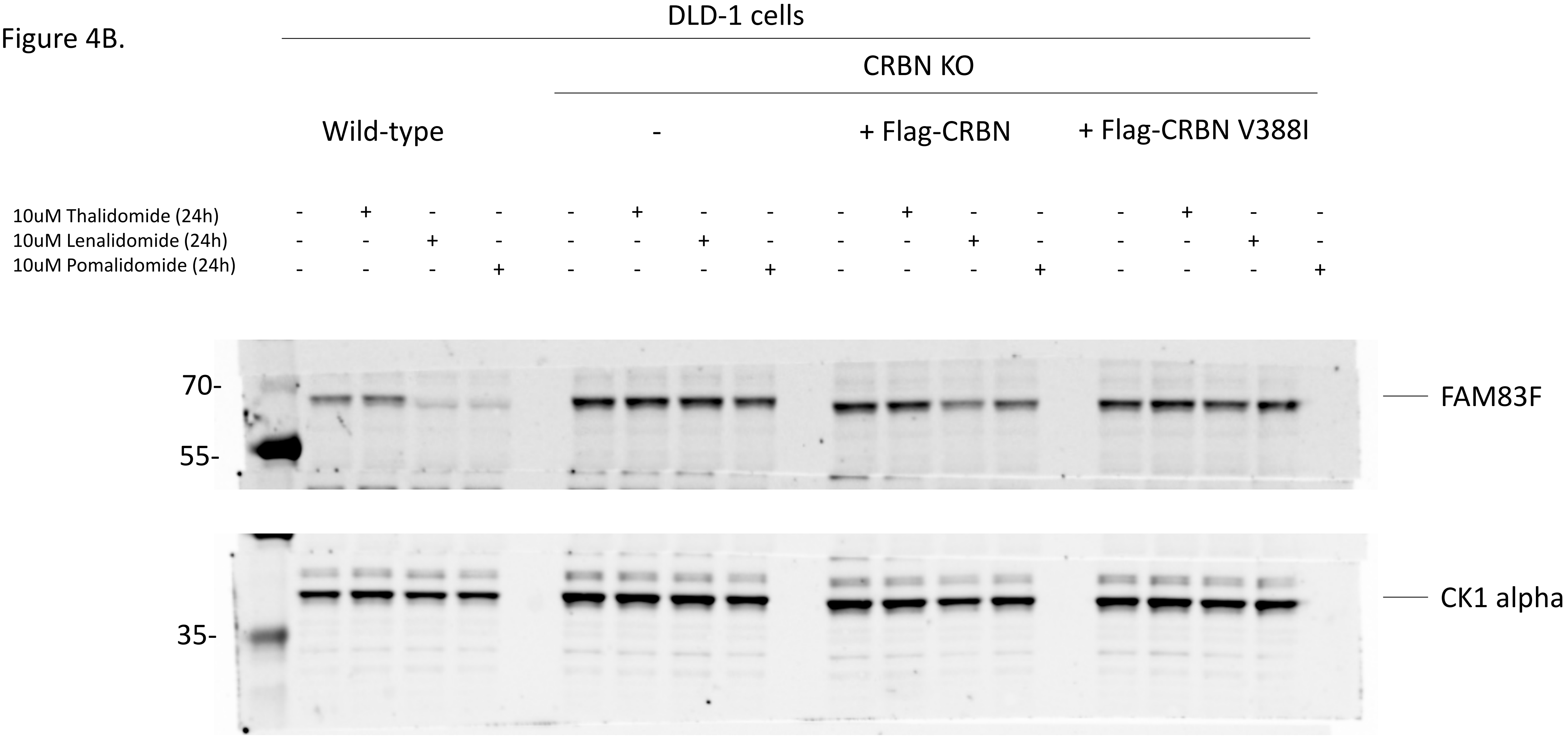
DLD-1 cells
CRBN KO
Wild-type
-
+ Flag-CRBN
+ Flag-CRBN V388I
10uM Thalidomide (24h)
10uM Lenalidomide (24h)
10uM Pomalidomide (24h)
70-
55-
FAM83F
35-
CK1 alpha

Figure 4B.

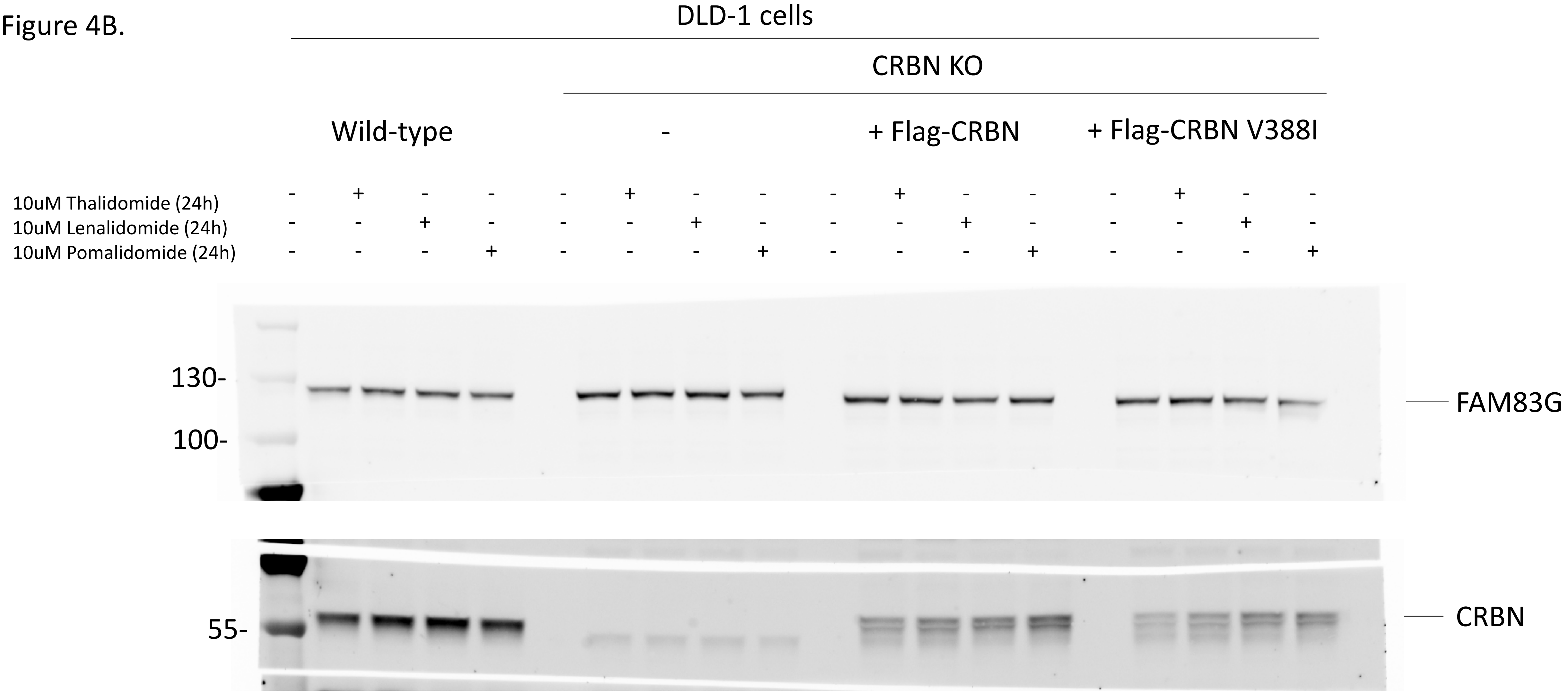
DLD-1 cells
CRBN KO
Wild-type
-
+ Flag-CRBN
+ Flag-CRBN V388I
10uM Thalidomide (24h)
10uM Lenalidomide (24h)
10uM Pomalidomide (24h)
130-
100-
FAM83G
55-
CRBN

Figure 4B.

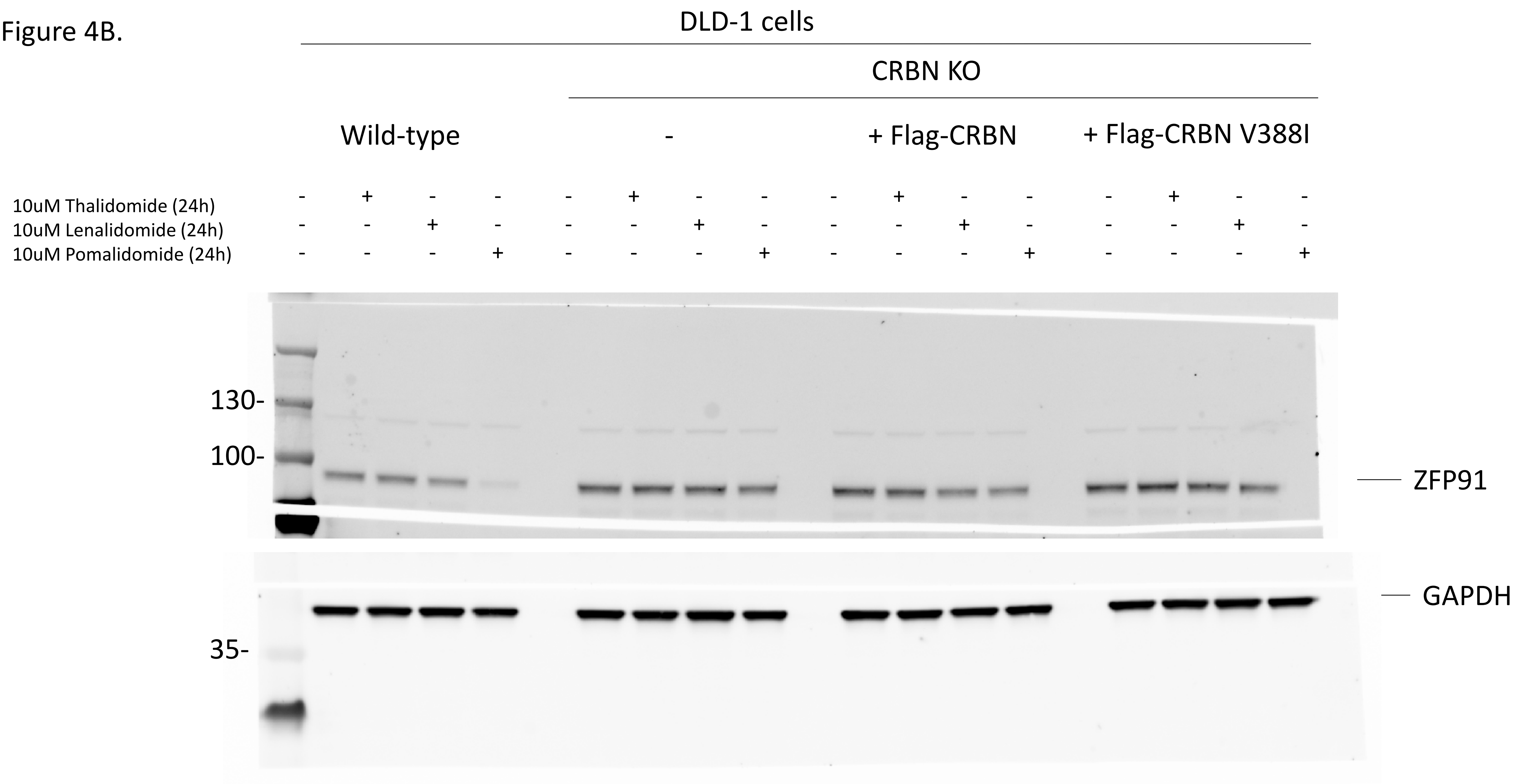
DLD-1 cells
CRBN KO
Wild-type
-
+ Flag-CRBN
+ Flag-CRBN V388I
10uM Thalidomide (24h) - + - - - + - - - + - - - + - -
10uM Lenalidomide (24h) - - + - - - + - - - + - - - + -
10uM Pomalidomide (24h) - - - + - - - + - - - + - - - +
130-
100-
ZFP91
GAPDH
35-
