# Supplementary material for: IMiDs induce FAM83F degradation via an interaction with CK1α to attenuate Wnt signalling
Source: Life Sci Alliance. 2020 Dec 23;4(2):e202000804. doi: 10.26508/lsa.202000804 (PMC7768194; doi:10.26508/lsa.202000804)

Sup. Figure 6A.

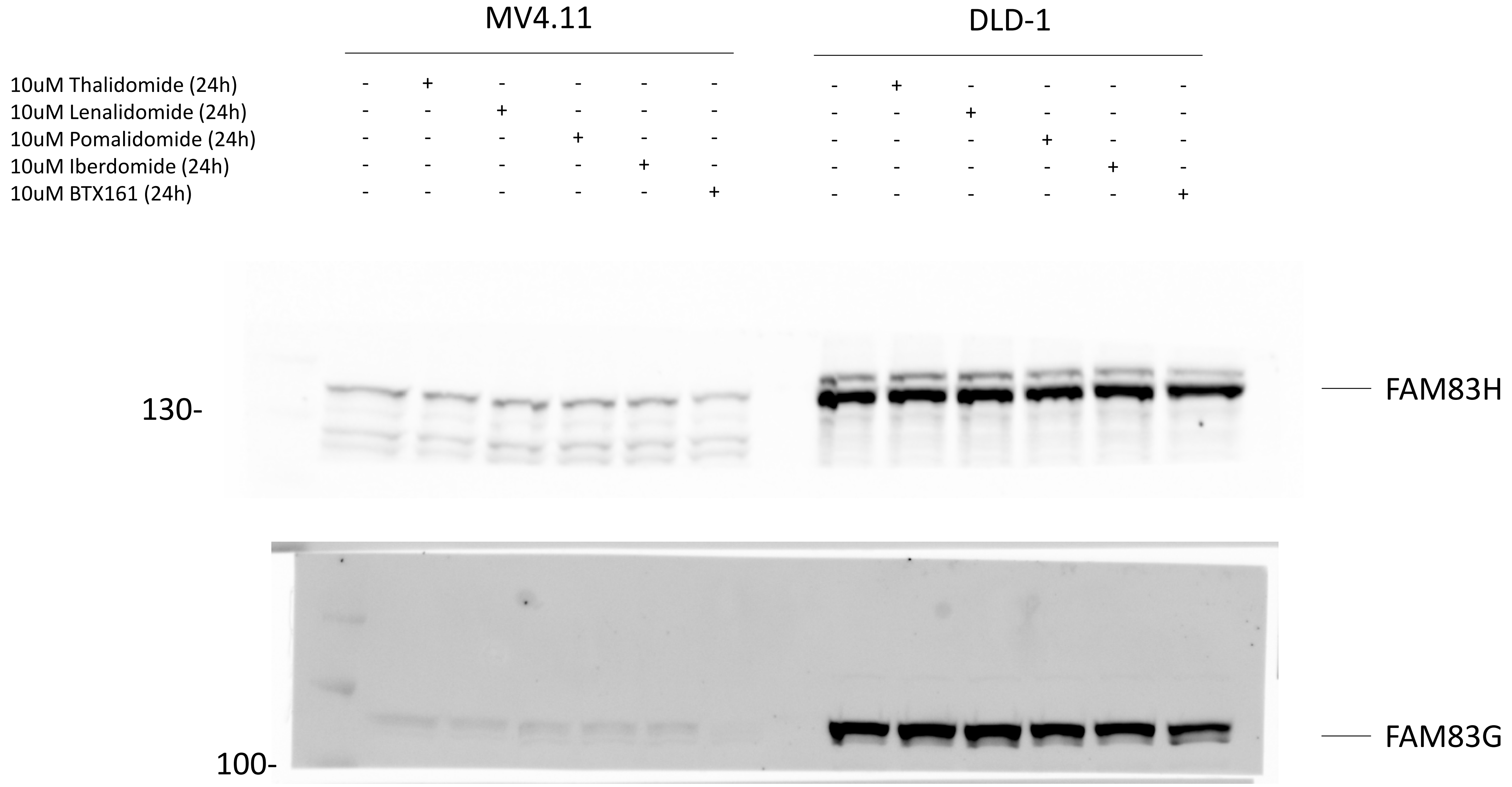

Sup. Figure 6A.

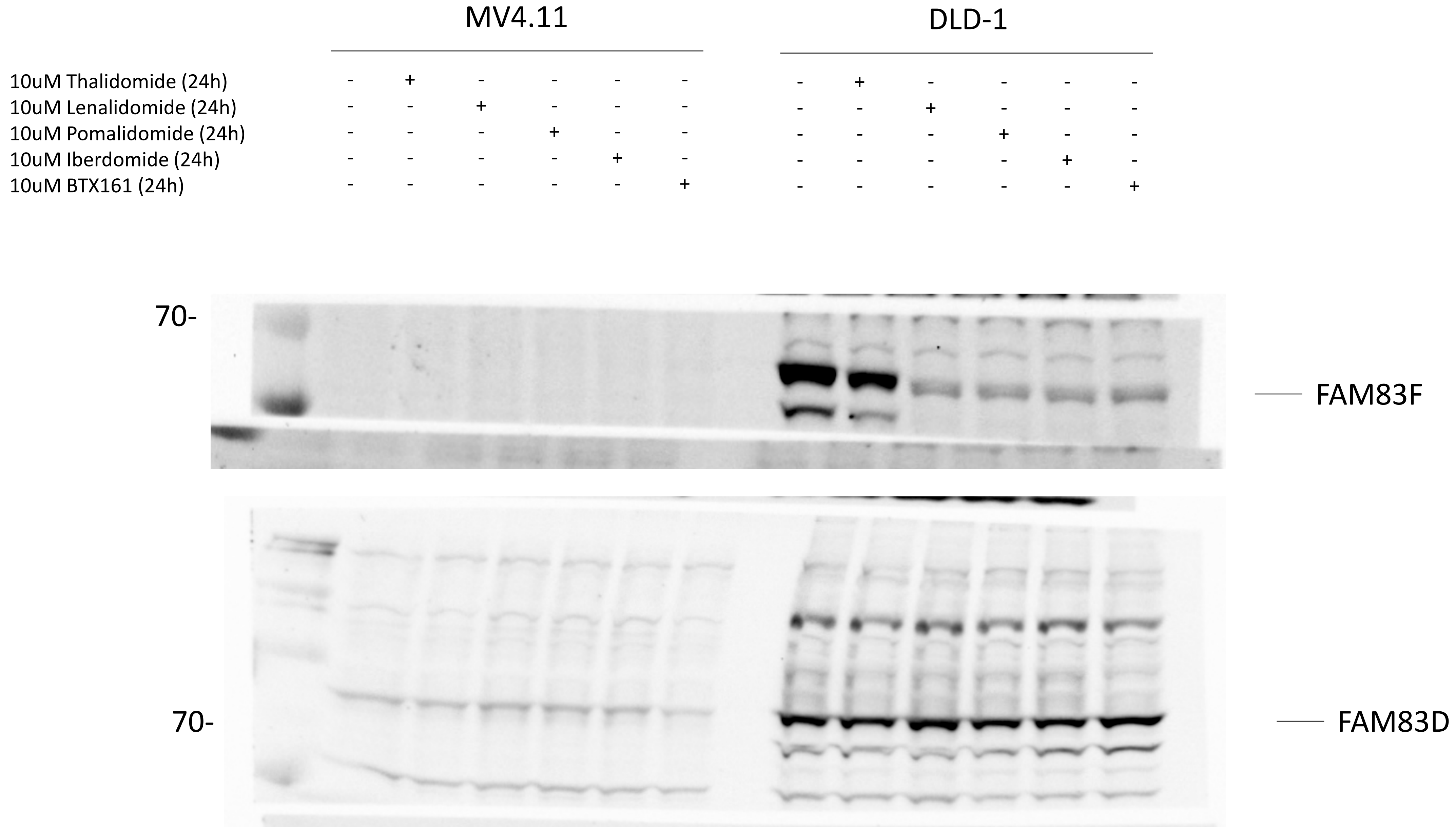

Sup. Figure 6A.

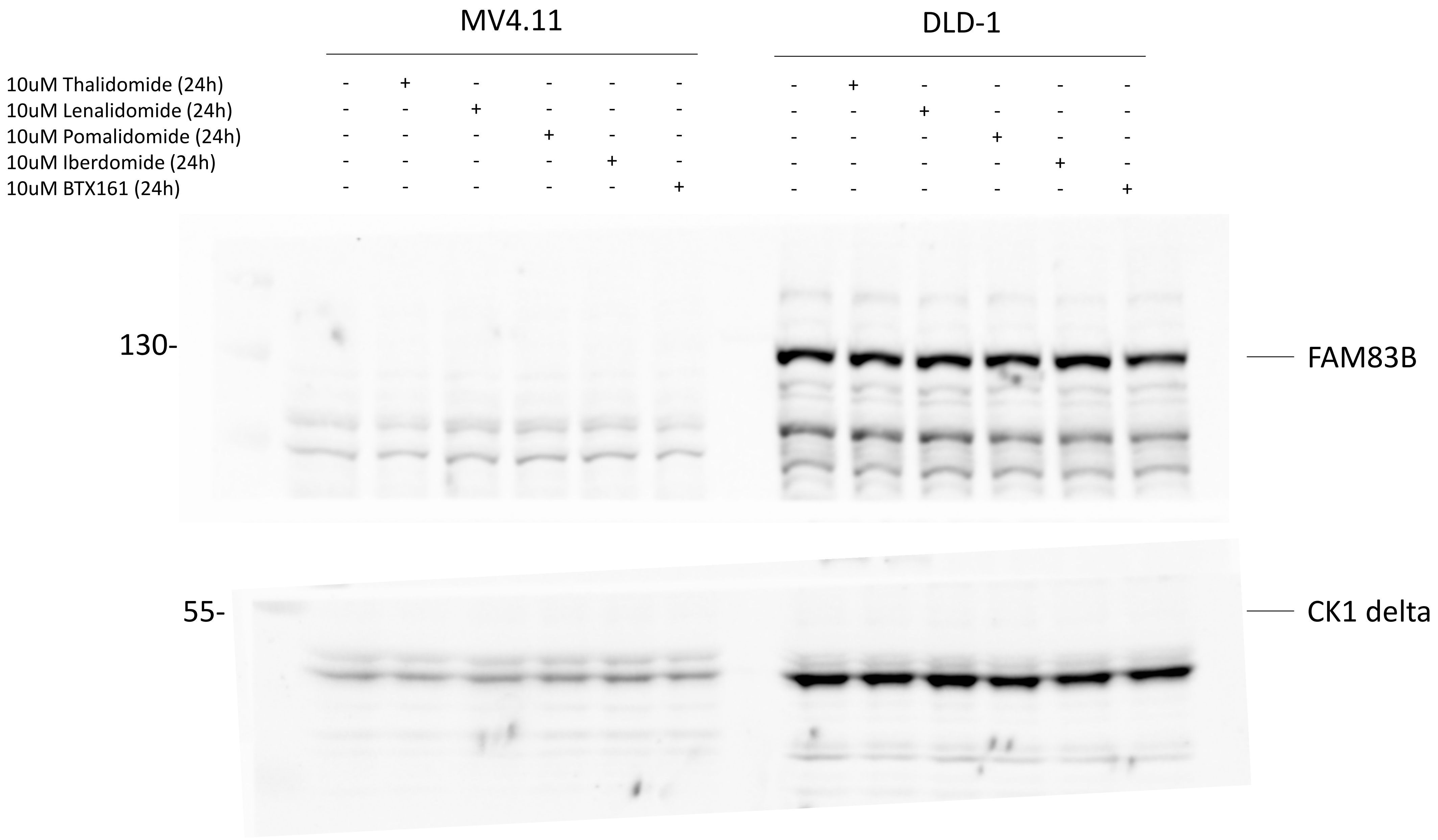

Sup. Figure 6A.

	MV4.11						DLD-1					
10uM Thalidomide (24h)	-	+	-	-	-	-	-	+	-	-	-	-
10uM Lenalidomide (24h)	-	-	+	-	-	-	-	-	+	-	-	-
10uM Pomalidomide (24h)	-	-	-	+	-	-	-	-	-	+	-	-
10uM Iberdomide (24h)	-	-	-	-	+	-	-	-	-	-	+	-
10uM BTX161 (24h)	-	-	-	-	-	+	-	-	-	-	-	+

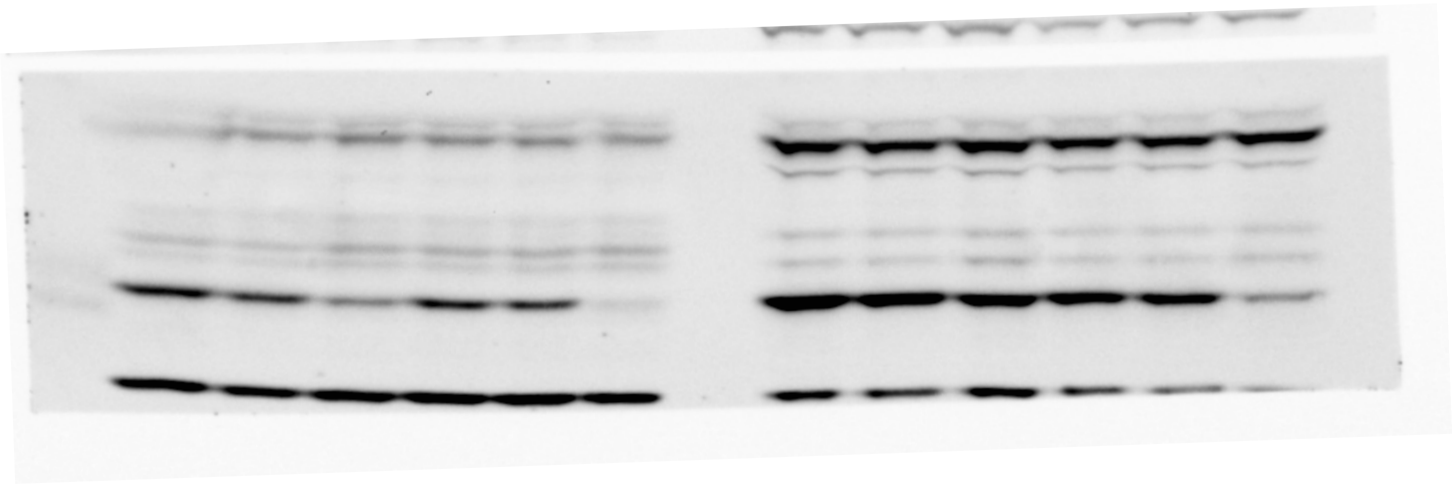

Sup. Figure 6A.

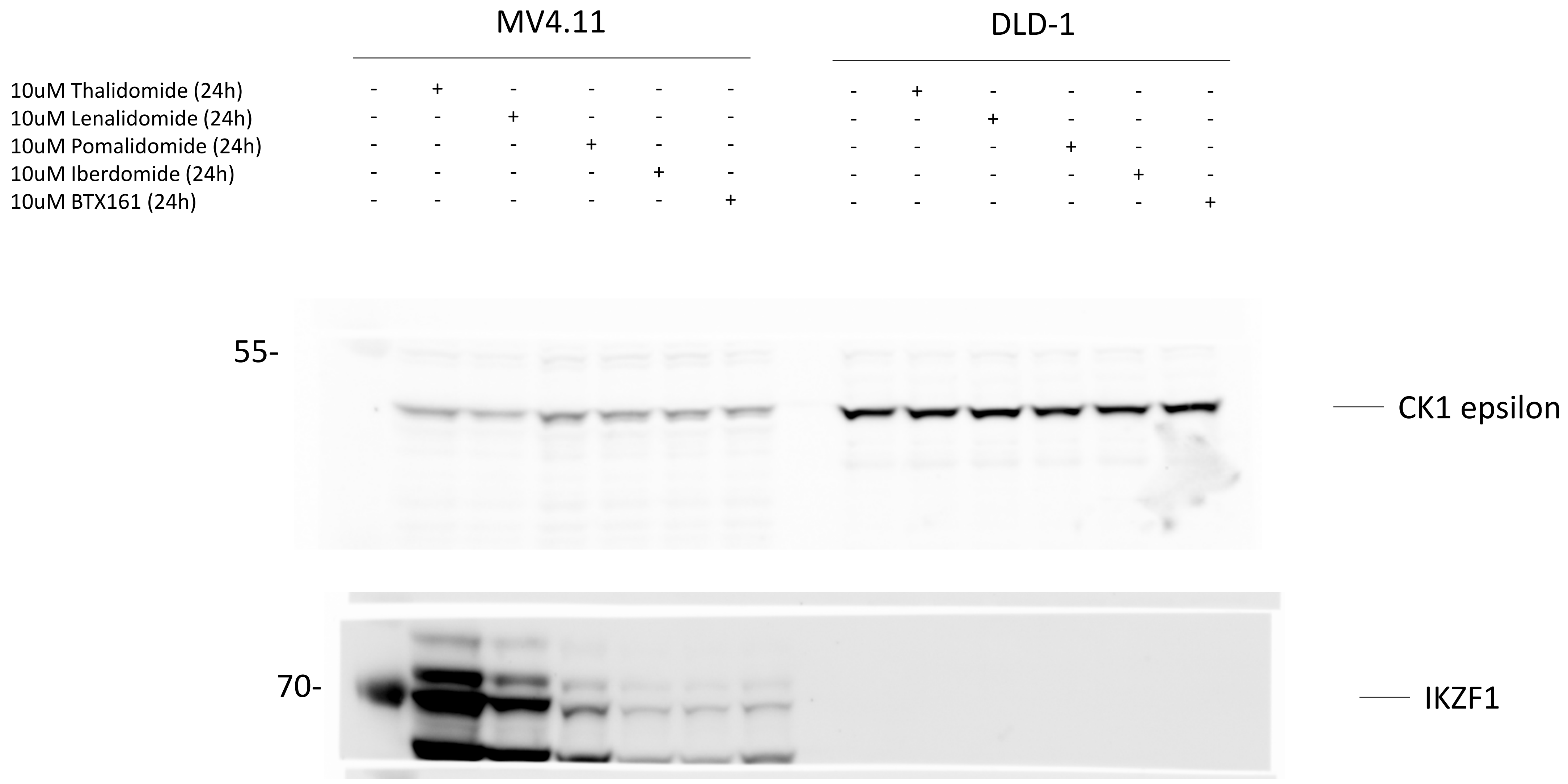

Sup. Figure 6A.

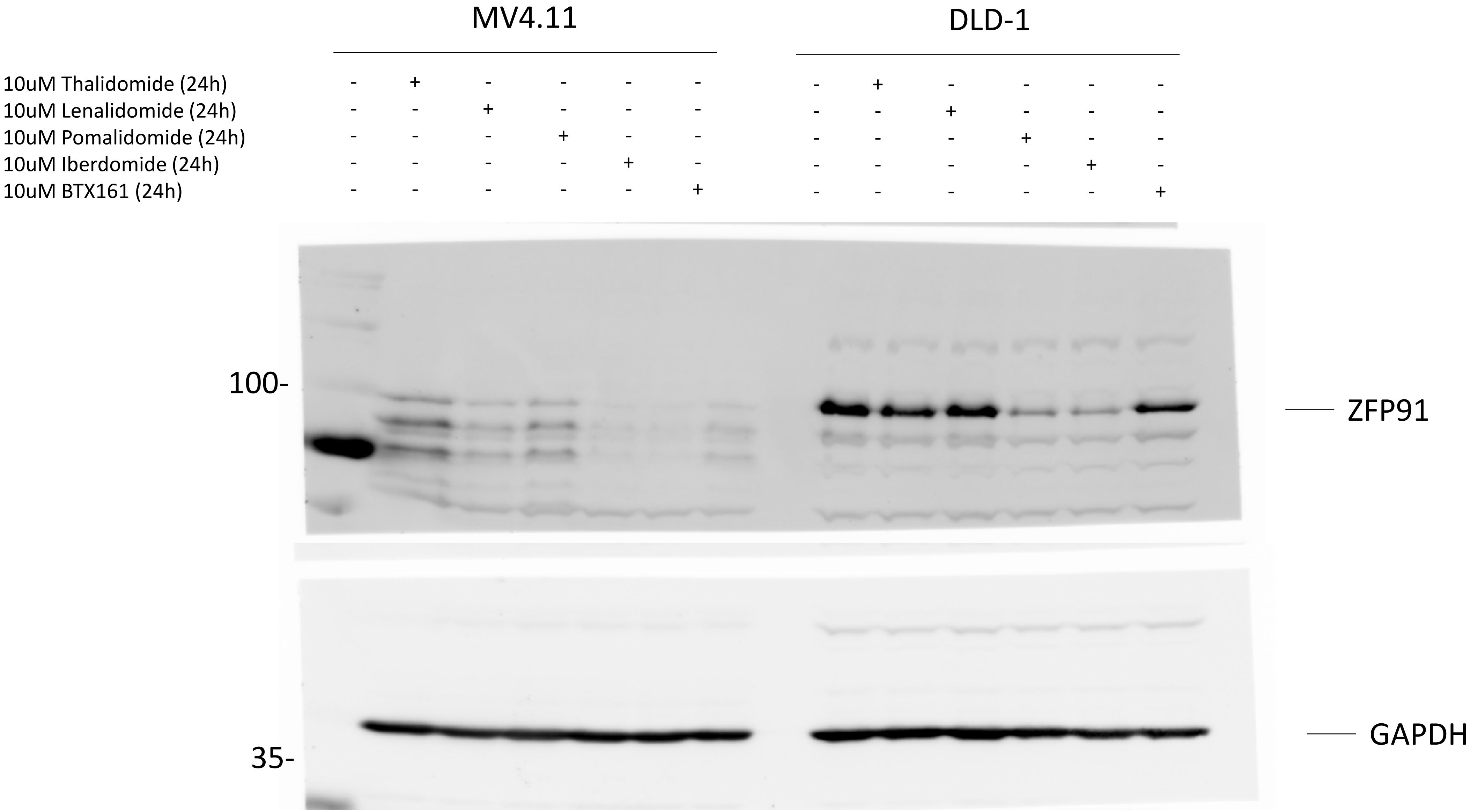

Supplement: Supplementary file 8 [file LSA-2020-00804_SdataFS6.pdf]
