# Supplementary material for: IMiDs induce FAM83F degradation via an interaction with CK1α to attenuate Wnt signalling
Source: Life Sci Alliance. 2020 Dec 23;4(2):e202000804. doi: 10.26508/lsa.202000804 (PMC7768194; doi:10.26508/lsa.202000804)
Supplement: Supplementary file 9 [file LSA-2020-00804_SdataF5.pdf]

Figure 5A.

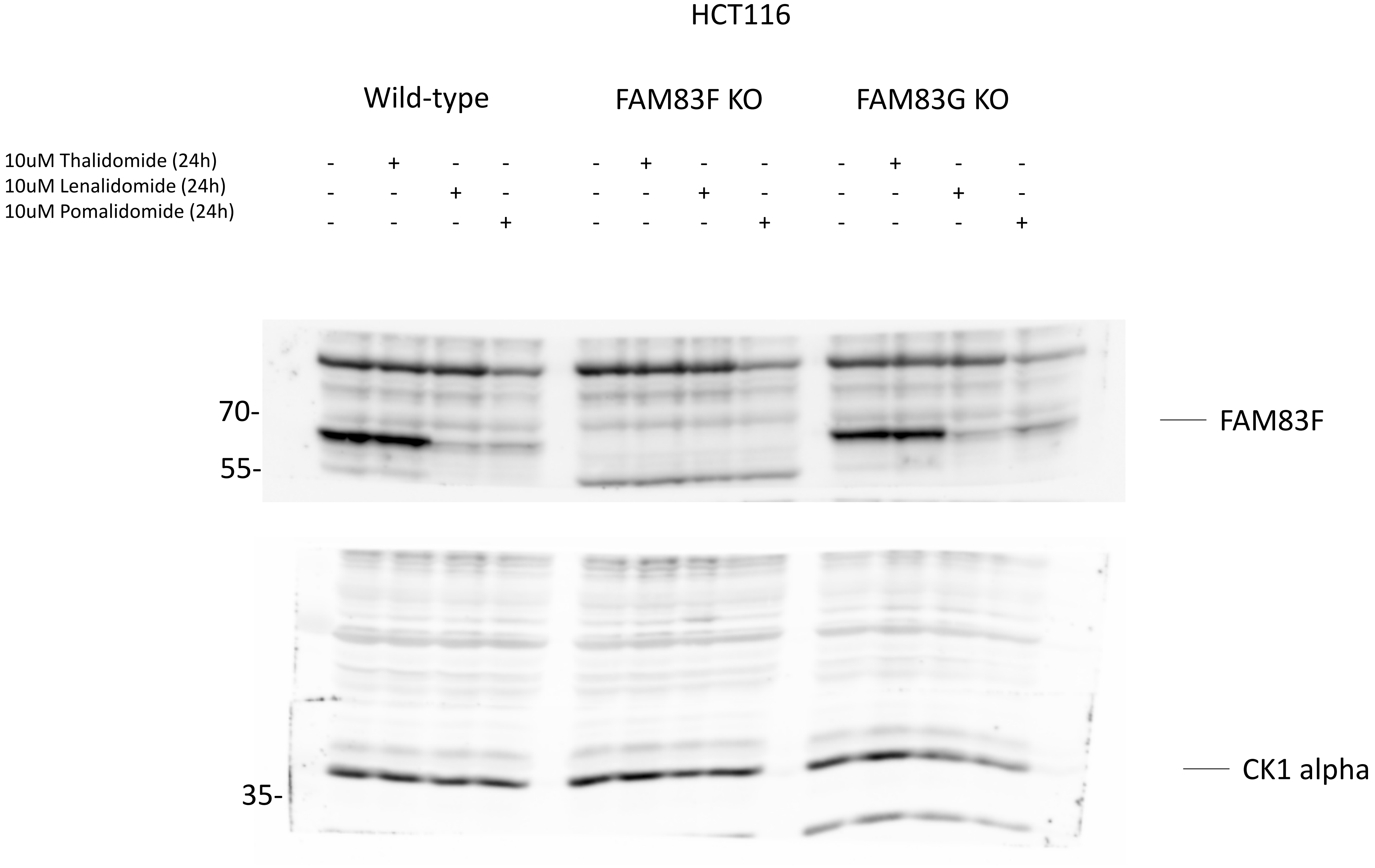
HCT116
Wild-type
FAM83F KO
FAM83G KO
10uM Thalidomide (24h)
10uM Lenalidomide (24h)
10uM Pomalidomide (24h)
- + - - - + - - - + - -
- - + - - - + - - - + -
- - - + - - - + - - - +
70-
55-
FAM83F
35-
CK1 alpha

Figure 5A.

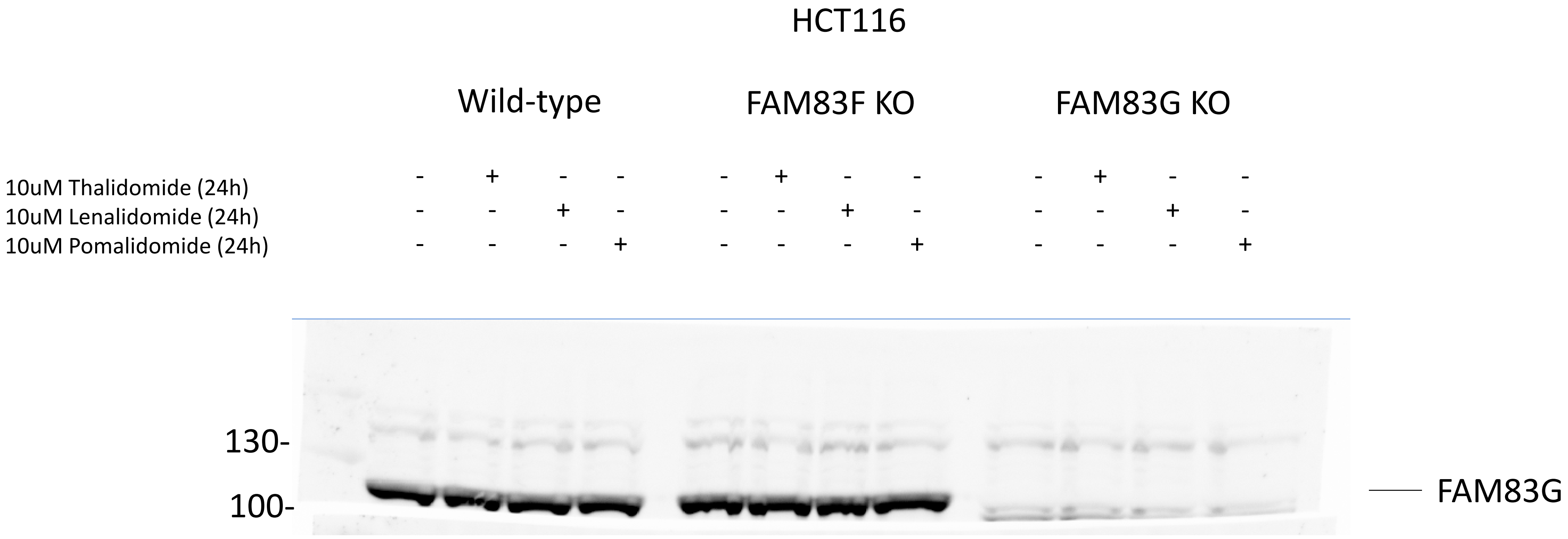

HCT116
Wild-type
FAM83F KO
FAM83G KO
10uM Thalidomide (24h)
10uM Lenalidomide (24h)
10uM Pomalidomide (24h)
- + - - - + - - - + - -
- - + - - - + - - - + -
- - - + - - - + - - - +
130-
100-
FAM83G

Figure 5A.

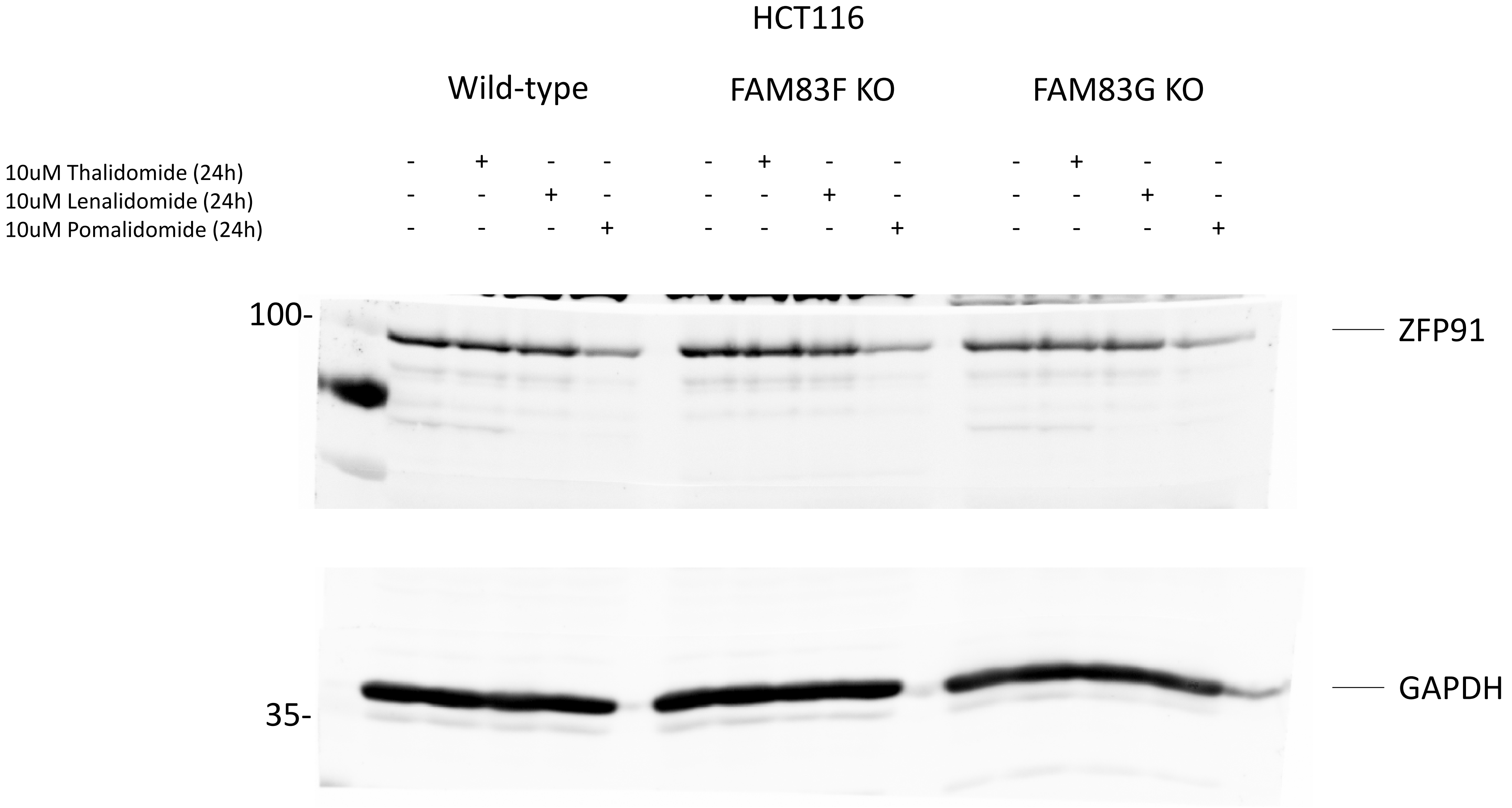
HCT116
Wild-type
FAM83F KO
FAM83G KO
10uM Thalidomide (24h) - + - - - + - - - + - -
10uM Lenalidomide (24h) - - + - - - + - - - + -
10uM Pomalidomide (24h) - - - + - - - + - - - +
100-
ZFP91
35-
GAPDH

Figure 5C.

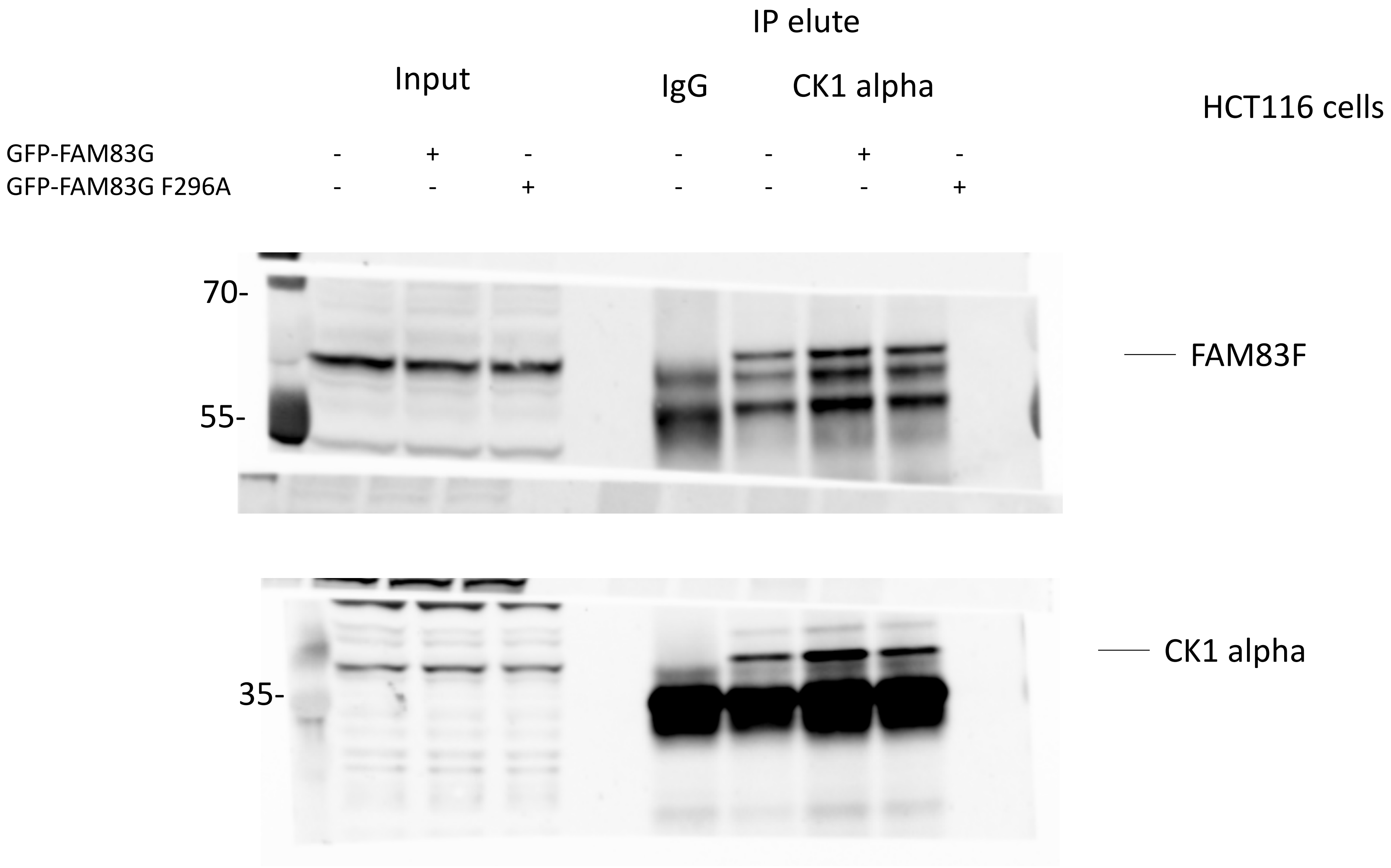
IP elute
Input
IgG
CK1 alpha
HCT116 cells
GFP-FAM83G - + - - - + -
GFP-FAM83G F296A - - + - - - +
70-
55-
FAM83F
35-
CK1 alpha

Figure 5C.

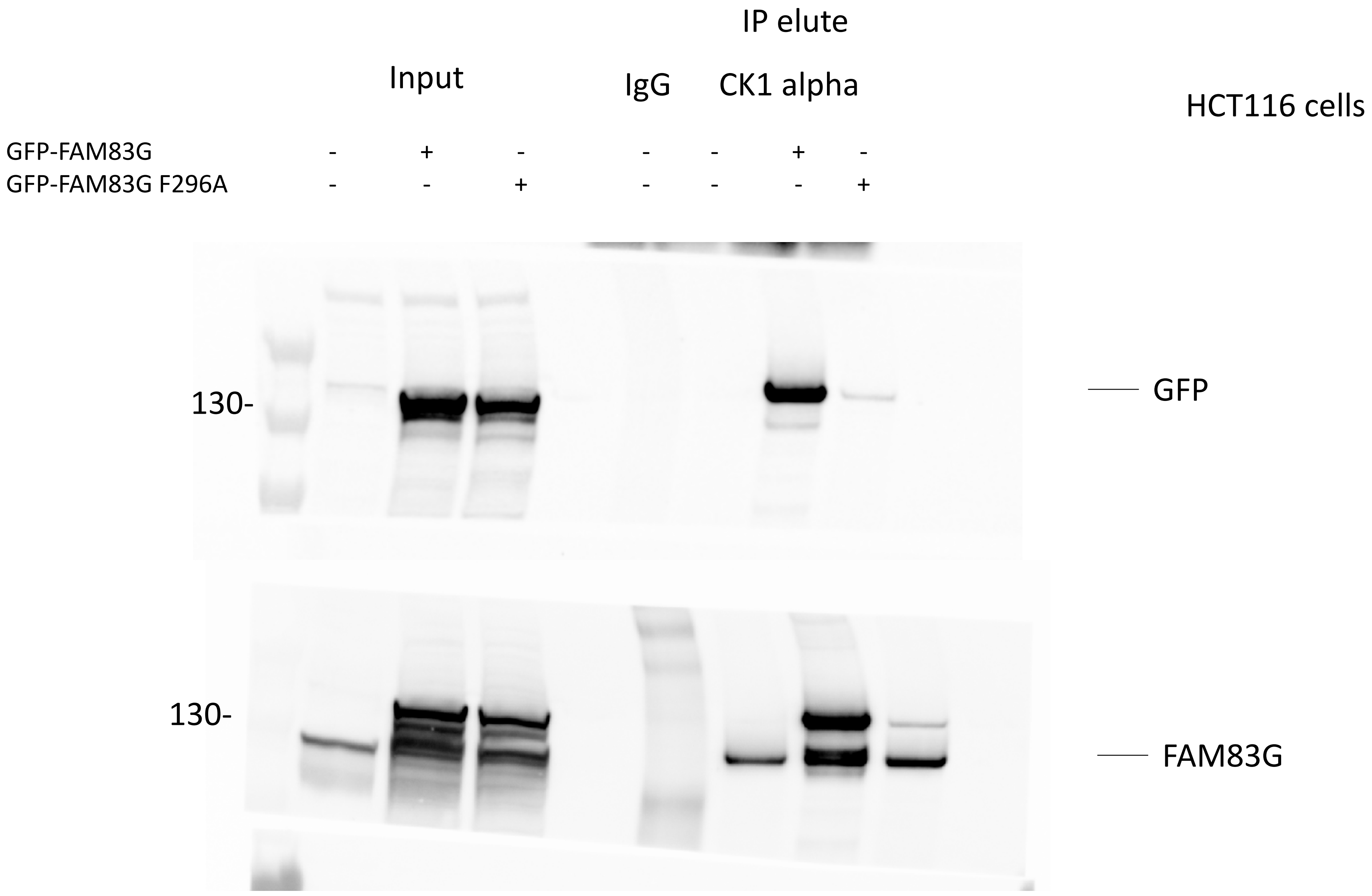

IP elute
Input
IgG
CK1 alpha
HCT116 cells
GFP-FAM83G - + - - - + -
GFP-FAM83G F296A - - + - - - +
130-
GFP
130-
FAM83G

Figure 5C.

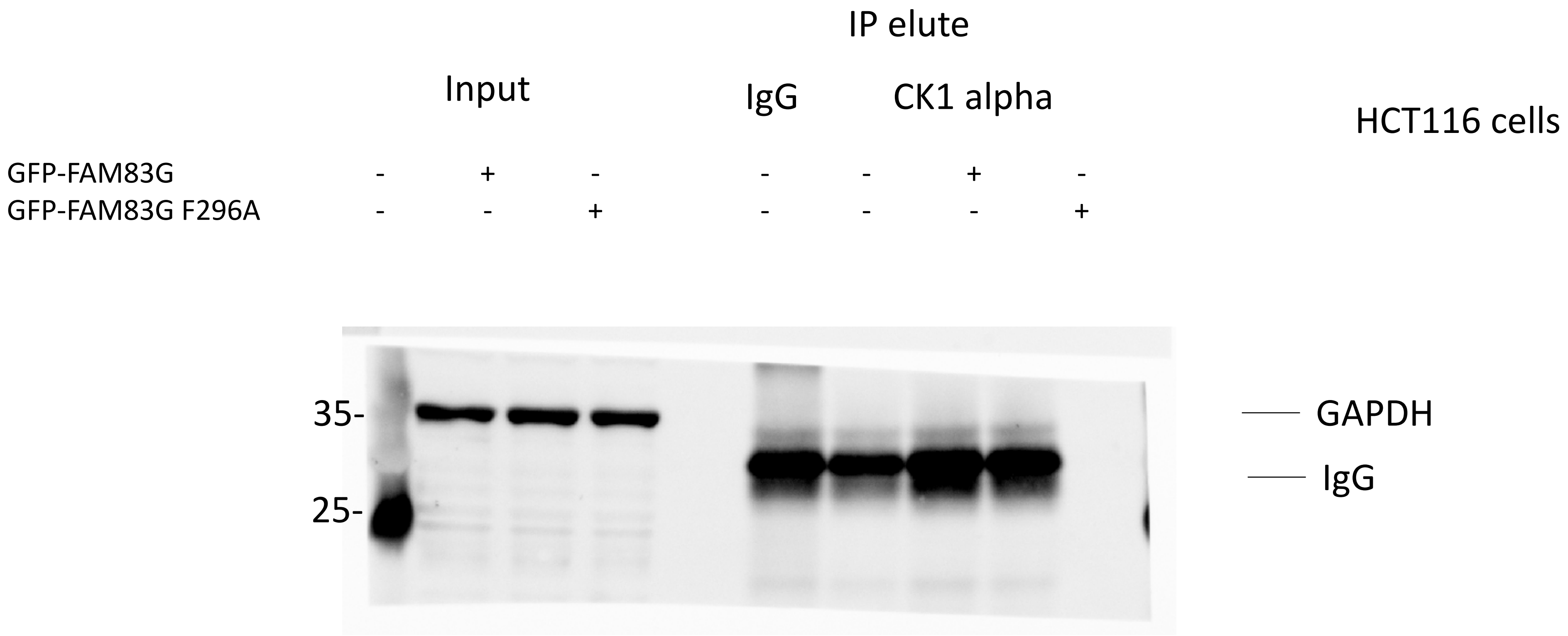
IP elute
Input
IgG
CK1 alpha
HCT116 cells
GFP-FAM83G - + - - - + -
GFP-FAM83G F296A - - + - - - +
35-
25-
GAPDH
IgG

Figure 5C.

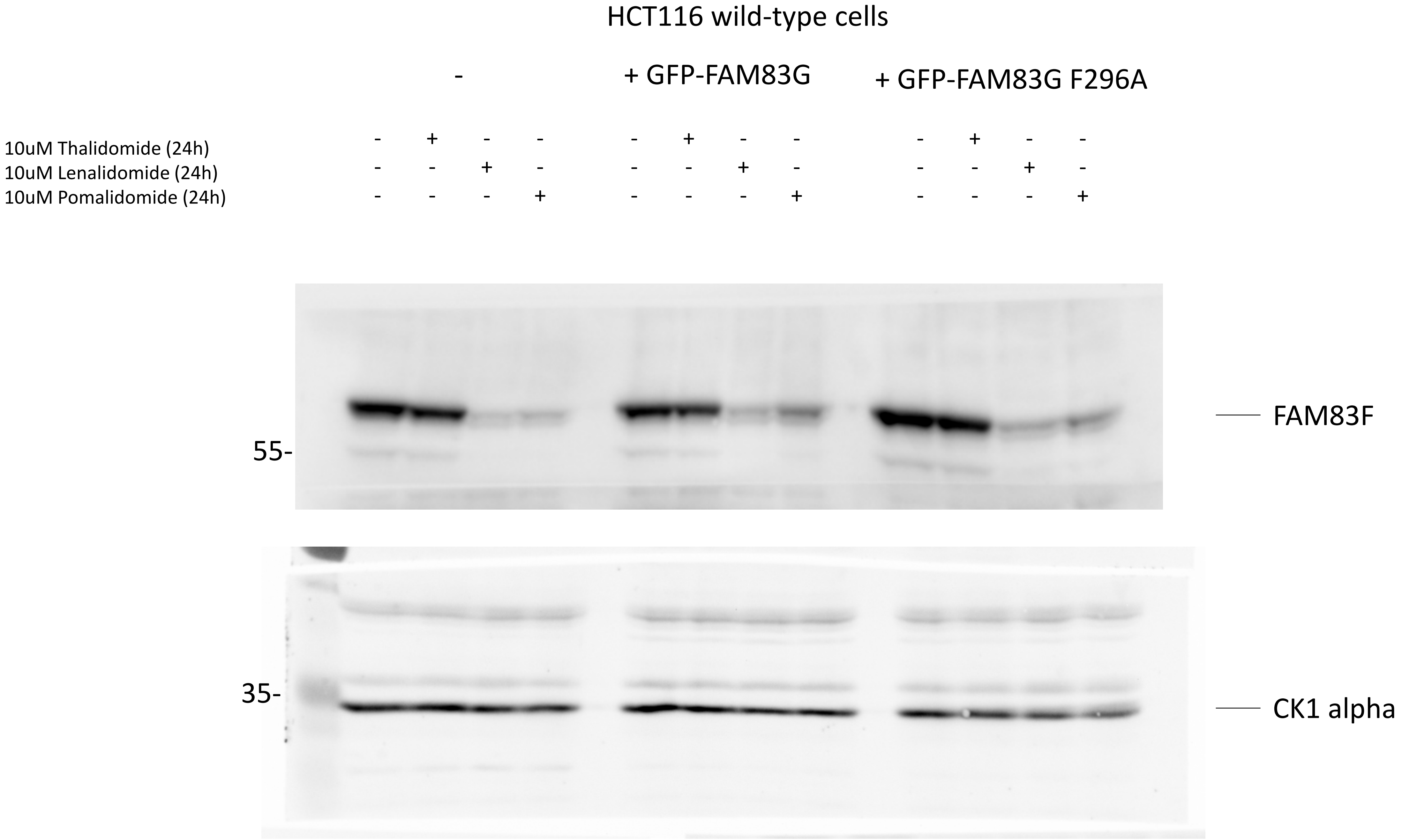
HCT116 wild-type cells
-
+ GFP-FAM83G
+ GFP-FAM83G F296A
10uM Thalidomide (24h)
10uM Lenalidomide (24h)
10uM Pomalidomide (24h)
- + - - - + - - - + - -
- - + - - - + - - - + -
- - - + - - - + - - - +
55-
FAM83F
35-
CK1 alpha

Figure 5C.

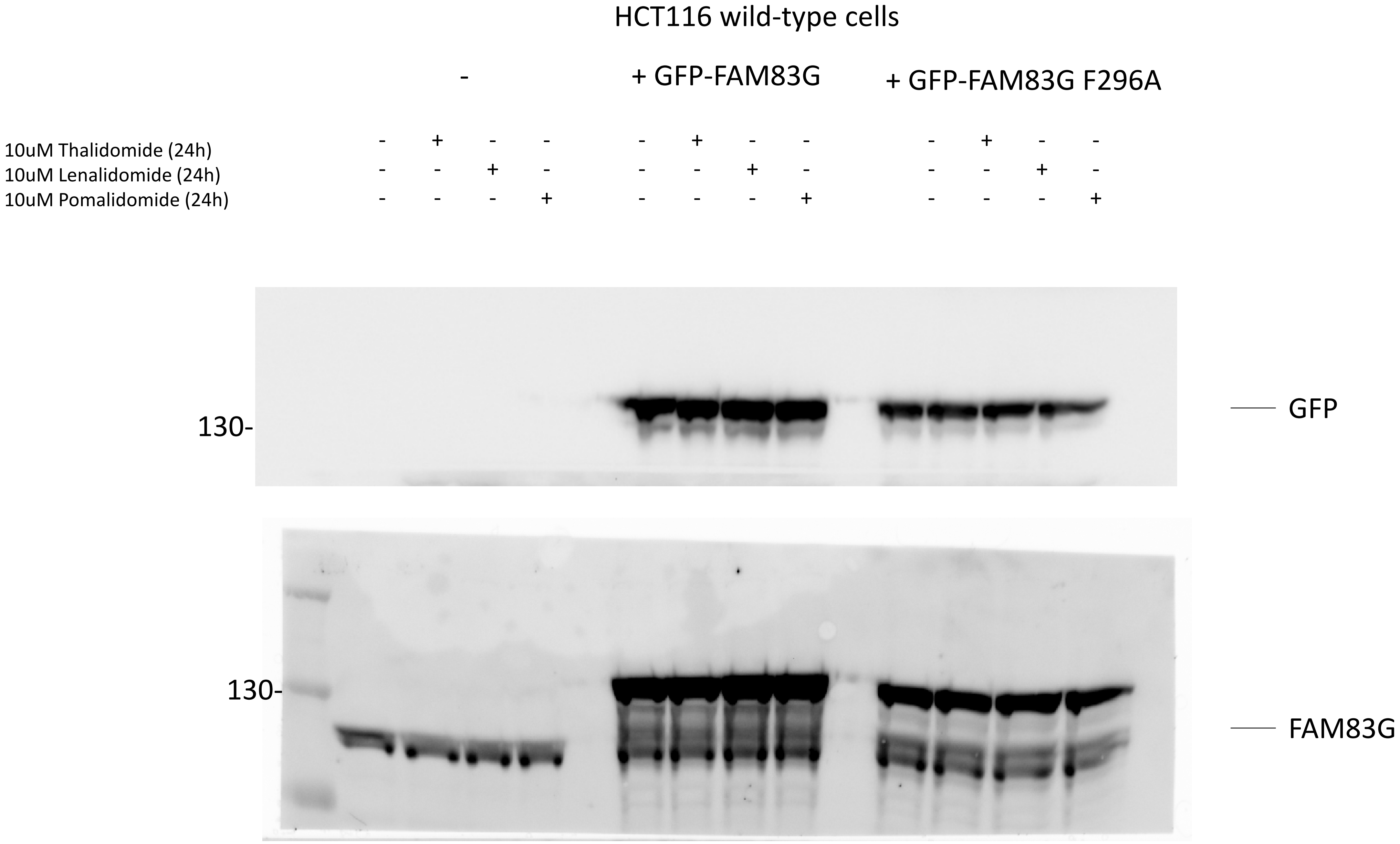

HCT116 wild-type cells
-
+ GFP-FAM83G
+ GFP-FAM83G F296A
10uM Thalidomide (24h)
10uM Lenalidomide (24h)
10uM Pomalidomide (24h)
- + - - - + - - - + - -
- - + - - - + - - - + -
- - - + - - - + - - - +
130-
GFP
130-
FAM83G

Figure 5C.

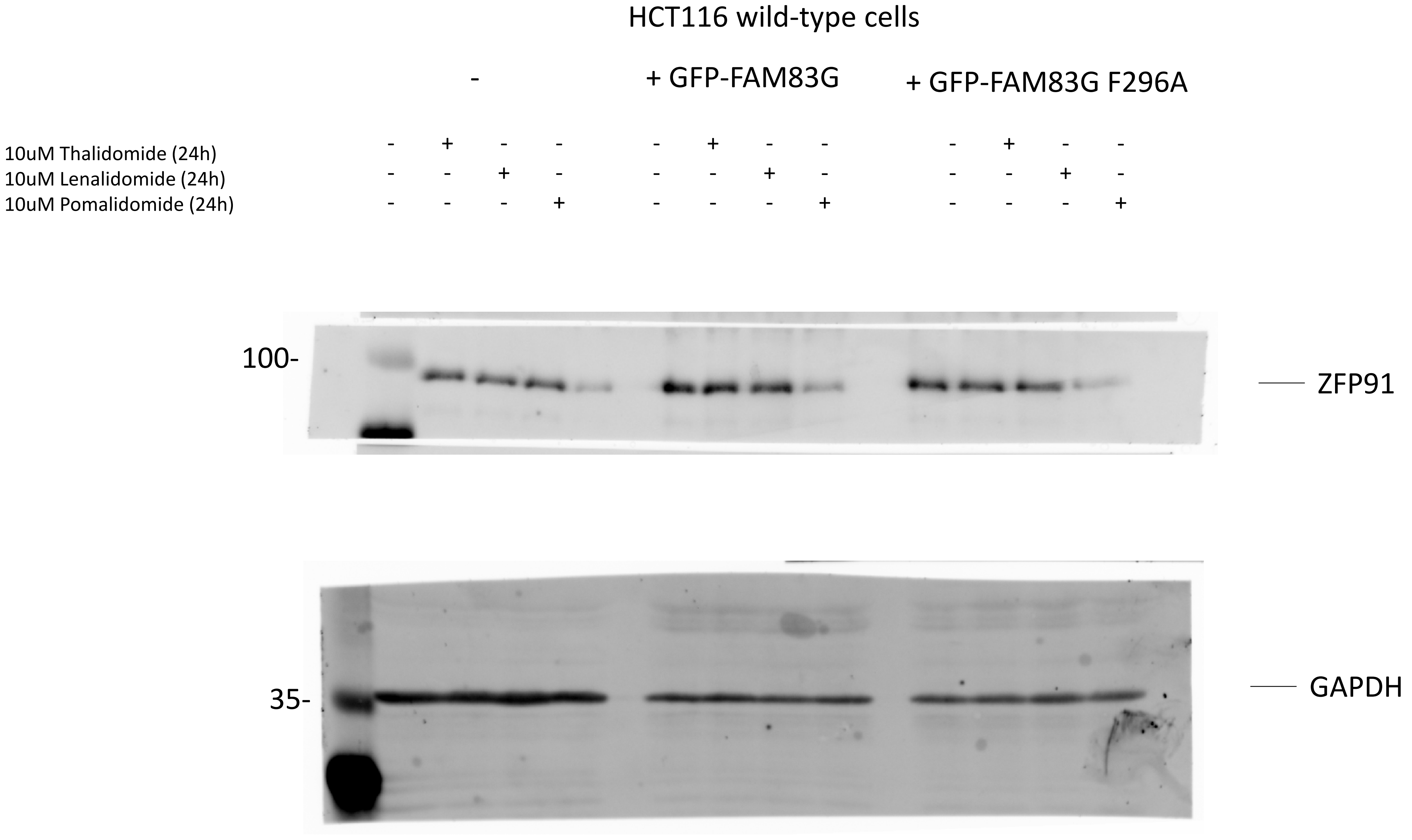
HCT116 wild-type cells
-
+ GFP-FAM83G
+ GFP-FAM83G F296A
10uM Thalidomide (24h) - + - - - + - - - + - -
10uM Lenalidomide (24h) - - + - - - + - - - + -
10uM Pomalidomide (24h) - - - + - - - + - - - +
100-
ZFP91
35-
GAPDH
