# Supplementary material for: IMiDs induce FAM83F degradation via an interaction with CK1α to attenuate Wnt signalling
Source: Life Sci Alliance. 2020 Dec 23;4(2):e202000804. doi: 10.26508/lsa.202000804 (PMC7768194; doi:10.26508/lsa.202000804)
Supplement: Supplementary file 10 [file LSA-2020-00804_SdataF6.pdf]

Figure 6A.

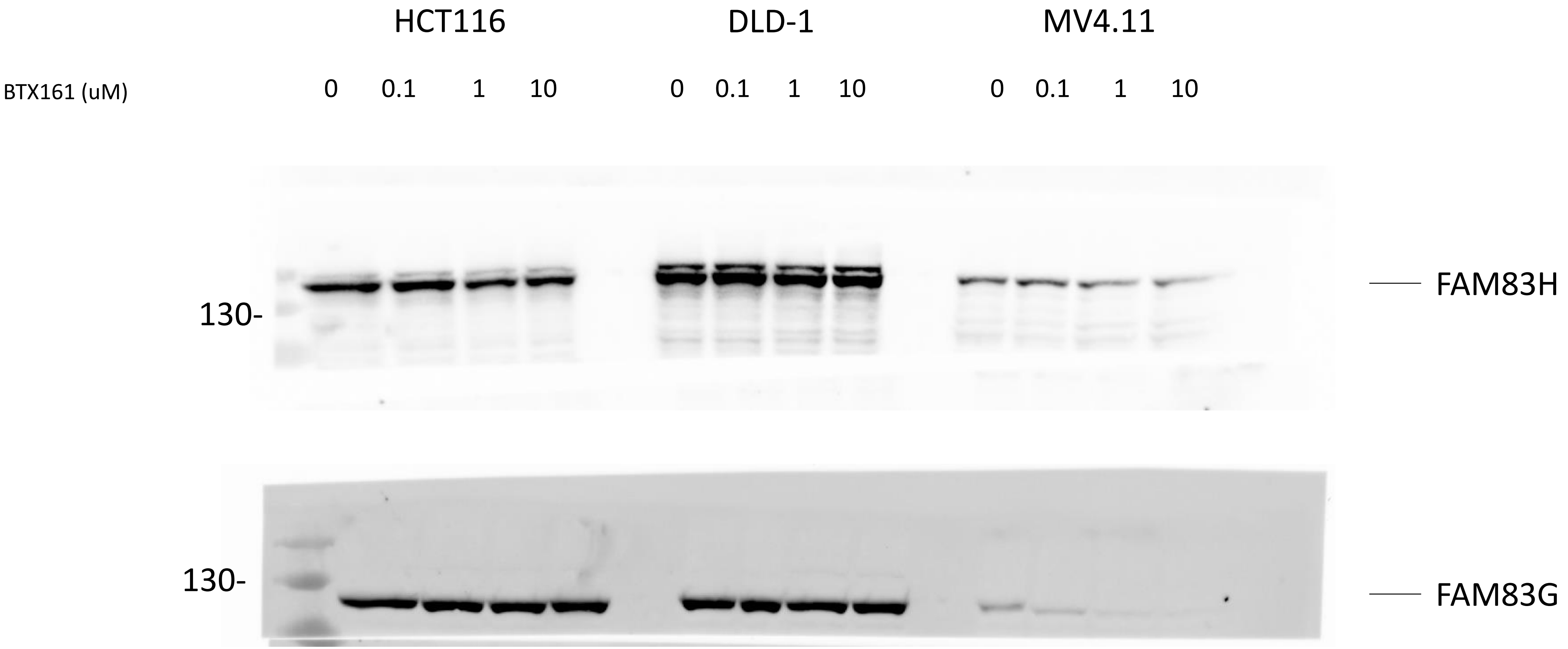
HCT116
DLD-1
MV4.11
BTX161 (uM)
0 0.1 1 10
0 0.1 1 10
0 0.1 1 10
130-
FAM83H
130-
FAM83G

Figure 6A.

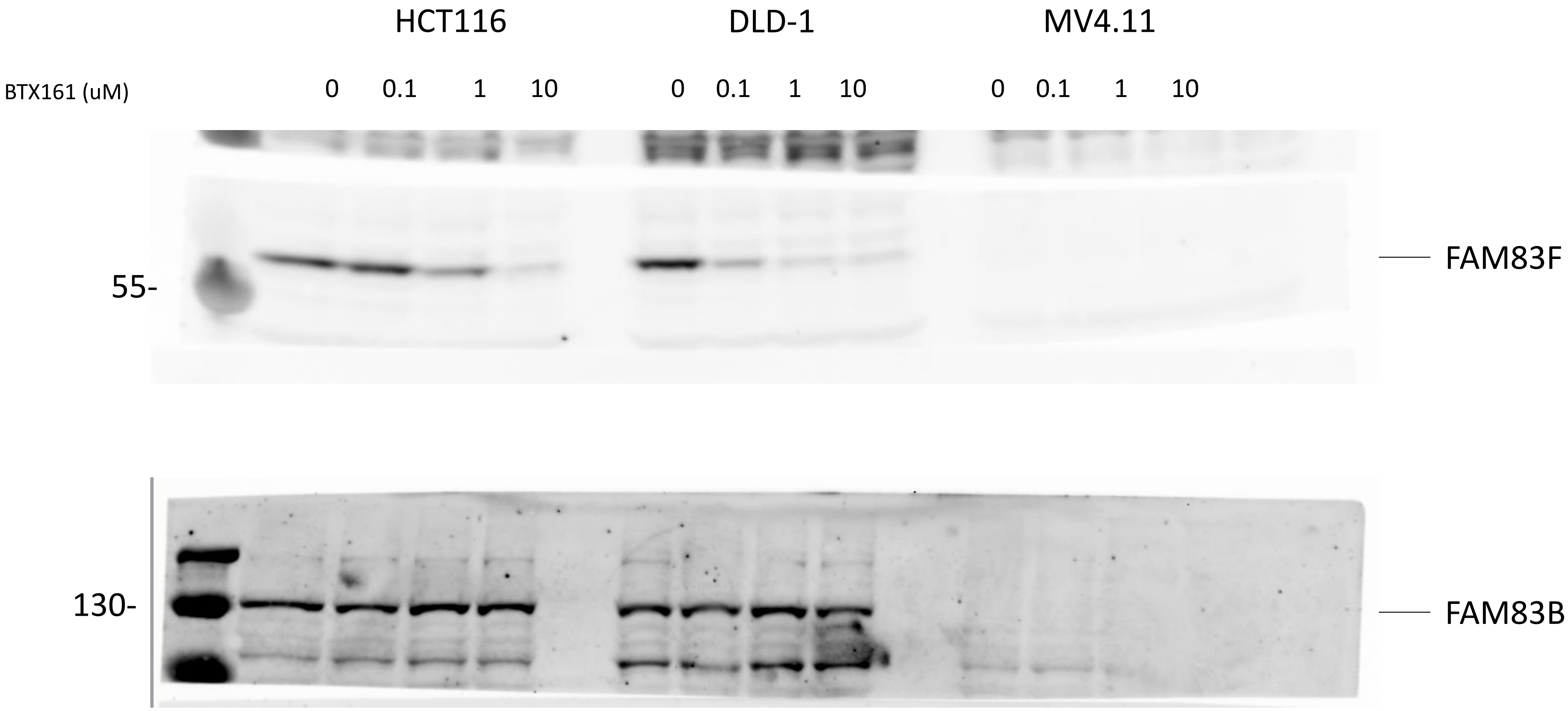
HCT116
DLD-1
MV4.11
BTX161 (uM)
0
0.1
1
10
0
0.1
1
10
0
0.1
1
10
55-
FAM83F
130-
FAM83B

Figure 6A.

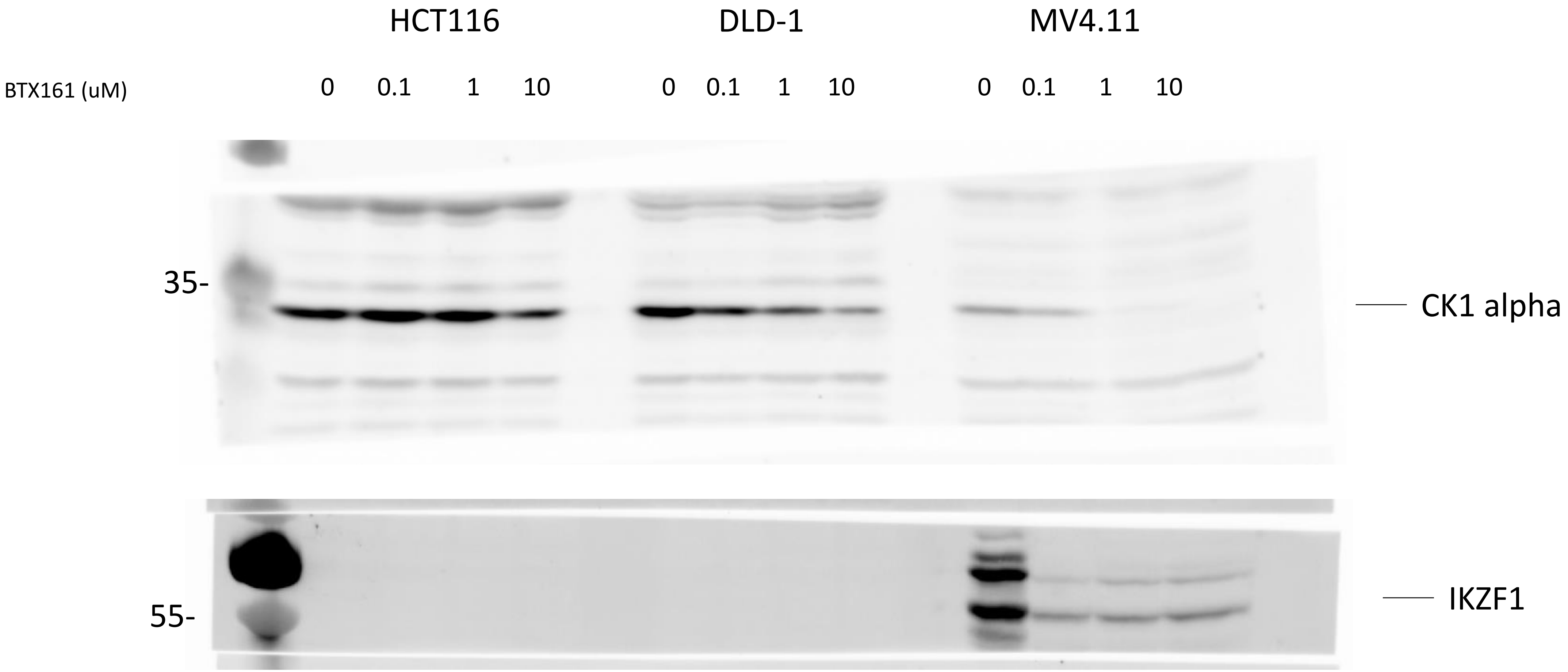

HCT116
DLD-1
MV4.11
BTX161 (uM)
0 0.1 1 10
0 0.1 1 10
0 0.1 1 10
35-
CK1 alpha
55-
IKZF1

Figure 6A.

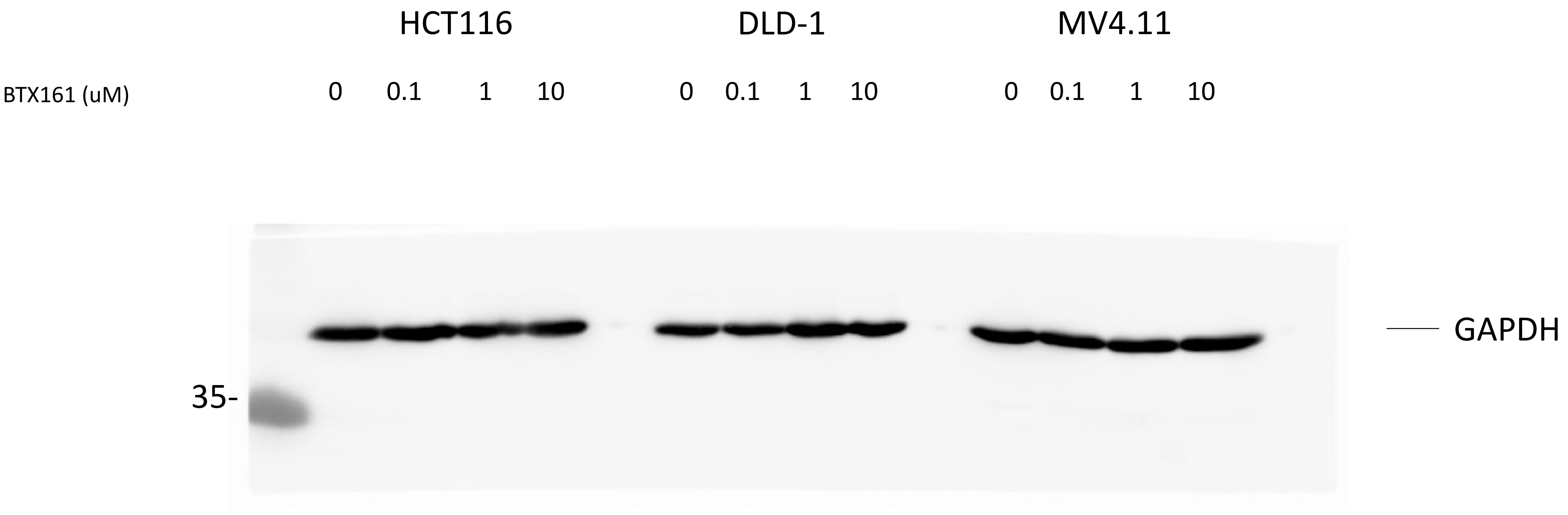

Figure 6B.

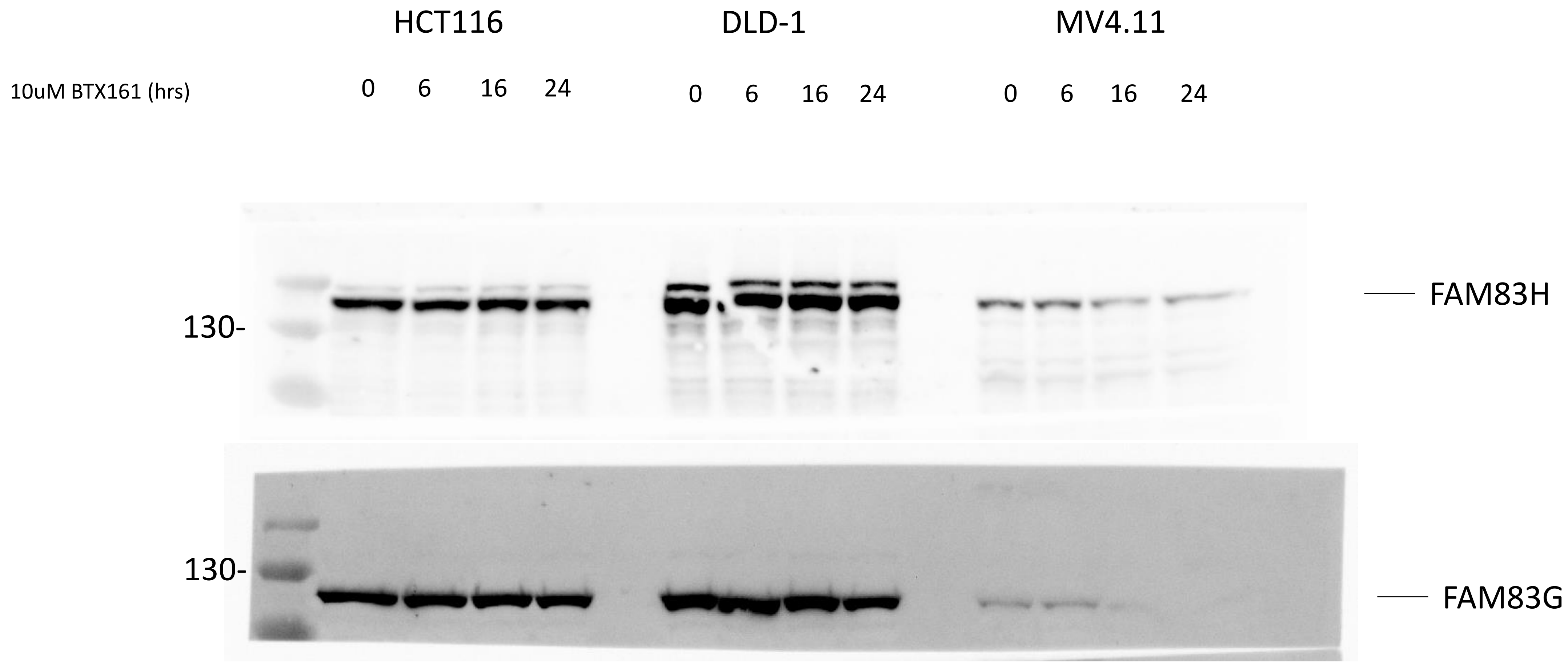
HCT116
DLD-1
MV4.11
10uM BTX161 (hrs)
0
6
16
24
0
6
16
24
0
6
16
24
130-
FAM83H
130-
FAM83G

Figure 6B.

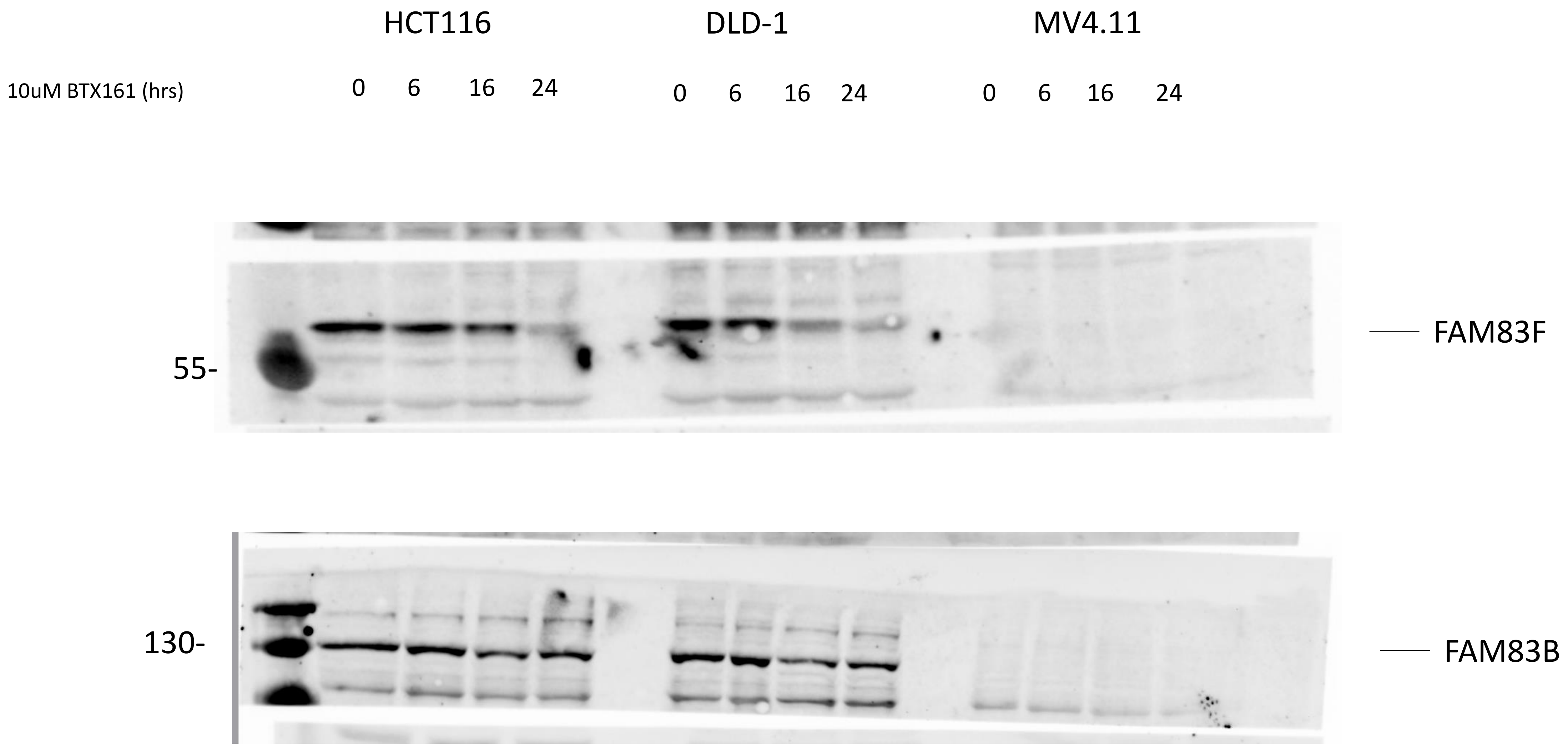
HCT116
DLD-1
MV4.11
10uM BTX161 (hrs)
0 6 16 24
0 6 16 24
0 6 16 24
55-
FAM83F
130-
FAM83B

Figure 6B.

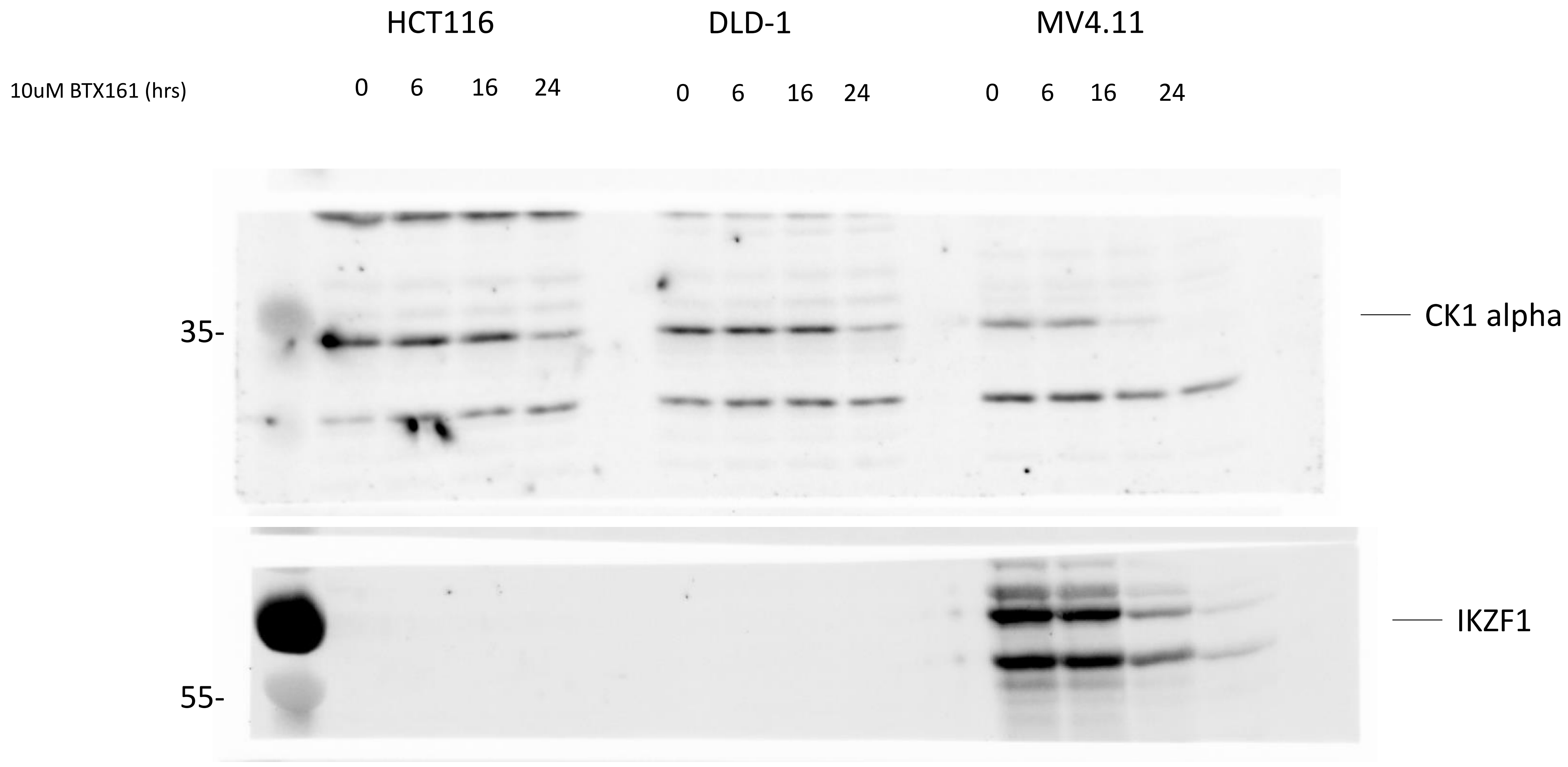
HCT116
DLD-1
MV4.11
10uM BTX161 (hrs)
0 6 16 24
0 6 16 24
0 6 16 24
35-
55-
CK1 alpha
IKZF1

Figure 6B.

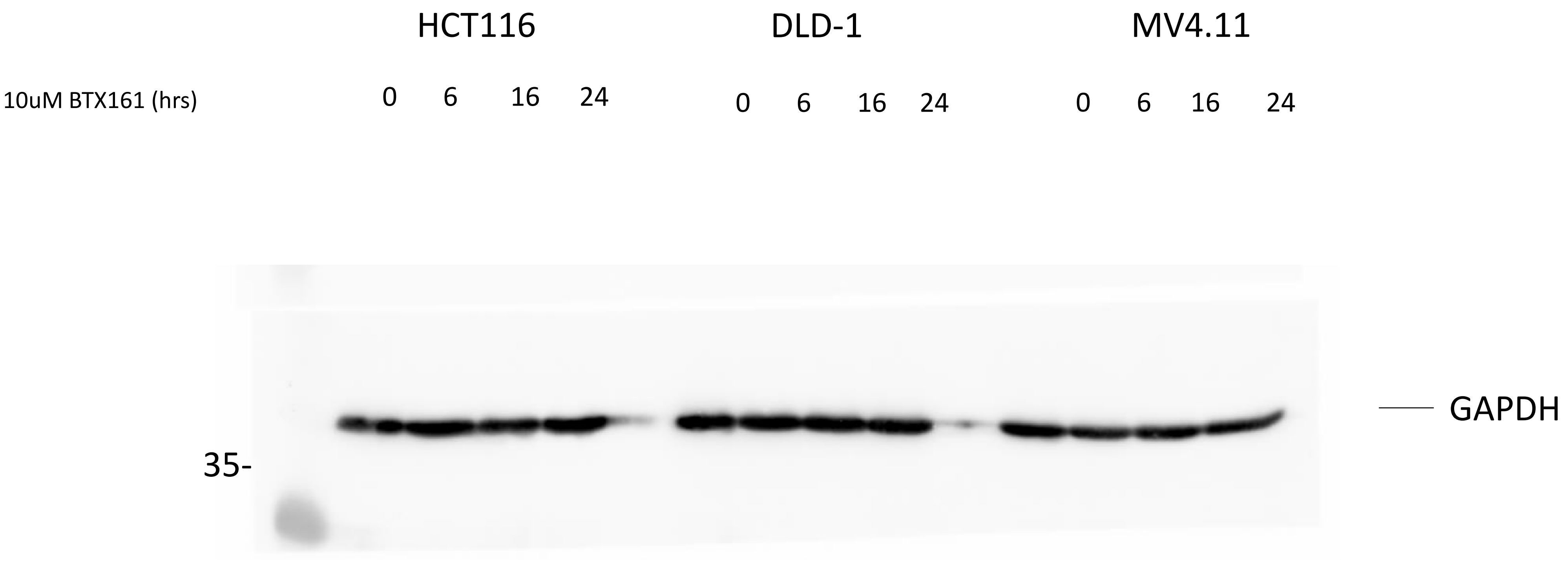

HCT116
DLD-1
MV4.11
10uM BTX161 (hrs)
0 6 16 24
0 6 16 24
0 6 16 24
GAPDH
35-

Figure 6D.

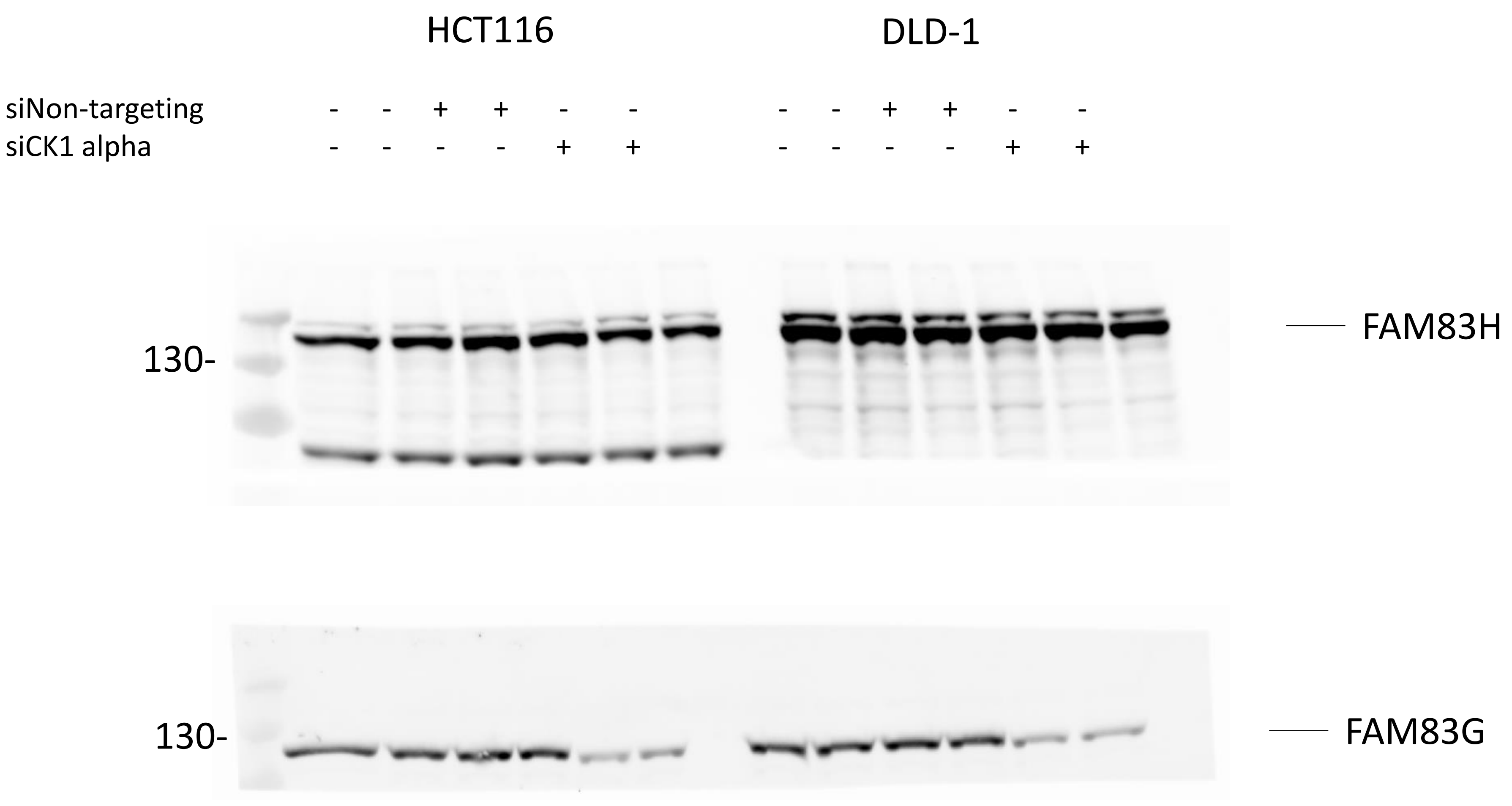
HCT116
DLD-1
siNon-targeting - - + + - - - - + + - -
siCK1 alpha - - - - + + - - - - + +
130-
FAM83H
130-
FAM83G

Figure 6D.

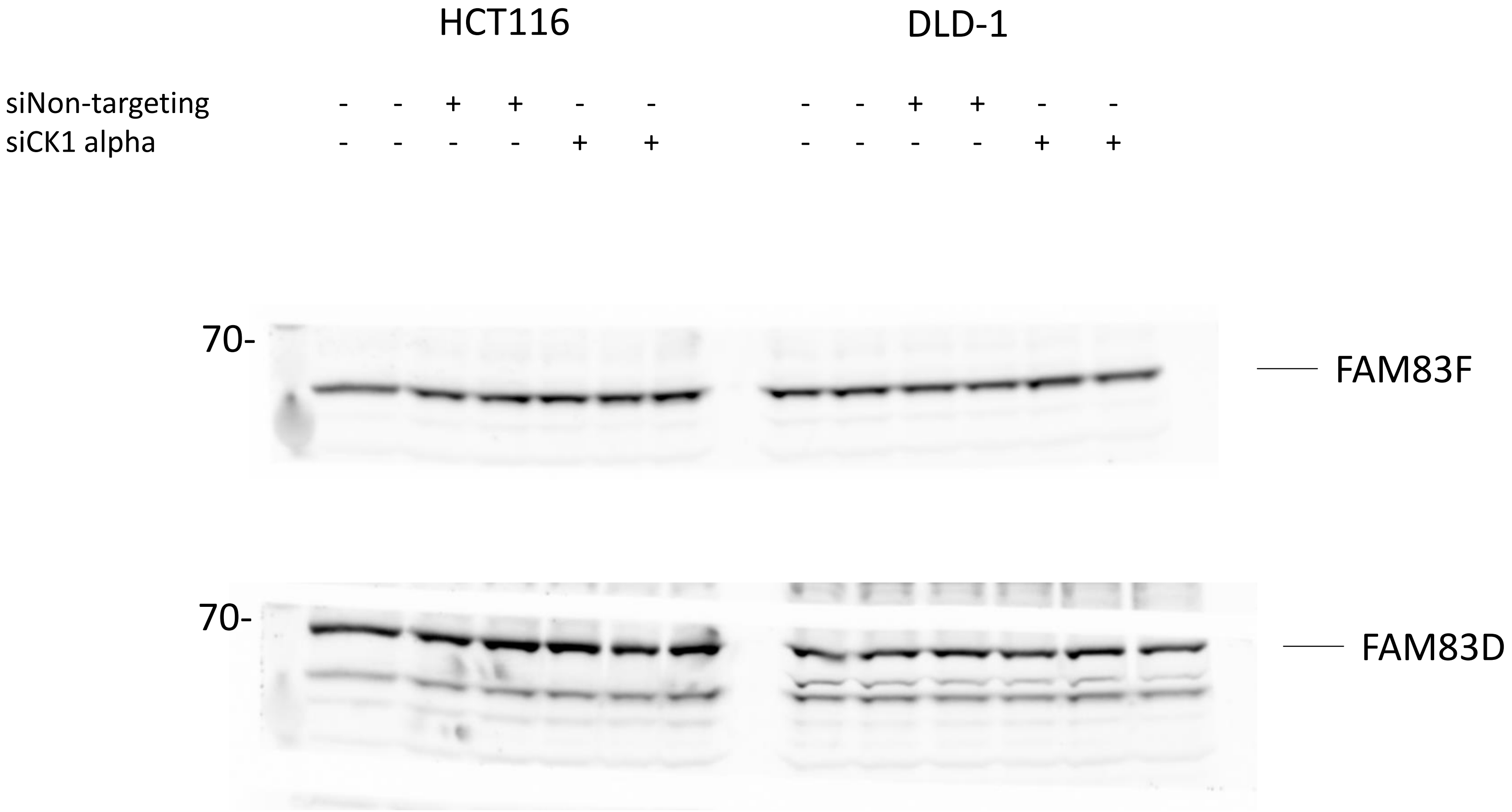
HCT116
DLD-1
siNon-targeting - - + + - - - - + + - -
siCK1 alpha - - - - + + - - - - + +
70-
FAM83F
70-
FAM83D

Figure 6D.

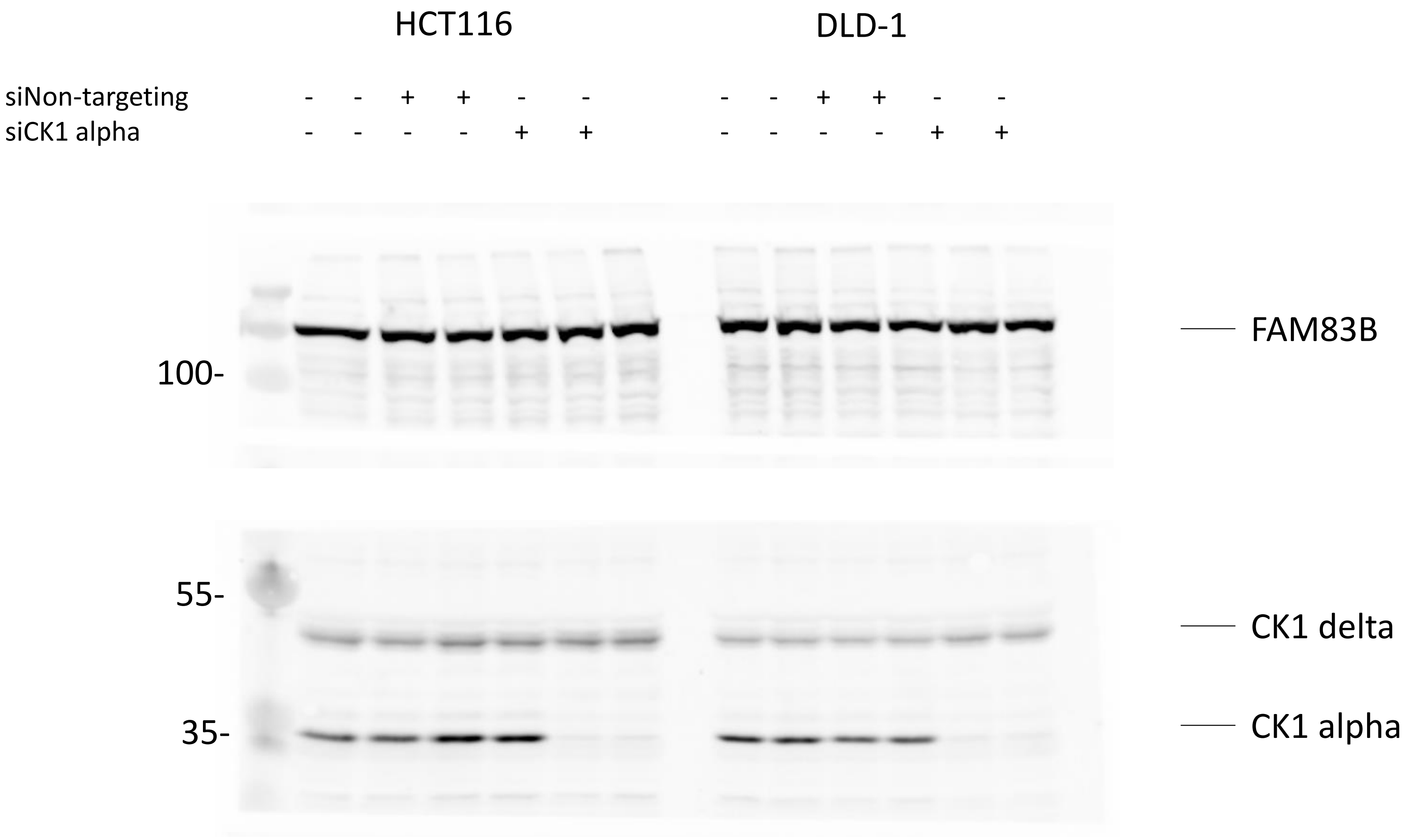

HCT116
DLD-1
siNon-targeting - - + + - - - - + + - -
siCK1 alpha - - - - + + - - - - + +
100-
55-
35-
FAM83B
CK1 delta
CK1 alpha

Figure 6D.

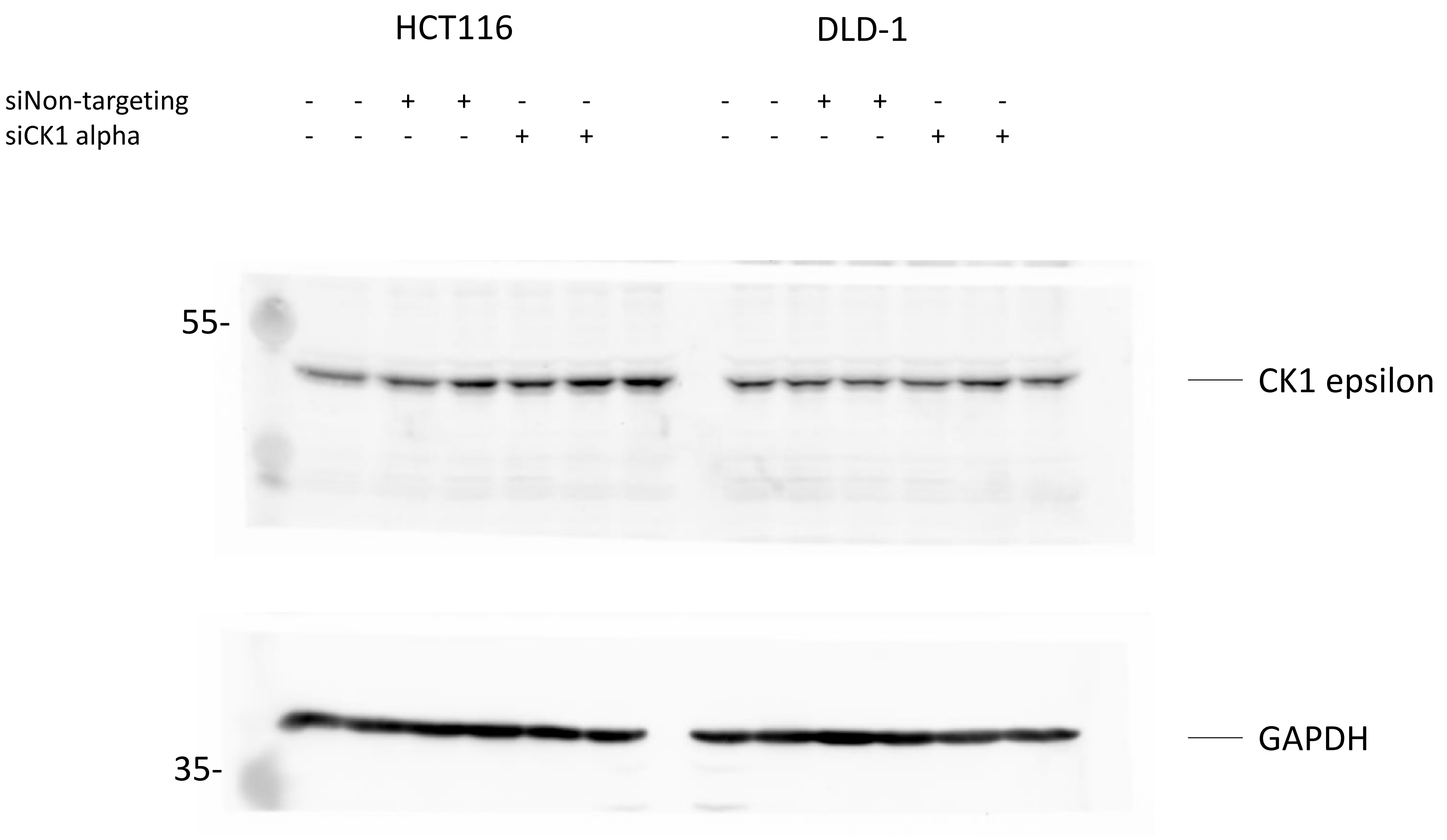
HCT116
DLD-1
siNon-targeting - - + + - - - - + + - -
siCK1 alpha - - - - + + - - - - + +
55-
35-
CK1 epsilon
GAPDH
